# Conformational plasticity of human acid-sensing ion channel 1a

**DOI:** 10.1038/s41594-026-01845-0

**Published:** 2026-07-16

**Authors:** James Cahill, Kimberly A. Hartfield, Stephanie Andrea Heusser, Nadine Ritter, Mette Homann Poulsen, Craig Yoshioka, Stephan Alexander Pless, Isabelle Baconguis

**Affiliations:** 1 Vollum Institute, Oregon Health and Science University, Portland, OR USA; 2 Department of Drug Design and Pharmacology, University of Copenhagen, Copenhagen, Denmark; 3 Department of Biomedical and Clinical Sciences, Linköping University, Linkoping, Sweden; 4 Department of Biomedical Engineering, Oregon Health and Science University, Portland, OR USA

**Keywords:** Cryoelectron microscopy, Ligand-gated ion channels, Sodium channels

## Abstract

Acid-sensing ion channels (ASICs) are typically activated by acidic environments and contribute to nociception and synaptic plasticity. ASIC1a is the most abundant subunit in the central nervous system and forms homomeric channels permeable to Na^+^ and Ca^2+^, making it a compelling therapeutic target for acidotic pathologies including stroke and traumatic brain injury. However, a complete conformational library of human ASIC1a has yet to be described. Here we show that human ASIC1a adopts six major conformations, resolved by cryo-electron microscopy across a pH range between 8.5 and 5.7 and in the presence of a toxin agonist and a gating-modifying amino acid substitution. These major conformations establish linear transmembrane helices to be associated with an open state, delineate mechanistic differences between proton and toxin activation and demonstrate that desensitization involves unexpected conformational diversity in the transmembrane domain. Together, they provide a three-dimensional framework to integrate previous structure–function studies on ASIC.

## Abstract (web summary)

Using cryo-electron microscopy, Cahill, Hartfield et al. identified six structural states of human acid-sensing ion channel 1a, revealing how acidic conditions and peptide toxins regulate channel activation and desensitization through conformational flexibility.

## Main

Acid-sensing ion channels (ASICs) are trimeric proton-gated sodium channels. ASICs are members of the epithelial sodium channel (ENaC)/degenerin family of ion channels and are widely expressed, including in the central and peripheral nervous system. They are involved in important physiological processes such as synaptic transmission^1^, memory^2^ and fear response^3^. ASIC activation is also implicated in ischemic injury during stroke^4,5^ and cardiac infarction^6^ and has roles in pain^7^ and other pathologies, such as epilepsy, anxiety and addiction^8^.

Four genes (*ASIC1*–*ASIC4*) express six ASIC isoforms (ASIC1a, ASIC1b, ASIC2a, ASIC2b, ASIC3 and ASIC4), forming functional homotrimers and heterotrimers^9^. The identified isoforms can all transition across resting closed, activated open and desensitized closed states, with pH sensitivity, desensitization kinetics and ion selectivity differentially affected by subunit composition. ASIC1a activation typically has a pH_50_ of 6.5–6.7 and desensitizes within tens to hundreds of milliseconds.

Structurally, each protomer has a short intracellular N terminus (pre-TM1) that directly precedes the first transmembrane helix (TM1), part of which can form a membrane-embedded reentrant loop^10–12^ containing a conserved HG motif. TM1 is followed by a large proton-sensing extracellular domain (ECD), which contains a set of β-strands that form a so-called ‘molecular clutch’ controlling channel desensitization^13^ and then by TM2. TM2 lines the ion-conducting channel pore and contains a GAS motif that is highly conserved throughout the ENaC/degenerin family and has been implicated as a ‘selectivity filter’ that provides a slight preference to sodium permeability in ASIC1 (refs. ^14–17^). The pore is primarily permeable to sodium and potassium, while calcium can both permeate and inhibit the channel. This permeability profile has possible clinical relevance because of the role of sodium and calcium influx in acidosis-associated brain injury during ischemic stroke^5^.

The potential for pharmacological modulation of ASIC function is apparent from the diversity of naturally occurring toxins targeting ASICs, primarily derived from the venoms of snake, tarantula and sea anemone species with various activating, inhibiting and modulating effects^18^. The binding modes of several such toxins are known from structural characterization using either chicken ASIC1 (refs. ^10,19,20^) or human ASIC1a (ref. ^21^); however, a full description of the ASIC–toxin interaction paradigm would benefit from improved understanding of the structural–functional landscape^18^. For example, the toxin agonist MitTx from *Micrurus* *tener* *tener* binds the closed resting state^7,10^ but it remains unclear whether the opening mechanism and resultant open state conformation differ between channels activated by protons and MitTx. Therefore, modeling this activation mechanism requires structural characterization of closed, MitTx-activated and proton-activated ASIC1.

Understanding of both the physiological function of ASIC and the modulatory potential of pharmacological agents benefits from the characterization of its conformational diversity across resting, conducting and desensitized states. However, it was not clear whether previous structural studies, mostly on chicken ASIC1, fully described a structural landscape that generalizes to human ASIC1a. Using single-particle cryo-electron microscopy (cryo-EM) across a wide pH range, we report the structures of six distinct conformations of human ASIC1a. While we observe a three-step progression from closed to open to desensitized conformations in the ECD, the TM domain (TMD) presents a surprising conformational diversity, including three distinct TMD conformations associated with the desensitized ECD. This ECD-specific and TMD-specific structural diversity is supported by functional experiments and offers crucial insights into the diverse functional landscape of ASIC1a in the nervous system.

## Results

### hASIC1a structural characterization under multiple pH conditions

**Extended Data Fig. 1:**
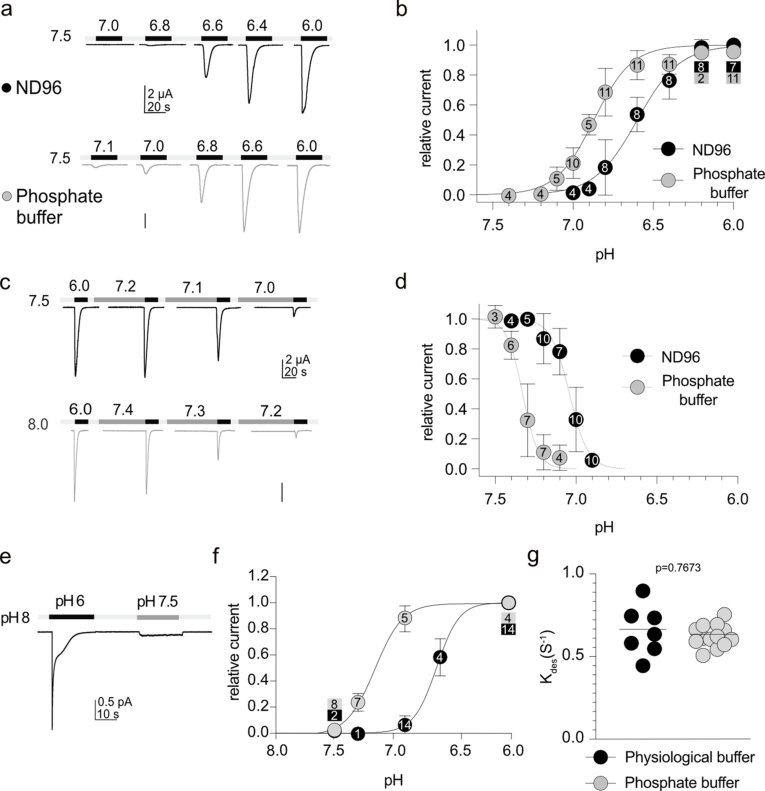
Functional characterization of hASIC1a in phosphate buffer by electrophysiology. **a**. Representative excerpts of two-electrode voltage clamp (TEVC) traces of oocytes expressing hASIC1a activated by decreasing pHs of ND96 (top) or phosphate buffer (bottom). **b**. Normalized activation responses in ND96 and phosphate buffer. **c**. TEVC traces showing the induction of steady-state desensitization by preconditioning the channel for 2 min in decreasing pHs of ND96 (top) or phosphate buffer (bottom) before activation. **d**. Normalized SSD responses in ND96 and phosphate buffer. **e**. Representative trace from HEK293T* cells expressing hASIC1a, recorded using fast perfusion patch clamp electrophysiology in phosphate buffer. The hASIC1a current in phosphate buffer shows the typical fast activation and desensitization at pH 6 and an atypical standing current at pH 7.5. **f**. Normalized activation response of hASIC1a WT expressed in HEK293T* cells, recorded in whole-cell patch-clamp experiments. **g**. Graph summarizing the desensitization rates for hASIC1a in regular physiological solution (black circles; n = 7 cells) and in phosphate buffer (grey circles; n = 13 cells). In a and c, vertical scale bars are 2 μA, all traces are on the same timescale. Data in b, d, and f are mean ± SD; number of biological replicates are indicated inside the circles, or in adjacent color-coded boxes where space did not permit. Statistical analysis: two-sided non-parametric t-test (Mann Whitney).

Structural characterization of ASIC1 under different pH conditions has previously relied on individually selected chemical environments (for example, Tris at higher pH^12,13,21,22^ or sodium acetate at low pH^10,19,20^), complicating interpretation of whether structural differences were proton mediated or artifactual. Instead, we chose a sodium phosphate buffering system that can buffer solutions in a comparatively wide pH range. The effect of sodium phosphate on hASIC1a was evaluated by determining activation and steady-state desensitization (SSD) curves in a simple buffer of phosphate and sodium chloride and in a more physiological buffer (ND96; HEPES based) (Extended Data Fig. 1a–d). Both buffer systems resulted in pH-dependent responses in *Xenopus* oocytes and HEK cells heterologously expressing hASIC1a, although the pH_50_ values in phosphate buffer were shifted in the alkaline direction for activation (ΔpH 0.27 and 0.53 pH units for *Xenopus* oocytes and HEK cells, respectively) and SSD (ΔpH 0.29 units for *Xenopus* oocytes) (Extended Data Fig. 1 and Supplementary Table 1). In HEK cells, we observed a small, sustained current induced by phosphate buffer at pH 7.5 (Extended Data Fig. 1e), providing functional evidence for channel opening at this pH. This alkaline shift is likely because of calcium sequestration or precipitation by phosphate, which reduces ambient free Ca^2+^, thereby favoring channel activation^23,24^.

**Fig. 1:**
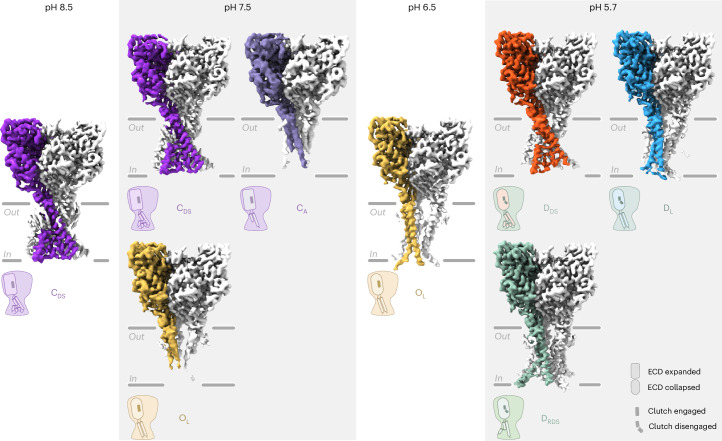
Conformational plasticity of hASIC1a by pH modulation. Maps of hASIC1a are arranged by the pH at which the samples were prepared. Map regions corresponding to an individual hASIC1a subunit are colored; colors are coded by conformation. The orientation and approximate position of the homotrimer within the plasma membrane are indicated with gray lines. Cartoon schematics are shown beneath the corresponding map and represent the major morphological features of TMD conformation, acidic pocket conformation, clutch orientation and apparent role in the mechanistic cycle (color of background shape: purple for resting, gold for conducting and green for desensitized) (Extended Data Figs. 1–3).

**Extended Data Fig. 2:**
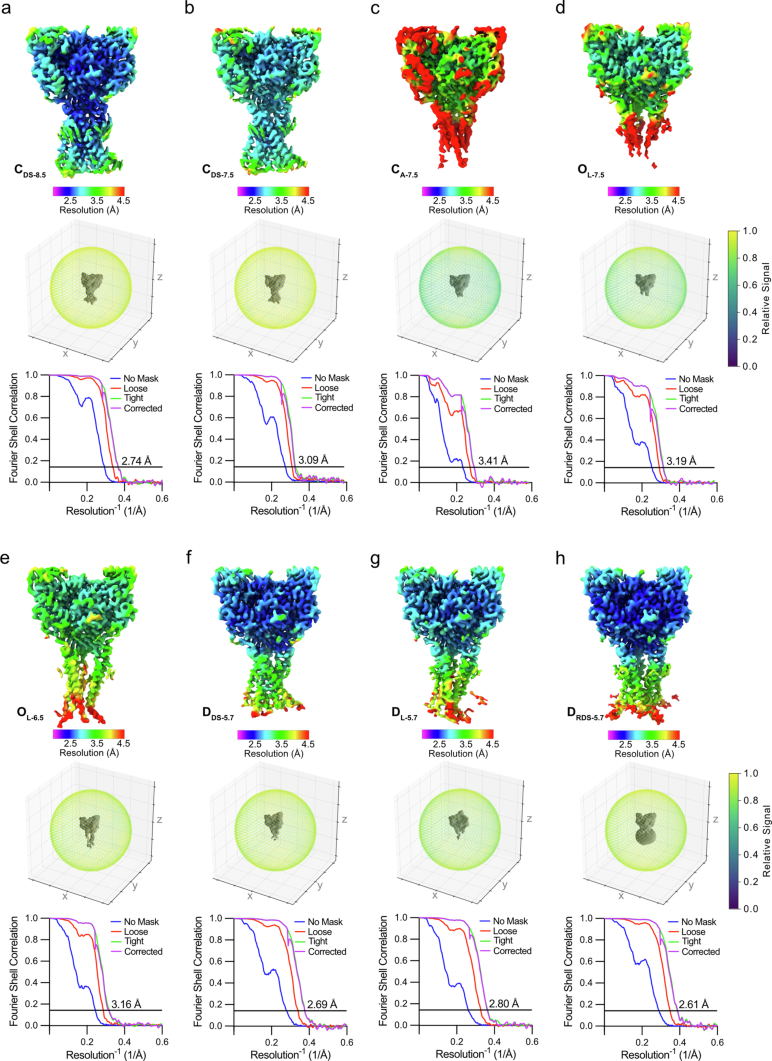
Cryo-EM data processing of hASIC1a maps attained with pH modulation. **a-h**. Local resolution estimation (top), orientation distribution (middle), and Fourier shell correlation (FSC) (bottom) for each indicated map. The resolution at the FSC = 0.143 cutoff (black line) is indicated.

The conformational landscape of hASIC1a was studied under four pH conditions using single-particle cryo-EM: pH 8.5, 7.5, 6.5 and 5.7. Full-length hASIC1a was expressed in HEK293S cells and purified in digitonin-containing phosphate buffer. We solved eight structures of wild-type hASIC1a, giving rise to six different conformations (Fig. 1, Extended Data Fig. 2, and Supplementary Tables 2 and 3). The conformations were differentiated by key structural elements summarized in Table 1. For clarity, we label the structures with initialisms according to the functional state associated with their ECD conformation (closed, open or desensitized) and TM architecture (for example, domain swapped or linear); the latter descriptor and pH, where relevant, are denoted in subscript.

**Table 1:** Summary of hASIC1a conformations

Conformation	Acidic pocket	Clutch	TMD arrangement	TM2b domain swap?	Reentrant pre-TM1?	Similar to (PDB ID)
C_DS_	Expanded	Engaged	Twisted	Yes	Yes	5WKU, 5WKV, 6AVE (ref. ^13^); 5WKX, 5WKY (ref. ^22^); 6VTL (ref. ^12^); 7CFS, 7CFT (ref. ^21^); 6X9H (ref. ^26^); 7RNN (ref. ^25^)
C_A_	Expanded	Engaged	Untwisted	?	?	
O_L_	Collapsed	Engaged	Untwisted	No	?	4FZ1 (ref. ^19^); 6L6N^a^
D_DS_	Collapsed	Disengaged	Twisted	Yes	Yes	4NYK (refs. ^10,43^); 6CMC (ref. ^22^); 6VTK (ref. ^12^)
D_L_	Collapsed	Disengaged	Untwisted	No	?	2QTS (ref. ^11^); 3S3X, 3S3W (ref. ^20^); 4FZ0 (ref. ^19^)
D_RDS_	Collapsed	Disengaged	Untwisted	Yes	?	
O_M_	Collapsed	Engaged	Untwisted	No	?	
C_T26V_	Expanded	Engaged	Untwisted	No	?	6L6I^a^
O_M-RDS_^b^	Collapsed	Engaged	Untwisted	Yes	?	4NTW, 4NTX, 4NTY (ref. ^10^)

^a^ PDB 6L6I and 6L6N are hASIC1a crystal structures with no associated publication and are included here for completeness. The categorization of PDB 6L6I with C_T26V_ is based on structural similarity, as the T26V substitution is not known to be present in PDB 6L6I.

^b^ The conformation named O_M-RDS_ is known from MitTx–cASIC1 structural characterization but was not detected in this work. It is included for completeness and because this conformation is discussed as it relates to D_RDS_ and O_M_, characterized in the current work.

**Extended Data Fig. 3:**
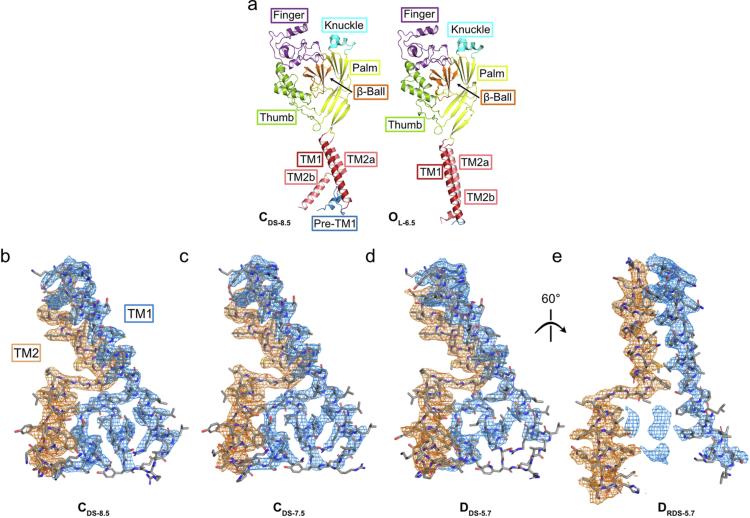
Structural overview of hASIC1a. **a**. Cartoon representation of (left) C_DS-8.5_ and (right) O_L-6.5_ with color-coding according to the ‘hand holding a ball’ model of subdomain architecture. **b-e**. Map regions corresponding to TM1/pre-TM1 (blue) and TM2 (orange) are shown in mesh representation over the model in stick representation. For e, unmodeled map features in the approximate expected position of the pre-TM1 ‘reentrant loop’ are shown.

The first conformation, identified at pH 8.5 and 7.5, resembles closed, resting-state structures of cASIC1 and hASIC1a at high pH^12,21,25,26^. The three hASIC1a protomers exhibit three-fold rotational symmetry around the putative pore axis. Each protomer’s ECD has a characteristic ‘hand holding a ball’ architecture (Extended Data Fig. 3a). A relative expansion of the ECD has been attributed to electrostatic repulsion around a pocket of deprotonated acidic side chains^13^. The six TM helices form three parallel pairs, which twist around the pore axis. In this conformation, TM2 is domain swapped; the GAS motif unwinds to abut the proximal half (TM2a) of one protomer to the distal half (TM2b) from another protomer. Pre-TM1 forms a membrane-embedded ‘reentrant loop’ (Extended Data Fig. 3b,c), networking the HG and GAS motifs to stabilize the domain-swapped conformation^12^. We denote this conformation as C_DS_ (Table 1).

In addition to C_DS_, a second conformation was observed at pH 7.5. The ECD conformation closely resembles C_DS_ (Extended Data Fig. 3a); however, the pairs of TM helices are not twisted around the pore axis as in C_DS_. Although TM1 is resolved for a longer distance than TM2, pre-TM1 is not resolved and its conformation cannot be determined. Because the GAS motif and TM2b cannot be resolved, we are unable to determine whether TM2 domain swapping occurs in this conformation; thus, we categorized it as an ‘ambiguous’ TMD conformation (C_A_).

A third major conformation was observed at both pH 7.5 and 6.5. Relative to both C_DS_ and C_A_, the ECD is in a more contracted configuration. This conformational change has been associated with protonation of the pocket of acidic side chains and correlated with channel activation^11^. The TMD has untwisted TM helices as in C_A_; however, the helices are less closely spaced. This conformation has TM2a and TM2b forming a continuous, linearized TM2 helix. Therefore, we refer to this confirmation as O_L_, in line with functional evidence of open channels at this pH (Extended Data Fig. 1). Pre-TM1 cannot be resolved and the close positioning of the six TM helices does not leave a space for a pore-lining reentrant loop. The TMD exhibits slight asymmetry around the three-fold axis of the ECD; as a result, O_L_ and all conformations lacking domain-swapped TM2b helices (including C_A_) were analyzed using *C*_1_ symmetry instead of the *C*_3_ symmetry used for domain-swapped conformations such as C_DS_ (Supplementary Table 3).

Three distinct conformations were observed at pH 5.7. Their ECDs resemble that of O_L_ in most respects. However, the characteristic inversion (‘disengagement’) of the β11–β12 ‘clutch’ linker, proposed to decouple the acid-sensing ECD from the pore-forming TM2 (ref. ^13^), indicates that the protein has shifted into a desensitized state. These three hASIC1a conformations differ in TMD configuration. D_DS_ has a twisted, domain-swapped TMD configuration closely resembling the TMD in C_DS_, with reentrant loop pre-TM1 (Extended Data Fig. 3d). D_L_ has an untwisted TMD and linearized TM2, with a tilted TMD relative to the pseudo-three-fold axis of the ECD that recalls a desensitized cASIC1 structure with dramatically tilted TMD^11^. D_RDS_ has a unique TM configuration sharing some features of both D_DS_ and D_L_; it features an untwisted TMD and domain-swapped TM2. Its name reflects that its TM helices are rotationally displaced relative to the conformation of D_DS_ and C_DS_. Pre-TM1 is not modeled in D_RDS_, although there are weak map features beneath the GAS motif and between TM1 and TM2b consistent with the reentrant loop position in C_DS_ and D_DS_ (Extended Data Fig. 3e).

### MitTx-bound hASIC1a adopts a linearized TMD conformation

**Fig. 2:**
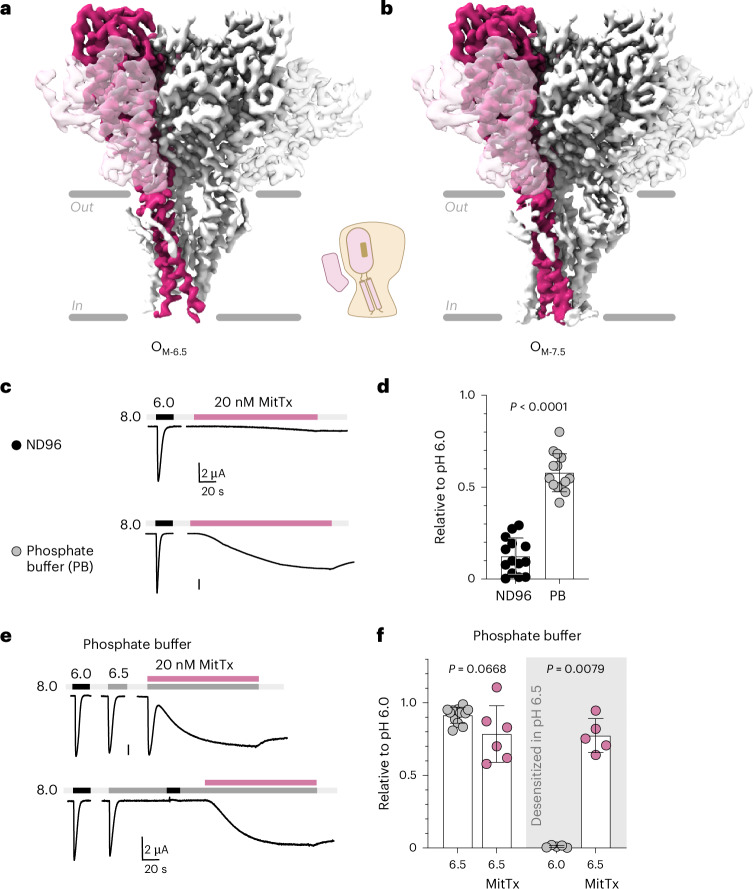
Modulation of hASIC1a by MitTx in phosphate buffer. **a**,**b**, Maps of O_M-6.5_ and O_M-7.5_. Map regions corresponding to an individual hASIC1a subunit are colored. Map regions corresponding to MitTx are shown in transparent representation. The orientation and approximate position of the homotrimer within the plasma membrane are indicated with gray lines. A cartoon schematic is shown between the two maps. **c**, TEVC traces showing the effect of 20 nM MitTx in ND96 (top) and phosphate buffer (bottom) at pH 8.0. Vertical scale bars are 2 μA; traces are on the same timescale. **d**, Normalized response of data shown in **c** comparing the response to 20 nm MitTx in ND96 (*n* = 14 oocytes) and phosphate buffer (*n* = 14 oocytes). **e**, TEVC traces showing the effect of 20 nM MitTx in phosphate buffer when applied at pH 6.5 (top) and when channels were preconditioned at pH 6.5 before MitTx addition (bottom). **f**, Normalized responses of data from oocytes shown in **e**. Responses to MitTx are reported at the plateau. Four experimental conditions were examined: pH 6.5 alone (*n* = 12), pH 6.5 in the presence of MitTx (*n* = 6), channels first desensitized at pH 6.5 followed by application of pH 6.0 (*n* = 5) and channels first desensitized at pH 6.5 followed by the addition of MitTx (*n* = 5). For **d**,**f**, data are the mean ± s.d.; each data point represents a biological replicate. Statistical analysis was conducted using a two-sided nonparametric *t*-test (Mann–Whitney) (Extended Data Fig. 4). Source data

**Extended Data Fig. 4:**
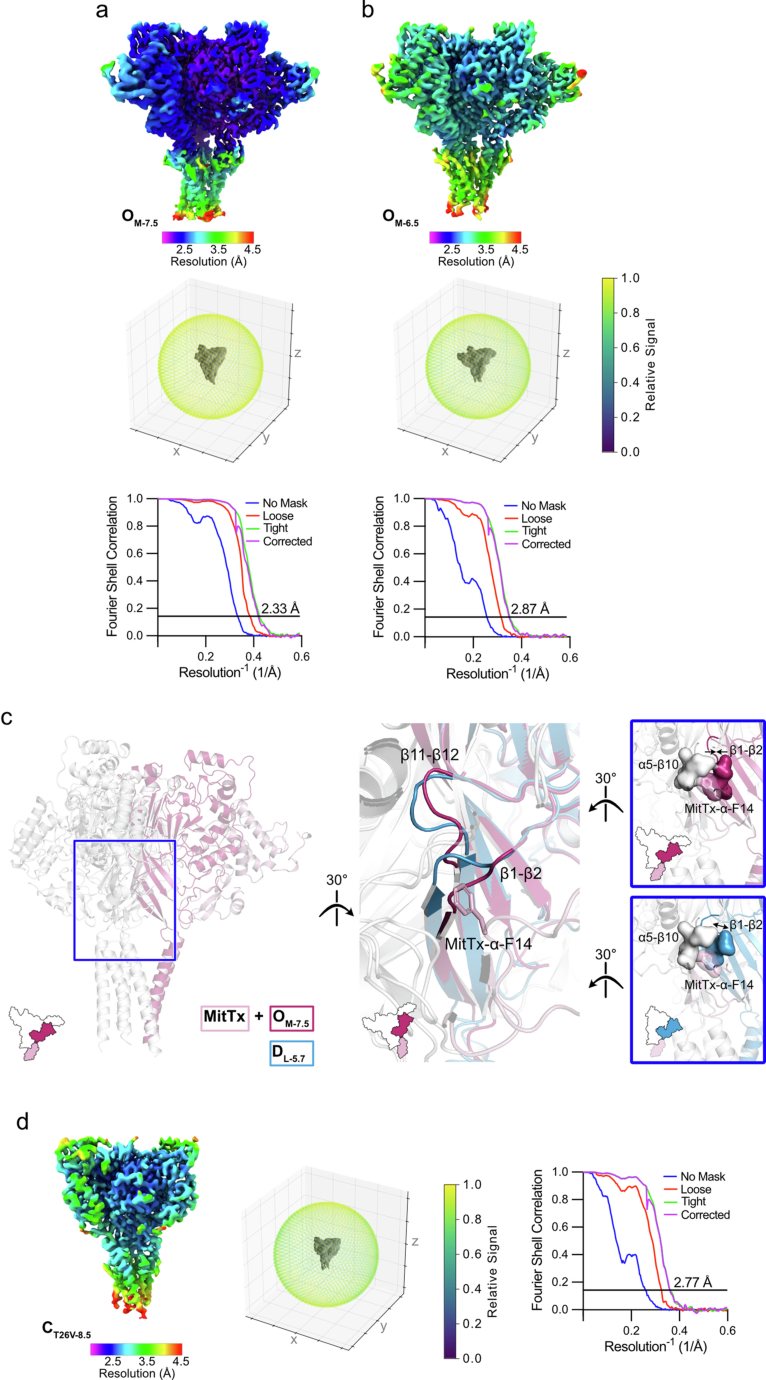
Cryo-EM data processing of hASIC1a maps attained using toxin addition or mutagenesis. **a-b**. Local resolution estimation (top), orientation distribution (middle), and Fourier shell correlation (bottom) for the maps of hASIC1a in complex with MitTx at pH 6.5 and 7.5. The resolution at the FSC = 0.143 cutoff (black line) is indicated. **c**. Left panel: Reference image of O_M-7.5_ with a highlighted region corresponding to panels on the right. Middle panel: The positioning of MitTx-α-Phe14 in the model of O_M-7.5_ is shown relative to the β1-β2 linkers of the minor-interacting hASIC1a in the models of O_M-7.5_ (magenta) and D_L-5.7_ (blue). Right panel top: The packing of the MitTx-α-Phe14 side chain (pink) between the β1-β2 linker of the minor-interacting hASIC1a subunit (magenta) and the α5-β10 loop of the major-interacting hASIC1a subunit (white) is represented with surface over a cartoon representation. Arrows indicate the proximity of the two hASIC1a subunits within the depicted region. Right panel bottom: The position of the MitTx-α-Phe14 side chain from the aligned O_M-7.5_ model is shown in the context of the D_L-5.7_ model. Arrows indicate the relative reduction in proximity between the β1-β2 linker and α5-β10 loop in D_L-5.7_. Inset in each panel: The spatial relationship between the colored hASIC1a and MitTx subunits is represented using a simplified surface view from the extracellular viewpoint. **d**. Local resolution estimation (left), orientation distribution (middle), and Fourier shell correlation (right) for the map of hASIC1a-T26V at pH 8.5. The resolution at the FSC = 0.143 cutoff (black line) is indicated.

To contextualize O_L_ as the sole conformation observed at pH 6.5—a pH window putatively consistent with a relevant conducting state (Extended Data Fig. 1)—we resolved the structure of hASIC1a in complex with the heteromeric snake venom toxin MitTx, which activates ASIC1 channels independently of pH^7,10^. A single conformation of MitTx–hASIC1a (O_M_) was identified at pH 7.5 and 6.5 (Fig. 2a,b and Extended Data Fig. 4a,b). O_M_ bears strong overall resemblance to O_L_ (C_α_ positional root-mean-square deviation (r.m.s.d.) for O_L-6.5_ versus O_M-7.5_ 0.978 Å). Its TMD configuration is unlike that of MitTx–cASIC1 (ref. ^10^), which exhibits TM helical positionings similar to D_RDS_. The difference may be species specific or relate to experimental conditions. For example, whereas MitTx was added to cASIC1 at pH 7.4 in Tris^10,19^ (regarded as a nonactivating pH condition), it was added to hASIC1a at either pH 7.5 or 6.5 in phosphate buffer, where a partial or full transition to C_L_ or O_L_ before MitTx addition would be expected (Fig. 1).

MitTx has a greater activating effect in phosphate buffer relative to ND96 (Fig. 2c,d and Supplementary Table 1). To evaluate MitTx addition at low pH, channels were first exposed to pH 6.5, prompting activation followed by desensitization. Subsequent addition of MitTx produced a sustained inward current, indicating that MitTx—unlike protons—can transition hASIC1a from a desensitized state to a stabilized conducting state (Fig. 2e,f and Supplementary Table 1).

MitTx forms a ‘church-key’ interaction with hASIC1a, inserting bulky or charged side chains into the lower thumb, palm and wrist domains to communicate outward leverage^10^. Each toxin interacts across the interface between two hASIC1a protomers, in the clockwise and counterclockwise positions relative to the extracellular viewpoint. The side chain of MitTx-α-F14 packs into a pocket formed by the lower thumb domain of the clockwise hASIC1a subunit and the β1–β2 linker of the counterclockwise hASIC1a subunit (Extended Data Fig. 4c); the β1–β2 linker itself interacts with the β11–β12 clutch^13^. This conformation of β11–β12 and β1–β2 MitTx-α-F14 disrupts the pocket where MitTx-α-F14 interacts (Extended Data Fig. 4c). Considering the functional effects of MitTx binding, this specific MitTx–ASIC1a interaction may serve to provide favorable contacts within the nondesensitized β1–β2/β11–β12 region, thereby energetically favoring the open state.

### Substitution of residues in the pre-TM1 region precludes TM2 domain swapping at high pH

**Fig. 3:**
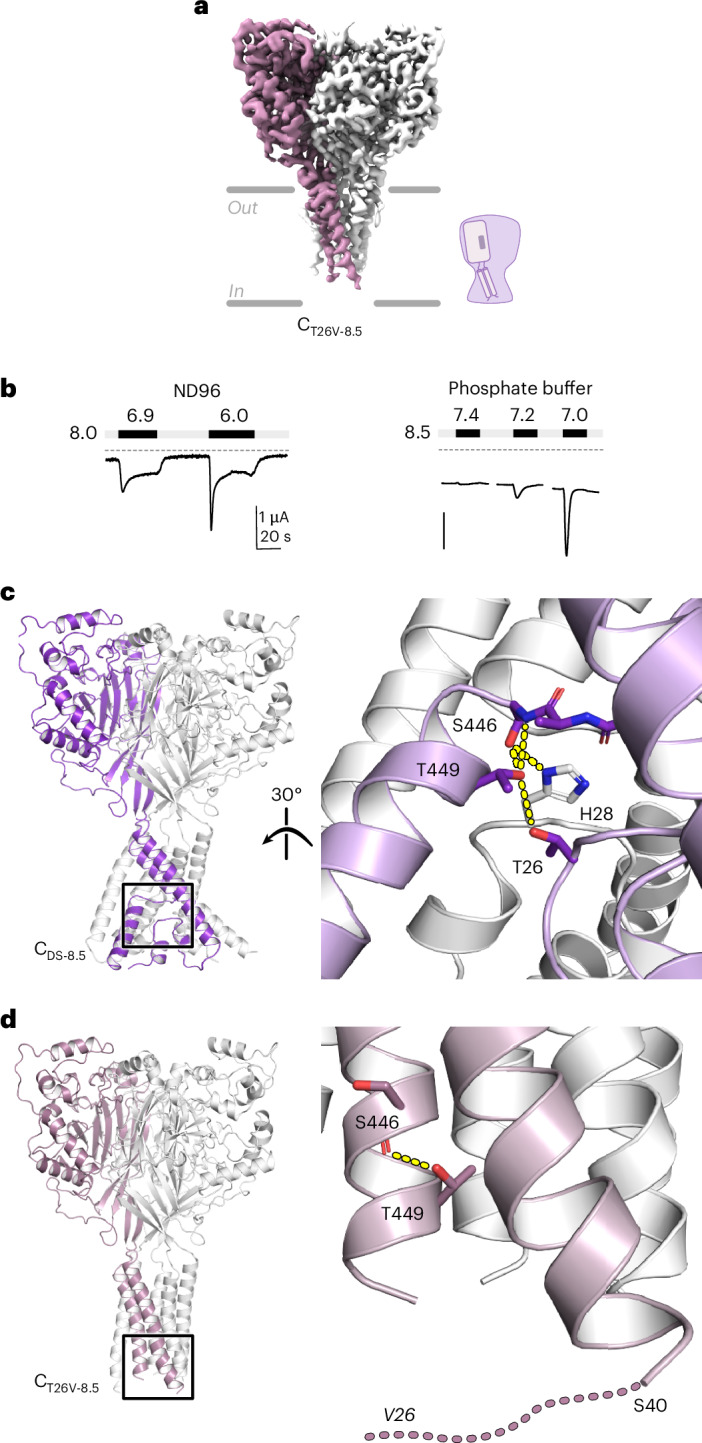
Functional and structural consequences of T26V substitution. **a**, Map of C_T26V-8.5_. Map regions corresponding to an individual hASIC1a subunit are colored. The orientation and approximate position of the homotrimer within the plasma membrane are indicated with gray lines. A cartoon schematic is shown beside the map. **b**, TEVC trace showing activation peaks of hASIC1a-T26V in ND96 (left) and phosphate buffer (right). Vertical scale bars are 1 μA; traces are on the same timescale. The gray dashed line indicates 0 μΑ. In phosphate buffer, oocytes showed notable leak, even at pH 8.5. **c**, The putative hydrogen-bonding network bridging T26, T449 and S446 of the GAS motif and H28 of the HG motif from a neighboring subunit is shown in the model of C_DS-8.5_. Side chains of involved residues and the full GAS motif are shown in stick representation. The putative hydrogen-bonding interaction network around T449 is indicated with dashed yellow lines. **d**, The position of T449 in the model of C_T26V-8.5_ is indicated in stick representation along with its putative hydrogen-bonding partner, the main-chain carbonyl oxygen of S446. V26 is within the unmodeled N-terminal region, as represented with a dashed line extending from the first modeled residue (S40) (Extended Data Fig. 4).

Several substitutions of residues in the pre-TM1 region have been associated with perturbations in gating^27–29^ and divalent ion permeability^30^. To further contextualize the opening of hASIC1a, we structurally characterized one such substitution (T26V) at pH 8.5 (Fig. 3a and Extended Data Fig. 4d). This substitution was chosen primarily for its ability to increase apparent proton sensitivity in mouse ASIC1a, despite its accompanying reduction in ion selectivity^28^.

*Xenopus* oocytes expressing hASIC1a-T26V exhibited a notable leak in phosphate buffer, which appeared largely pH independent between pH 8.5 and 7.4 (Fig. 3b). Although activation was observed at pH 7.2, reliable characterization of activation was hindered by the noticeable leak and pronounced tachyphylaxis. However, the phenotypic perturbations suggest that hASIC1a-T26V more readily transitions into an open state, possibly exacerbated by phosphate buffer.

The single conformation observed at pH 8.5—named C_T26V_—most closely resembles C_A_, except that TM2 can be modeled unambiguously in a linearized conformation. We do not categorize C_T26V_ and C_A_ as the same conformation because of the importance of TM2b to this interpretation; however, the ECD, TM1 and TM2a of C_T26V_ closely resemble those of C_A_ (C_α_ positional r.m.s.d. for C_A-7.5_ versus C_T26V_: 0.660 Å). Their close similarity suggests that the T26V substitution stabilizes the C_A_ conformation of wild-type protein.

The loss of the pre-TM1 reentrant loop at pH 8.5 appears to be a direct consequence of the isosteric T26V substitution, which removes hydrogen-bonding capability. In support of this assignment, the T26S^28^ (but not the T26C)^28,29^ mutant retains wild-type-like function. Thus, the loss of this single hydrogen-bonding interaction may be sufficiently destabilizing to the reentrant loop configuration of pre-TM1 to induce a conformational shift to C_T26V_.

In C_DS_, T26 forms a hydrogen bond with T449 as part of a larger hydrogen-bonding network that connects two highly conserved elements: S446 of the GAS motif and H28 of the HG motif (the latter originating from a neighboring protomer) (Fig. 3c). Therefore, conformational destabilization of the T449 hydroxyl by removal of the T26 hydroxyl may communicate to these functionally crucial residues of hASIC1a. In C_T26V_, the T449 side chain does not maintain any of its C_DS_-specific hydrogen-bonding interactions, although the side-chain hydroxyl retains proximity to the main-chain carbonyl of S446 (Fig. 3d). Because pre-TM1 cannot be modeled in any of the hASIC1a conformations with continuous TM2 helices, there may be further structural consequences of the T26V substitution beyond the observed conformational shift at pH 8.5. For example, T26 in C_A_, O_L_ or D_L_ may engage in other specific interactions that are also disrupted by the T26V substitution, contributing to the observed functional perturbations such as the much slower and incomplete desensitization in mouse^28^ and human (Fig. 3b) ASIC1a.

### Modeled pore characteristics support assignments of functional states

**Fig. 4:**
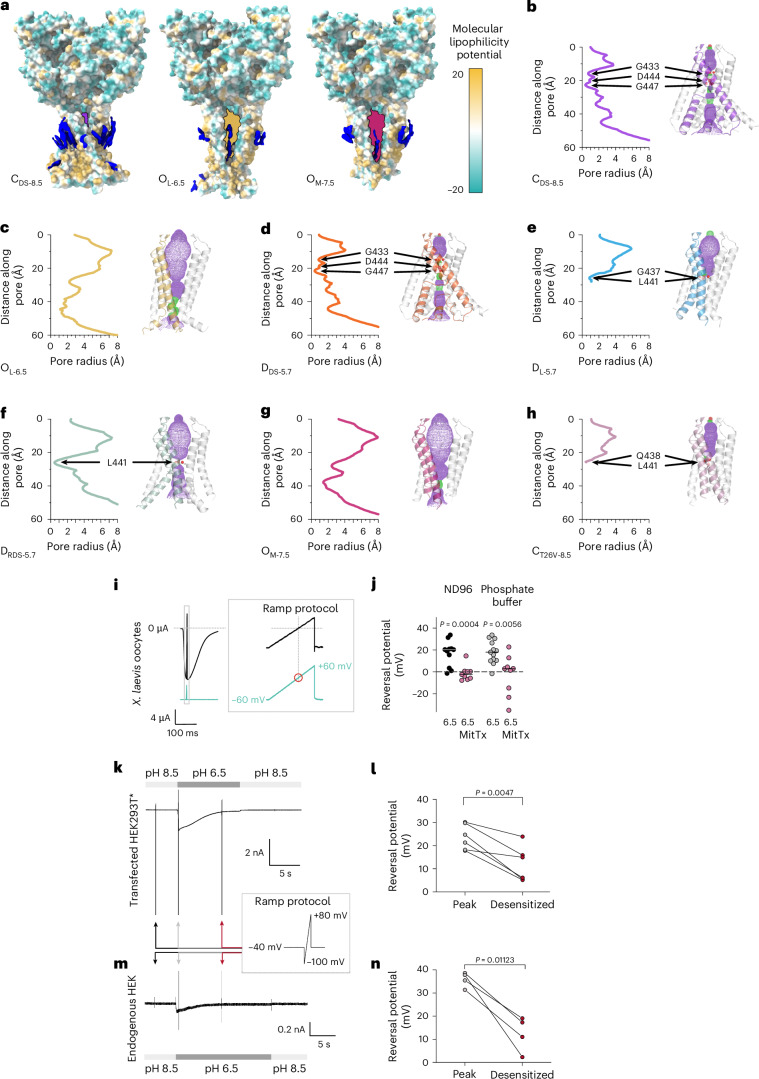
Pore accessibility of hASIC1a conformations and changing selectivity. **a**, Models of C_DS-8.5_ (left), O_L-6.5_ (center) and O_M-7.5_ (right) are depicted in surface view colored by molecular lipophilicity potential. Map features in the TMD region that appear to correspond to neither micelle nor protein are shown in blue. Spaces between TM1 and TM2 of adjacent subunits (fenestrations) are drawn in colored shapes. For O_M-7.5_, the surface corresponding to MitTx is omitted for clarity. **b**–**h**, Pore profile analyses of the indicated model were performed using HOLE. Pore radius plots (left) and pore maps (right) are shown starting from the consensus C_α_ position of V74 at the base of the palm domain. For calculation of the pore profiles depicted in **e**,**h**, the starting point was set to the centroidal position of L441 to approximate the trajectory of an ion that traveled down the pore, encountered the impassable constriction point and stopped; otherwise, the starting point was set to coordinates at the approximate extracellular plasma membrane interface. Pore maps are colored according to radius: red < 1.15 Å < green < 2.3 Å < purple. **i**, TEVC trace showing how the reversal potential was established by running a voltage-ramp protocol from −60 to +60 mV within 200 ms near the peak of a pH 6.5 activation. **j**, Comparison of reversal potentials when channels were activated using pH 6.5 or at the plateau of the response to 20 nM MitTx in pH 6.5 in ND96 (left) or phosphate buffer (right). Sample sizes were as follows: ND96 pH 6.5, *n* = 11; ND96 pH 6.5 + MitTx, *n* = 9; phosphate buffer pH 6.5, *n* = 13; phosphate buffer pH 6.5 + MitTx, *n* = 9. **k**, Whole-cell currents from hASIC1a-transfected HEK293T* cells recorded while applying a 50-ms voltage ramp ranging from −100 to 80 mV (inset) at baseline, during peak current and for the partially desensitized state. **l**, Comparison of reversal potentials measured at peak current (gray dots) or during the partially desensitized state (red dots) (*n* = 6 cells). **m**, Whole-cell endogenous HEK293 ASIC currents recorded while applying a 50-ms voltage ramp from −100 to +80 mV (inset in **k**) at baseline, during peak current and during the (partially) desensitized state. **n**, Comparison of reversal potentials measured at peak current (gray dots) and during the partially desensitized state (red dots) (*n* = 4 cells). Note the different current size in **k**,**m**. Data shown in **l**,**n** are corrected for baseline, leak and LJP. Data are the mean ± s.d.; each data point represents a biological replicate. Statistical analysis was conducted using a two-sided nonparametric *t*-test (Mann–Whitney) (Extended Data Figs. 5 and 6. Source data

**Extended Data Fig. 5:**
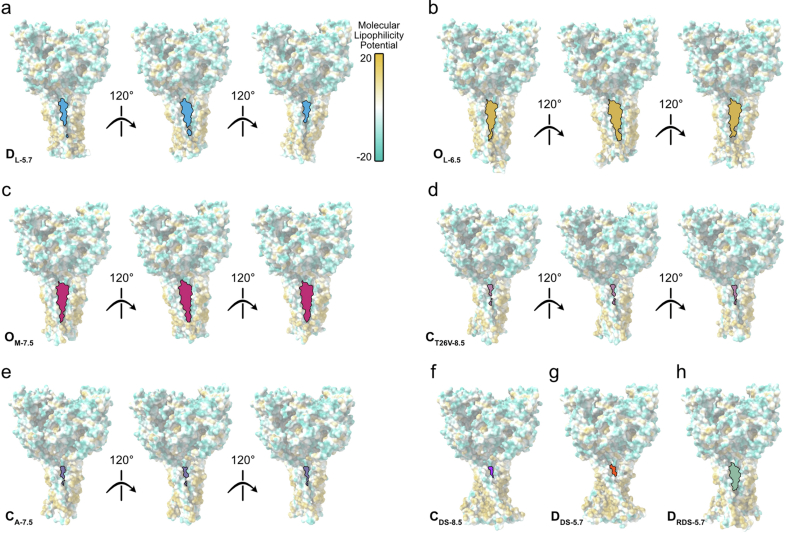
Fenestrations of hASIC1a models. **a-e**. Indicated models are depicted in surface view colored by molecular lipophilicity potential. Spaces between TM1 and TM2 of adjacent subunits (fenestrations) are drawn in colored shapes. For O_M-7.5_, surface corresponding to MitTx is omitted for clarity. Three rotational views are shown for each model. **f-h**. Models and fenestrations are depicted as in a-e. As the maps for these models were calculated with C3 symmetry imposed, only a single orientation is depicted.

**Extended Data Fig. 6:**
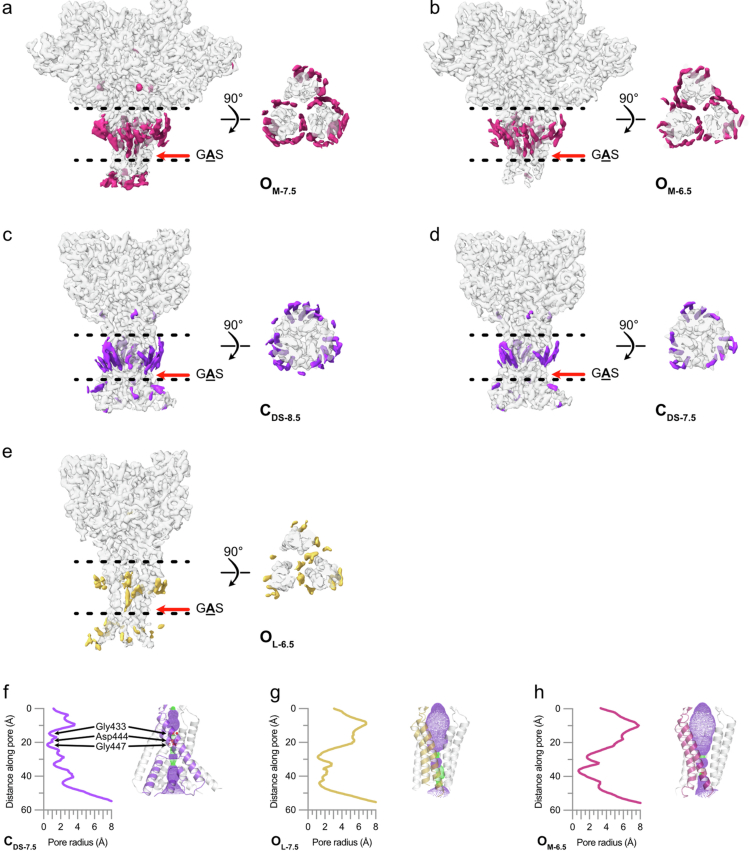
Distribution of unmodeled features in the hASIC transmembrane region and pore profiles. **a-e**. Indicated maps are depicted in white (regions corresponding to modeled protein) or colors (unmodeled map features). Small noncontiguous unmodeled features (‘dust’) are hidden for clarity. The red arrow marks the approximate height of the plane containing Ala445 of the GAS belt. A top-down view of the cross-sectional slab between the two dashed lines (right) shows the extent to which the unmodeled map features contribute to the pore-lining environment in the upper pore region. **f-h**. Pore profile analyses of the indicated model were performed using HOLE. Pore radius plots (left) and pore maps (right) are shown from the consensus C_α_ position of Val74 at the base of the palm domain. For calculation of pore profiles, the starting point was set to coordinates at the approximate extracellular plasma membrane interface. Pore maps are colored according to radius: red < 1.15 Å < green < 2.3 Å < purple. Source data

Although well-documented structural characteristics of the ECD and functional analyses were used to assign putative functional states to the structures, we further analyzed the molecular models to interpret the likely ability of sodium ions to access and travel through the pore. Restricted access to the pore or constrictions within the pore would be consistent with nonconducting channels. Fenestrations between the ECD-proximal ends of each TM1 and its neighboring subunit’s TM2a are a proposed access site because of lack of evidence for the release of constriction points along the three-fold axis of the ECD. These windows extend from the bottom of the ECD deep into the TM region. The widest fenestrations are found in O_L_, D_RDS_ and O_M_. They are slightly asymmetrically narrowed in D_L_ and sharply narrowed in C_DS_, C_A_, D_DS_ and C_T26V_ (Extended Data Fig. 5). Ordered lipid-like features, which wrap the ECD-proximal TMD in maps with both linear and domain-swapped TMDs, also occupy the membrane-embedded portions of the fenestrations in O_L_ and O_M_ (Fig. 4a and Extended Data Fig. 6a–e), suggesting a conformation-dependent intrusion of lipids into the pore-lining region. These features extend to the level of the selectivity filter, possibly forming interactions with the conserved G444 and A445 of the GAS motif.

Structural determinants of channel conductance were analyzed by calculating pore profiles with HOLE^31^ (Fig. 4b–h and Extended Data Fig. 6f–h). Although the helical turns were generally clear enough to define pore-lining residues with reasonable confidence, the specific orientations of many side chains could not be determined. Additionally, unmodeled putative lipids did not contribute to the pore profile calculations. However, the general trends in pore profile can be interpreted.

The pore is lined entirely by TM2 residues, except when reentrant loop pre-TM1 residues line the lower ion permeation pathway. C_DS_ and D_DS_ have the tightest constrictions around TM2a residues D434 and G437 (Fig. 4b,d and Extended Data Fig. 6f). Combined with the narrow fenestrations, the pore features are generally consistent with the assignments of their cASIC1 equivalents as a high-pH closed resting state^12,21,25,26^ and a nonconducting desensitized state^12,22^, respectively.

In other conformations, L441 appears to be the major site of constriction. Despite relatively wide fenestrations, D_L_ and D_RDS_ have very tight constrictions around L441 (Fig. 4e,f); C_T26V_ combines narrow fenestrations and a tight constriction around L441 (Fig. 4h). Because of insufficient map quality within the TM region, C_A_ was omitted from pore profile analysis. However, the overall agreement between C_A_ and C_T26V_ presents a likelihood that C_A_ is similarly constricted around L441. L441 has previously been identified as a functionally important site in ASIC1; alanine substitutions at this position are associated with gating perturbations including reduced ion selectivity^17,32^ and we infer that D_L_, D_RDS,_ C_T26V_ and C_A_ represent nonconducting conformations.

O_L_ and O_M_ are, therefore, the only conformations with wide fenestrations and relatively unconstricted pores (Fig. 4c,g and Extended Data Fig. 6g,h). The combination of widened lateral fenestrations containing putative lipid tails (Fig. 4a and Extended Data Fig. 6a,b,e) and open pores parallels findings from Bandarupalli et al.^33^. In that study, molecular dynamics simulations showed that lipid tails can enter the ASIC3 pore through lateral fenestrations, but only when the channel adopts an open conformation. This state-dependent accessibility supports our interpretation that the conformations captured in the O_L_ and O_M_ datasets reflect an open state, one that permits lipid access from the membrane to the pore. Although both conformations exhibit pore narrowing in the TM2b region, the constrictions are less dramatic than in other conformations. These constricted areas are asymmetric (especially in O_L_) and variable between their representative maps. We consider the possibility that the distal TMD in O_L_ and O_M_—and, for similar reasons, in the other conformations with continuous TM2 helices—is inherently more flexible than in the conformations with domain-swapped TM2 helices and reentrant loop pre-TM1. This flexibility may also increase the susceptibility of this region to distortions because of the biochemical environment of the detergent-extracted protein, potentially inducing an apparent narrowing of the pore.

Although the O_M_ and O_L_ pore profiles are only slightly different (Fig. 4c,g and Extended Data Fig. 5j,k), functional experiments with MitTx-activated hASIC1a suggest a loss of sodium ion selectivity during peak activation (Fig. 4i,j), similar to the reduced selectivity observed in MitTx-activated cASIC1a (ref. ^10^). This phenomenon suggests perturbation of the selectivity filter, although local resolution limitations in the TMD region (Extended Data Figs. 2d,e and 4a,b) preclude unambiguous modeling of key pore-lining residues and complicate the assignments of O_L_ and O_M_ to individual selectivity conditions. However, patch-clamp recordings of ASIC1a-transfected HEK cells reveal a clear shift in reversal potential between the peak and sustained phases of proton activation, indicating reduced sodium selectivity during the later, partially desensitized current (Fig. 4k,l). To test whether this observed shift in reversal potential is because of ion redistribution, as shown for overexpressed P2X receptors^34^, or instead reflects a true, state-dependent change in selectivity, we conducted patch-clamp recordings of endogenous ASIC currents from HEK cells^35^. As expected, endogenous ASIC currents in HEK cells show slightly increased selectivity; however, the recordings from these small endogenous ASIC currents confirm a change in ion selectivity, rather than ion distribution^30,35^ (Fig. 4m,n). This behavior could be consistent with a rearrangement of the selectivity filter during activation or with channels entering multiple conducting states with different desensitization rates. The shared reduction in selectivity during MitTx activation and during the later phase of proton-gated currents could suggest a common structural basis. Because both the MitTx-bound and toxin-free hASIC1a structures adopt a linear pore architecture, this conformation may correspond to the state occupied during the partially desensitized, less-selective phase of proton activation.

**Fig. 5:**
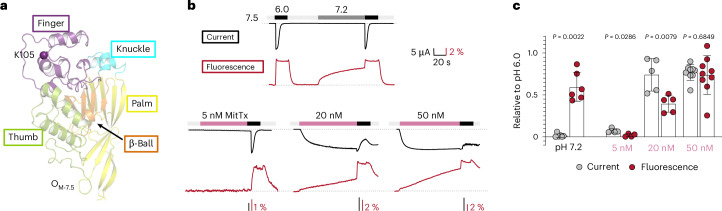
MitTx-induced activation is distinct from proton-induced activation. **a**, Structure showing position K105, where an introduced cysteine was used for fluorescent labeling. **b**, VCF traces of hASIC1a K105C, labeled with Alexa Fluor 488 through a maleimide linker, with currents in black and fluorescence in red. Channels were activated with pH 6.0, conditioned with pH 7.2 and exposed to different concentrations of MitTx at pH 7.5 in phosphate buffer. All traces are on the same timescale and vertical scale bars are 5 μA. **c**, Normalized current and fluorescence responses from **b**. Responses were normalized to the current or fluorescence response evoked by pH 6.0. Four conditions were tested: pH 7.2, 5 nM MitTx, 20 nM MitTx and 50 nM MitTx. Within each condition, normalized current and fluorescence responses were compared. The numbers of oocytes for current and fluorescence measurements, respectively, were as follows: pH 7.2, *n* = 6 and 6; 5 nM MitTx, *n* = 4 and 4; 20 nM MitTx, *n* = 5 and 5; 50 nM MitTx, *n* = 9 and 9. Data are the mean ± s.d.; each data point represents a biological replicate. Statistical analysis was conducted using two-sided nonparametric *t*-test (Mann–Whitney). Source data

However, the ability of MitTx to induce a conducting state also from a fully desensitized state of hASIC1a supports the notion that MitTx-induced activation mechanism might be distinct from that of proton-induced activation. To test this notion directly, we turned to voltage-clamp fluorometry (VCF), which allowed us to monitor conformational rearrangements around the K105 reporter site in response to activation by MitTx or protons^36–38^ (Fig. 5a). While even subactivating pH conditions lead to changes in fluorescence in this position and proton activation results in rapid and maximal change in fluorescence, activation of the channels by MitTx leads to smaller and slower-developing changes in fluorescence (Fig. 5b,c and Supplementary Table 1). These data indicate that the mechanism of activation by MitTx is different to that of protons, even if the final open state bears notable resemblance.

By destabilizing the reentrant loop configuration of pre-TM1, the T26V substitution may increase TMD flexibility at high pH, as proposed for O_L_ and O_M_. Although the constricted pore profile and narrow fenestrations of the C_T26V_ model (Fig. 4h and Extended Data Fig. 5d) are not consistent with C_T26V_ representing an open state, increased TMD flexibility relative to C_DS_ would likely increase its propensity to adopt an open conducting state. The openings at high pH may be transient and, therefore, not identified by structural characterization but may nevertheless explain the leak currents observed with T26V hASIC1a in phosphate buffer. By this interpretation, C_T26V_ represents a destabilized closed state resembling a preopen state with a low barrier to transitioning into a conducting state.

### Unexpected conformational diversity in desensitized states

**Fig. 6:**
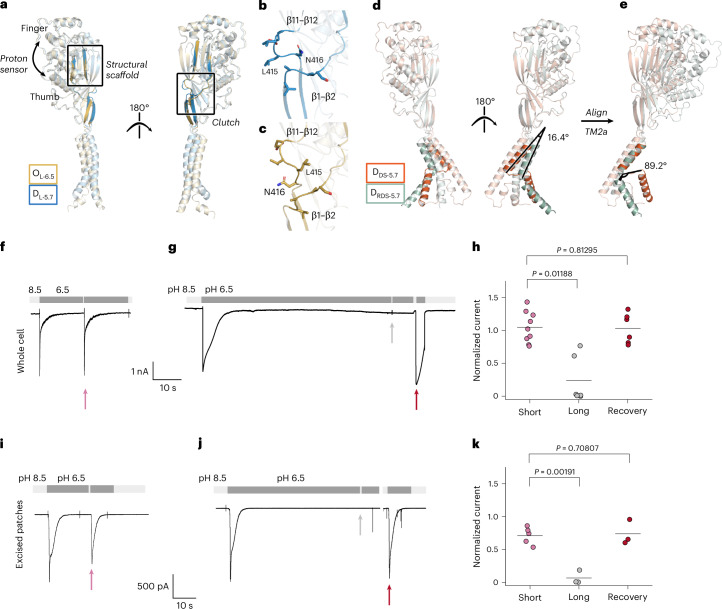
Three desensitized conformations of hASIC1a. **a**, Superposition of a single subunit from D_L-5.7_ and O_L-6.5_. ECD structural elements are indicated over models. The desensitization-associated ‘clutch’ (right) is highlighted in relation to the structural scaffold of the upper palm domain and the proton-responsive thumb and finger domains. **b**,**c**, Close-up views of the clutch region of D_L-5.7_ (**b**) and O_L-6.5_ (**c**). **d**, Relative to D_DS-5.7_, the rotational displacement of TM2a from D_RDS-5.7_ is noted. **e**, TM2a of D_RDS-5.7_ was aligned to TM2a of D_DS-5.7_ to isolate and measure the rotational displacement of the GAS motif. **f**–**k**, The pH-dependent activation of hASIC1a-transfected HEK293T* cells was probed in whole-cell (**f**–**h**) and outside-out excised patch (**i**–**k**) configuration using a short (**f**,**i**) and a long (**g**,**j**) perfusion protocol. Note that outside-out excised patches were used to rule out artifacts arising from slower solution exchange in whole-cell configuration. In the short protocol, pH 6.5 was applied for 12–14 s, followed by a 30-ms (**f**) or 60-ms (**i**) step into pH 8.5 before applying pH 6.5 again. In the long protocol, pH 6.5 was applied for 70–80 s, followed by a 30-ms (**g**) or 60-ms (**j**) step into pH 8.5 before applying pH 6.5 again. To demonstrate cell viability after the long protocol, cells were returned to pH 8.5 for 5–10 s before a third application of pH 6.5 (recovery). Arrows at the positions of second and third activations match the color coding used in **h**,**k**. In **h**,**k**, currents from **f** (*n* = 9 cells) or **i** (*n* = 5 patches) and **g** (*n* = 6 cells) or **j** (*n* = 3 patches) are normalized to the first activation peak. Statistical analysis for **h**,**k** was conducted using a two-sided nonparametric *t*-test (Mann–Whitney) (Extended Data Fig. 7).

**Extended Data Fig. 7:**
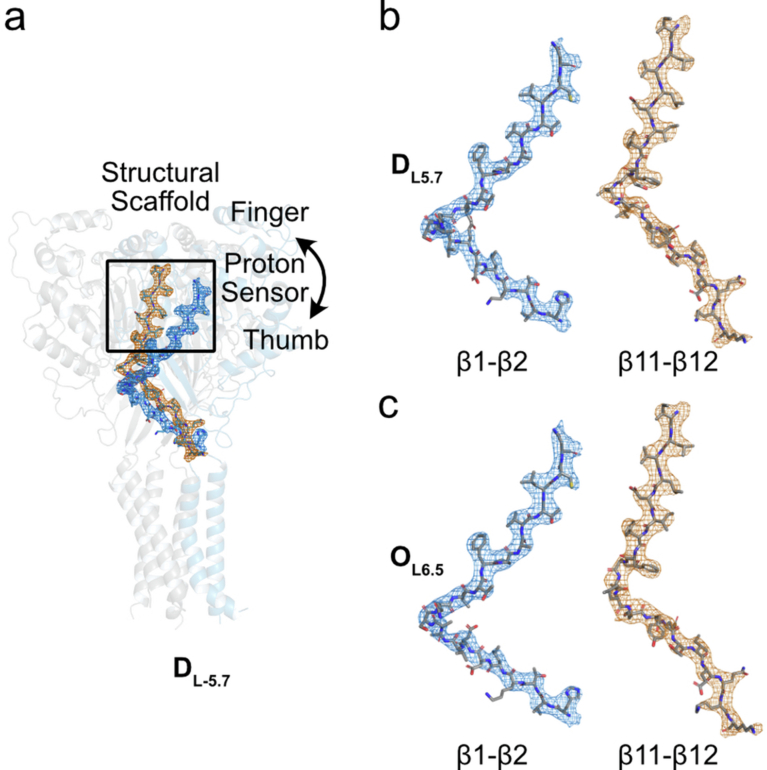
Map quality of hASIC1a in the β1/β2 and β11/β12 regions. **a**. The positions of the β1, β2, β11, and β12 sheets of D_L-5.7_ are shown in stick representation with map mesh overlay. Proximal structural motifs are labeled. **b**. The β1-β2 and β11-β12 sheets and linkers of D_L-5.7_ are shown in stick representation with map mesh overlay. **c**. The β1-β2 and β11-β12 sheets and linkers of O_L-6.5_ are shown in stick representation with map mesh overlay.

ASIC1a function normally involves rapid and complete desensitization after proton-induced activation^39^. A key feature of desensitization involves the β11–β12 and β1–β2 linkers^40,41^, which rearrange at low pH (Fig. 6a–c and Extended Data Fig. 7). At pH 5.7, all ECDs present the collapsed acidic pocket and disengaged clutch associated with the desensitized state but the TMs feature remarkable conformational diversity (Fig. 6a,d,e).

The ability of hASIC1a to form multiple kinetically distinct desensitized states has been previously suggested^42^. To explore whether there is direct transition between kinetically distinct states consistent with structural rearrangement, we performed whole-cell patch-clamp electrophysiology experiments in which we varied the duration of channel exposure to low pH before briefly returning to high pH, followed by a second application of low pH. Following relatively short initial acidification (12–14 s) and a 30-ms recovery step in pH 8.5, reapplication of low pH evoked a current amplitude comparable to the control response (Fig. 6f). In contrast, prolonged initial acid exposure (70–80 s) abolished subsequent currents, indicating entry into a desensitized state that was not readily reversed by brief alkalinization (Fig. 6g). To ensure that the loss of current did not reflect channel rundown, we added an extended second recovery period (5–10 s) at high pH before subsequent acid challenge. Under these conditions, current responses reappeared, confirming that the channels remained functional and could recover given sufficient time at alkaline pH (Fig. 6h). To exclude perfusion artifacts and to ensure that the 30-ms recovery step was not limiting, we conducted similar experiments (with a 60-ms recovery step in pH 8.5) in outside-out excised patches (Fig. 6i–k) and obtained similar results. These findings support the existence of at least two distinct desensitized conformations of hASIC1a with different rates of recovery.

Although it is unclear which conformational transition may cause this effect, the rotational differences between D_RDS_ and D_DS_ are best described by the transitions that would be necessary to move between them. The conformational rearrangement required to transition from D_RDS_ to D_DS_ involves a clockwise twisting (relative to the intracellular view) of both TM1 and TM2. The rotational displacement of TM2 between D_RDS_ and D_DS_ involves a 16.4° upward swing of TM2a and an 89.2° rotation of the GAS motif and TM2b within the plane of the membrane (Fig. 6d,e).

Structural characterizations of cASIC1 with disengaged clutch have previously described either domain-swapped^10,12,22,43^ or continuous^11,19,20^ TM2 helices; D_DS_ bears resemblance to the former and D_L_ to the latter in their respective TMD regions. Although there is no direct cASIC1 analog to D_RDS_ in the library of solved structures, the crystal structure of MitTx-bound cASIC1 (ref. ^10^) shares the otherwise unique TMD rotation. However, MitTx–cASIC1 has a nondesensitized ECD and slightly different TM helical positions, including a lateral displacement of TM2a that alleviates the constriction site observed in D_RDS_ at L441 (L440 in cASIC1). Together, these findings suggest that desensitization of hASIC1a involves multiple nonconducting TMD conformations, underscoring the striking degree of TMD conformational variability observed across the functional states of hASIC1a.

## Discussion

In this study, we used complementary structural, functional and fluorometric approaches to establish that hASIC1a open conformations are associated with linear TM helix conformations and the TMD can adopt a surprisingly diverse array of conformations in open, closed and desensitized states. We further provide direct evidence for mechanistic differences between proton and toxin activation, as well as for how pre-TM1 forms interactions that stabilize the closed channel. Together, these results establish a framework to understand the structural underpinnings of ASIC gating, which have long remained elusive.

### Implications for ASIC gating

ASIC1 has traditionally been viewed to have at least three discrete functional states: resting, activated and desensitized. In this study, we observed three structurally distinct conformations in the ECD that correspond to these functional states. Under pH 7.5 conditions, we observed a mixture of two distinct ECD conformations within a single sample. This conformational heterogeneity suggests that ASIC1a can adopt multiple ECD conformations in the absence of a strong activating stimulus. Such heterogeneity could explain how state-dependent modulators like PcTx1, which bind selectively and alter SSD, exert their effects^44^. Our structural observations provide a mechanistic basis for how such modulators may act by stabilizing specific preexisting conformations. Given that PcTx1 has served as a foundation for designing new ASIC-targeting compounds^45,46^, our findings may inform future efforts to exploit this intrinsic conformational plasticity for therapeutic development.

**Fig. 7:**
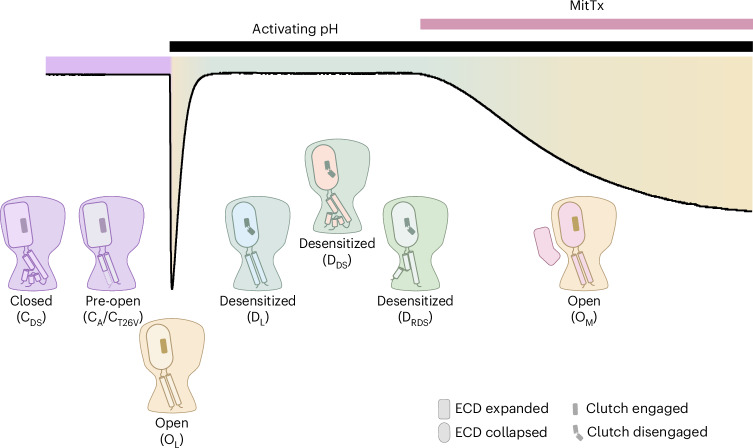
Proposed distribution of distinct hASIC1a conformations along a hASIC1a current trace. At high pH, hASIC1a adopts a nonconducting, resting conformation (C_DS_) and a closed preopen state (C_A_/C_T26V_) with a lower transition barrier to the open state. The channel opens at low pH (O_L_) and rapidly desensitizes into several states (D_RDS_, D_DS_ and D_L_). MitTx can activate hASIC1a (O_M_) from the desensitized (shown) or closed states, through a mechanism distinct from pH-dependent activation.

The three-state model provides a useful paradigm but is too simplistic to account for the range of functional and structural behavior observed^42,47,48^. By varying pH between 5.7 and 8.5, we captured six different conformations with four distinct TMD architectures: two with a twisted TMD, domain-swapped TM2b helices and reentrant loop pre-TM1 domains in resting and desensitized conformations (C_DS_ and D_DS_), two with continuous TM2b helices in an untwisted arrangement, in conducting and desensitized conformations (O_L_ and D_L_), one desensitized conformation with untwisted TMD and domain-swapped TM2bs (D_RDS_) and one high-pH conformation with untwisted TMD but unknown TM2b architecture (C_A_). Additional conformations resembling C_A_ and O_L_ were captured through probing the modulatory effects of a gating-related substitution (C_T26V_) and agonist toxin MitTx (O_M_); the T26V substitution and MitTx binding are functionally distinct but both are associated with increased channel activity, decreased ion selectivity^28^ and reduced desensitization rates. Taking together the 11 structural models representing six major conformations of hASIC1a, we propose an overall mechanistic model of conformational rearrangements upon activation (pH-induced or toxin-induced) and desensitization (Fig. 7).

Domain-swapped TMD conformations were observed at both high and low pH but neither at pH 6.5 nor in the presence of MitTx, the two experimental conditions intended to probe the TMD conformation associated with channel conduction. This result recalls a previous study that links structural features of the linearized TM2 conformation to a functional open state. Cysteine accessibility experiments^29^ provide support for an activation mechanism that displaces the reentrant loop, as seen with the linearized TM2 conformations. R43 has both conformation-dependent and state-dependent exposure to the pore environment, becoming chemically accessible from the pore-facing side upon channel activation and becoming unshielded by the reentrant loop between domain-swapped and linear conformations.

Another striking result of the structural study was the identification of three desensitized states. While it has proven challenging to assign specific biological roles to each structural state, there have been multiple bodies of evidence supporting the existence of functionally diverse desensitization mechanisms and desensitized states. These mechanistic states include acute desensitization after channel activation, which may itself include multiple subpopulations with different desensitization kinetics^9^ (Extended Data Fig. 1e). This complexity could be explained by structural transitions from multiple open states or into multiple desensitized states. Additionally, we demonstrated that prolonged incubation of hASIC1a at activating pH in phosphate buffer increases the necessary recovery time from desensitization (Fig. 6f–k). This is consistent with an initial acute desensitization conformation (characterized by swift recovery upon return to resting pH) undergoing a gradual transition into a desensitized conformation with slower recovery kinetics, similar to previous work by others^42^.

Two other desensitization mechanisms are well characterized for hASIC1a. One long-lived desensitized state accumulates through repeated stimulation and results in an incremental reduction in response (tachyphylaxis)^40,42,47–49^. Such persistent desensitized states are not unique to ASICs and have also been described for other ligand-gated ion channels, including glutamate receptors^50^. Another proposed desensitization mechanism is induced directly from the closed resting state by prolonged incubation at subactivating pH (SSD)^51^. A recent D_DS_-like cASIC1 structure was proposed to reflect the SSD-associated conformation, as it was solved at a putative subactivating pH (pH 7)^12^; the identification of D_DS_ at pH 5.7 may reflect an alternative pathway to an equivalent structural state, although we lack sufficient evidence to confidently assign any of the three identified desensitized conformations of hASIC1a to one or more functional roles.

The conformation of the GAS motif differs markedly between the domain-swapped and linear architectures, with potential implications for ion selectivity. In the domain-swapped conformations, this selectivity filter region adopts a belt-like geometry, disrupted by a conserved glycine that introduces a helical break. In contrast, in the linear conformation, this region forms a continuous α-helix. Both proton-induced and MitTx-induced activation have been associated in this study with TM2 linearization, as well as the T26V substitution shifting the resting-state equilibrium toward the linearized conformation; reduced ion selectivity is also associated with MitTx addition (Fig. 4i,j) and pre-TM1 substitutions including T26V^28^. Substitutions such as T26V could influence selectivity upon channel opening by altering the GAS motif configuration or decreasing the dwell time of TM1 and reentrant loop residues within the conduction pathway. Although our structural studies have not revealed an alternative activated state exposing these regions to the pore, we described a functional transition from higher-selectivity to lower-selectivity conducting states during a single proton activation event. One explanation for these various structural and functional observations is that O_L_ and O_M_ both represent the low-selectivity open conformation, with C_T26V_ representing a nonconducting state that is biased toward accessing only this open conformation.

Considering the dual roles of the ASIC1 GAS motif in domain swapping and governing ion selectivity, we note that the highly selective epithelial Na^+^ channel (ENaC) does not fully conserve the motif. In particular, the γ subunit substitutes a serine for the TM2-breaking glycine, although it is not known whether γ-containing ENaCs can form domain-swapped TM2 helices. To date, there has been no structural characterization of activated ENaC to provide detailed insights into the structural basis of its enhanced ion selectivity. However, these observations demonstrate the structural flexibility of the ASIC1a selectivity filter and suggest that distinct conformations may contribute to differences in ion discrimination across ENaC/degenerin family members^28^, highlighting the need for further studies to clarify the functional consequences of these structural rearrangements.

### Potential methodological constraints

The activation curve suggests that the majority of channels will access the open state at pH 6.5 (Extended Data Fig. 1b,f); however, current recordings show rapid and complete desensitization and desensitized conformations were captured only at pH 5.7. This discrepancy may arise from differences between the buffers used for recording and grid preparation. Alternatively, detergent extraction of hASIC1a may stabilize a particular conformation that is less persistent in the cell membrane.

The use of digitonin may influence hASIC1a’s proton-mediated conformational transitions and the use of phosphate buffer has functional consequences such as shifts in the activation and SSD curves (Extended Data Fig. 1). This also means that the observed changes in ion selectivity or recovery from desensitization may differ in more conventional buffers. However, this study has reproduced equivalents of cASIC1a structures solved in a membrane lipid environment^12^ at three of the four sampled pH conditions (C_DS-8.5_, C_DS-7.5_ and D_DS-5.7_), validating these reagents for stabilization of hASIC1a. Moreover, our structural examination of a single hASIC1a construct using a consistent buffering system captured most of the conformations previously seen in cASIC1 and others not previously observed in cASIC1 (Table 1), supporting this methodology when interrogating the hASIC1a conformational space. Future experiments using technologies that preserve a more native membrane environment might further elucidate ASIC gating mechanisms.

We also note that local resolution in the TM region imposes limitations on the accuracy of pore radius calculations (Extended Data Figs. 2d,e and 4a,b). While the backbone trace and helical register are generally well resolved, side-chain orientations remain ambiguous in several regions, especially near the lower pore. The apparent intrusion of lipid-like map features into the pore region (Extended Data Fig. 6a–e) may also alter the effective pore radius and local chemical environment from what the protein models would suggest. As a result, precise interpretation of pore geometry and ion conduction pathways should be approached with caution.

### Outlook

The six major conformations captured in this study provide detailed mechanistic insights into ASIC1a function, highlighting an unexpected intrinsic conformational variability of the TMD during both open and desensitized states of hASIC1a. These conformational rearrangements are mediated by proton or toxin binding to the ECD to produce functionally distinct states, albeit suggesting linear TMD helices as a common denominator for otherwise slightly divergent open states. By comparison, less is known about structural transitions and conformational plasticity in other members of the broader ENaC/degenerin family; however, recent work suggests that there might be important differences in ENaCs^28,52^ and neuropeptide-activated FaNaCs (FMRFamide-activated sodium channels)^53,54^.

Our study focuses on homotrimeric hASIC1a; it is important to consider how these findings might relate to heteromeric ASIC channels, which also exist in the brain. Key elements implicated in gating, such as the conserved HG motif and T26 of pre-TM1 and the GAS motif of TM2, are present across ASIC subtypes, suggesting that similar conformational transitions may underlie gating in heteromeric assemblies such as ASIC1a/1b or ASIC1a/2b. However, heteromeric channels exhibit distinct functional properties, including different proton sensitivities, compared to homomeric ASIC1a. We speculate that the distribution and relative occupancy of conformational states may differ from those captured in this study. Structural and functional characterization of heteromeric ASICs will be critical to determine the extent to which these conformations are conserved or diverge across physiologically relevant assemblies.

## Methods

### Cell lines

*Spodoptera* *frugiperda* Sf9 cells (Thermo Fisher Scientific, Cat# 12659017), used for generating baculovirus, were cultured at 27 °C in suspension in Sf-900 III SFM (Thermo Fisher Scientific). HEK293S GnTI^−^ suspension cells, used for expression of hASIC1a for structural studies, were obtained from the American Type Culture Collection (ATCC; CRL-3022) and cultured at 37 °C and 8% CO_2_ in a humidified environment. HEK293S GnTI^−^ cells were cultured in FreeStyle 293 expression medium (Thermo Fisher Scientific) supplemented with 2% FBS (Thermo Fisher Scientific). HEK293T cells were originally purchased from the ATCC (CRL-3216). The HEK293T cell line, in which endogenous hASIC1a was removed by CRISPR–Cas9 (hereafter HEK293T*), was previously described^37^. HEK293T* cells were grown in monolayer in T75 flasks (Orange Scientific) at 37 °C in a humidified 5% CO_2_ atmosphere. The culture medium was DMEM (Thermo Fisher Scientific) supplemented with 10% FBS (Thermo Fisher Scientific) and 1% penicillin–streptomycin (10,000 U per mL; Thermo Fisher Scientific).

### Expression and purification of hASIC1a

A pEG BacMam vector^55^ encoding a full-length human ASIC1a N-terminally tagged with eight histidines (8×His) and enhanced green fluorescent protein (eGFP), with a thrombin cleavage site between eGFP and hASIC1a (Supplementary Fig. 1a), was synthesized (GenScript) and used to generate baculovirus. HEK293S GnTI^−^ cells were grown in suspension to a density of 3.5 × 10^6^ cells per mL. Cells were infected with baculovirus and incubated for 8–10 h at 37 °C, after which sodium butyrate was added to 10 mM and the cells were transferred to 29 °C. For expression of hASIC1a-T26V (generated by Genscript), 10 μM amiloride was also added to the culture medium at this time. After a total incubation time of no more than 48 h after virus addition, the cells were pelleted and pellets were washed with Tris-buffered saline before another round of centrifugation. The resultant pellet was snap-frozen in liquid nitrogen and stored at −80 °C. Small aliquots of these cells were reserved for preliminary evaluation of GFP-containing protein using fluorescence-detection size-exclusion chromatography (FSEC) (Supplementary Fig. 1b).

The frozen pellet was resuspended in buffer A (20 mM HEPES pH 7.5, 150 mM NaCl, 0.01 mg mL^−1^ deoxyribonuclease I and protease inhibitor cocktail (Pierce)). Cells were lysed by sonication. The lysed cells were centrifuged (8,000*g*, 20 min, 4 °C) and the supernatant was collected. Membranes were isolated by centrifugation (100,000*g*, 1 h, 4 °C). The pelleted membranes were snap-frozen in liquid nitrogen and stored at −80 °C.

The material was resuspended in fresh buffer A and homogenized using a Dounce homogenizer, before adding 1% (w/v) digitonin. Membrane proteins were solubilized for 1 h at 4°C and then debris was removed by centrifugation at 100,000*g* for 1 h at 4 °C. Homemade GFP nanobody (GNB) affinity resin (1 mL of packed resin per g of membrane) was preequilibrated in buffer B (20 mM HEPES pH 7.5, 150 mM NaCl and 0.1% (w/v) digitonin) and transferred to a glass flex-column. Supernatant was then slowly flowed over the column to allow for protein association. The column was washed with five column volumes of buffer B with 2 mM ATP and 2 mM MgCl_2_, then five column volumes of buffer B with nuclease (Pierce) and finally five column volumes of buffer B with 5 mM CaCl_2_. The resin was then incubated in buffer B with 5 mM CaCl_2_ and 30 mg thrombin (Prolytix) per mL of resin at room temperature for 1 h. Cleaved hASIC1a was eluted with three column volumes of buffer B and collected in fractions of one column volume. Peak fractions were concentrated using a centrifugal filter (50-kDa molecular weight cutoff; Millipore) and run on a Superose 6 Increase 10/300 gel filtration column (Cytiva) in gel filtration buffer (50 mM sodium phosphate, 200 mM NaCl, 1 mM TCEP and 0.1% (w/v) digitonin). The pH of the buffer varied between 8.5, 7.5, 6.5 and 5.7 depending on the desired final condition of the protein. Peak fractions were pooled and concentrated to ~4 mg mL^−1^. For certain experiments, MitTx (Alomone Labs) was added at a 1:1 molar ratio (ASIC monomer to toxin). All samples used for grid preparation had fluorinated octyl maltoside (Anatrace) added to a final concentration of 10 μM.

Quantifoil R 2/1 200-mesh Au grids were glow-discharged at 15 mA for 30 s. A Vitrobot Mark IV (Thermo Fisher Scientific) was used for blotting and freezing. In general, a 3-μl drop was applied to the surface and then promptly wicked away using a torn piece of filter paper. A second drop of the same volume was applied before blotting and plunge-freezing in liquid ethane (blot time: 3.5 s, wait time: 0 s, drain time: 0 s, blot force: 1, 100% humidity, 12 °C). Grids were clipped under vapor-phase nitrogen cooled by liquid nitrogen and stored under liquid nitrogen before screening and collection.

Protein quality was assessed using FSEC (Supplementary Fig. 1c) and SDS–PAGE (Supplementary Fig. 1d). The latter technique resolved three distinct bands, one of which was of a size consistent with intact hASIC1a monomer. The other two bands were characterized with N-terminal sequencing and were revealed to be two segments of a cut hASIC1a monomer, most likely a result of off-target cleavage. FSEC traces and cryo-EM maps suggested that this partial cleavage in the primary structure did not impact the tertiary structure of the protein (Supplementary Fig. 1d–g).

### Image acquisition and processing

Grids were screened on a 200-kV Glacios TEM (Thermo Fisher Scientific) with a K3 Detector (Gatan) and promising candidates underwent data collection on a 300-kV Titan Krios (Thermo Fisher Scientific) with a K3 Detector at the Pacific Northwest Center for Cryo-EM (PNCC) or the Howard Hughes Medical Institute (HHMI) Janelia Research Campus.

Following collection, map processing took place using cryoSPARC^56^ following a generalized workflow. In brief, this included preprocessing with patch motion correction and patch contrast transfer function (CTF) fitting followed by blob picking, several rounds of 2D classification and ab initio map generation. This ab initio map was then used to generate templates for extraction of a new particle stack, which was used to generate a new map. This map subsequently underwent multiple rounds of heterogeneous refinement and 3D classification to remove low-quality particles from the stack and to evaluate potential conformational heterogeneity. Successive iterations of local refinement, nonuniform refinement^57^ and global and local CTF refinement resulted in the final map (Supplementary Fig. 2).

For hASIC1a prepared at pH 5.7, multiple structurally unique populations were identified using symmetry expansion before focused 3D classification in the TM and micelle region, followed by removal of duplicate particles from each of the three resultant particle stacks (Supplementary Fig. 2d). For hASIC1a prepared at pH 7.5, ECD-focused 3D classification yielded two distinct populations, one of which was further classified into two structurally unique populations using heterogeneous refinement (Supplementary Fig. 2b). To enhance TMD map quality and supported by apparent lack of symmetry-breaking structural elements, the maps of C_DS-8.5_, C_DS-7.5_, D_DS-5.7_ and D_RDS-5.7_ were processed with *C*_3_ symmetry; all others were processed with *C*_1_ symmetry.

### Model building and refinement

Models were built into cryo-EM maps (Supplementary Fig. 3) using a combination of Coot^58^ and the Isolde plugin for ChimeraX^59,60^, using a cASIC1 model (PDB 6VTL)^12^ and the MitTx from a MitTx–cASIC1 model^10^ (PDB 4NTW) as the starting models. The modeling process consisted of iterative rounds of model building and real-space refinement in PHENIX^61,62^. Validation of final models was performed using MolProbity^63^. Pore profiles were characterized using HOLE^31^. Model-model positional r.m.s.d. was calculated in PyMol (Schrödinger); relative rotational displacements between models were measured using the AngleBetweenHelices plugin for PyMol. Model and map figures were prepared using PyMol and ChimeraX^59^.

### Whole-cell patch-clamp electrophysiology

For patch-clamp experiments HEK293T* cells were seeded in a T25 Flask (Orange Scientific) and incubated for 24 h at 37 °C before the cells were transfected with 2 μg of cDNA coding for hASIC1a (inserted into pcDNA3.1+ vector upstream IRES GFP) using polyethylenimine 25K (Polysciences) in a 1:3 ratio. Cells were used for patch-clamp experiments 24–36 h after transfection. On the day of patch-clamp recordings, the HEK293T* cells expressing hASIC1a were reseeded on glass coverslips to enable lifting of the cell from the coverslip and positioning in front of a perfusion tool to facilitate rapid solution/pH exchange. The custom-built glass perfusion tool equipped with four adjacent barrels was mounted on a piezo head (PZT head, Siskiyou) and controlled by the MXPZT-300 solution exchanger (Siskiyou). The cells were voltage-clamped at −40 mV at room temperature in the whole-cell configuration using an Axopatch 200B amplifier (Molecular Devices). All recordings were performed using the pCLAMP 10 software (Molecular Devices) and an Axon Digidata 1550A digitizer (Molecular Devices) at 10 kHz. Patch pipettes with a resistance between 3 and 5 MΩ were pulled on a pipette-puller (Model P-1000, Sutter instruments) using borosilicate glass capillaries (World Precision Instruments). The physiological extracellular recording solutions contained 150 mM NaCl, 5 mM KCl, 1 mM MgCl_2_, 2 mM CaCl_2_, 10 mM HEPES and 10 mM D-glucose and were adjusted to pH 5.6, 6.9 and 8.0 with NaOH and HCl. The phosphate recording solution contained 90 mM NaCl and 5 mM sodium phosphate. Phosphate buffers of varying pH were created using different ratios of 0.1 M sodium phosphate monobasic and sodium phosphate dibasic solutions to generate a sodium phosphate stock solution at the desired pH. The intracellular solution consisted of 30 mM NaCl, 120 mM KCl, 1 mM MgCl_2_, 0.5 mM CaCl_2_, 5 mM EGTA, 2 mM Na_2_ATP and 10 mM HEPES adjusted to pH 7.4. pCLAMP 10 (Molecular Devices) was used for analyzing current amplitudes and rate constants were fitted with single-exponential functions to the decay of the current. Graphs were generated using GraphPad Prism 10.

For the desensitization protocols, solutions, data acquisition and cells were prepared as described above. No correction was applied for the liquid junction potential (LJP). At the beginning of the recording, a hASIC1a-transfected cell in whole-cell configuration was positioned in front of the perfusion tool at the barrel with pH 8.5 for 5 s. Subsequently, the piezo actuated a shift to the barrel with pH 6.5 for 14 (short protocol) or 70–80 s (long protocol), followed by a return to the pH 8.5 barrel for 30 ms. The barrel was then switched back to pH 6.5 for 12 s and it reverted to pH 8.5. As there was no observable current in some long protocols, the piezo shifted back to pH 6.5 for a short recovery check of the peak current. All data obtained were evaluated using ClampFit 10.7.

ASIC permeability was assessed using a 50-ms voltage ramp from −100 mV to 80 mV at baseline (pH 8.5), during peak current (pH 6.5) and for partial desensitization (pH 6.5). For the solutions above, the reversal potential for sodium was calculated to be 27 mV. Reversal potentials during peak and partially desensitized currents were corrected by subtracting leak, baseline and the LJP (2.161 mV). All data obtained were evaluated using ClampFit 10.7.

### Two-electrode voltage-clamp (TEVC) electrophysiology and VCF

The human ASIC1a cDNA with a C-terminal 1D4 tag was cloned into a pcDNA3.1+ vector, obtained from TWIST bioscience. T26V and K105C substitutions were introduced using custom-made DNA mutagenesis primers (Eurofins Genomics) and site-directed mutagenesis (PfuUltraII Fusion polymerase, Agilent), followed by sequencing the full coding frame (Eurofins Genomics or Macrogen). Mutagenesis primer pairs were CAGCAGCAGCGTGCTGCACGGAC and GCAAAGGCCTGAATGG (forward and reverse; T26V) and CCAGGTGTCCTGTAACGACCTGTATC and GAGAACCGGAACTCG (forward and reverse; K105C). Xba1 was used for linearization of cDNAs before transcription to capped cRNA with the Ambion mMESSAGE mMACHINE T7 kit (Thermo Fisher Scientific). *Χenopus* oocyte preparation and the recording setup were the same as previously described^38^. Recording solutions were either Ca^2+^-free ND96 (96 mM NaCl, 2 mM KCl, 1.8 mM BaCl_2_, 1 mM MgCl_2_ and 5 mM HEPES) pH-adjusted with HCl or NaOH or phosphate buffer (90 mM NaCl and 5 mM sodium phosphate, described above). For experiments with MitTx, 0.05% BSA (≥98% essentially fatty acid free; Sigma-Aldrich) was added to the buffer on the day of use. For SSD experiments, cells were preconditioned for 2 min before activation with pH 6.0. MitTx was applied for 2 min or until a plateau was reached. The reversal potential was established by running a 200-ms voltage ramp from −60 mV to +60 mV through peak activation at pH 6.5 or during maximal activation by 20 nM MitTx at pH 6.5. For VCF experiments, oocytes expressing hASIC1a K105C were labeled for 30 min in ΟR2 solution (82.5 mM NaCl, 2 mM KCl and 1 mM MgCl_2_, pH 7.4) containing 10 μΜ Alexa Fluor 488 C_5_ maleimide (Thermo Fisher Scientific). Oocytes were then washed twice with OR2 solution and stored in the dark until further use. Recordings and data analysis were performed as previously described^38^.

### Statistics and reproducibility

No statistical method was used to predetermine sample size. The number of cryo-EM images and particles was determined by the allotted microscope time, as defined by the facility. The sample size for electrophysiology experiments was based on previous published studies.

Samples for cryo-EM experiments followed the same expression and purification procedures up until the final step, where the pH condition was altered for the test condition. Although there were some variations in protein yield, the SDS–PAGE band patterns remained consistent. For electrophysiology experiments, each trial was repeated and the data points are included in the figures.

No data were excluded from the analyses.

The experiments were not randomized. During cryo-EM data collection, regions of the grids were carefully selected on the basis of ice thickness and particle density, with the goal of achieving high-quality data. However, randomization was used during data analysis in the cryoSPARC software, where particles were randomly split into two groups to calculate overall map resolution. For electrophysiology experiments, randomization was not practically feasible. Control of covariates was not achievable because of the experimental design, where the concentration of DNA (for transfection) or mRNA (for injection) was predetermined.

The investigators were not blinded to allocation during experiments and outcome assessment. Blinding is not feasible for cryo-EM experiments, as sample details must be provided before data collection to ensure that conditions are correctly assigned to the appropriate sample. The same applies for electrophysiology.

### Reporting summary

Further information on research design is available in the Nature Portfolio Reporting Summary linked to this article.

## Online content

Any methods, additional references, Nature Portfolio reporting summaries, source data, extended data, supplementary information, acknowledgements, peer review information; details of author contributions and competing interests; and statements of data and code availability are available at 10.1038/s41594-026-01845-0.

## Supplementary information

Supplementary Information Supplementary Data Figs. 1–3, Tables 1–4 and raw gel image.

Reporting Summary

Peer Review File

## Source data

Source Data Fig. 2 Statistical source data.

Source Data Fig. 4 Statistical source data.

Source Data Fig. 5 Statistical source data.

Source Data Extended Data Fig. 6 Source data used to generate pore radius profiles of the ion channel.

## Data Availability

Cryo-EM maps and atomic models were deposited to the Protein Data Bank and EM Data Bank and are publicly available as of the date of publication through accession codes PDB 9E4A, PDB 9E4B, PDB 9E4C, PDB 9E4D, PDB 9E4E, PDB 9E4F, PDB 9E4G, PDB 9E4H, PDB 9E4I, PDB 9E4J and PDB 9E4K and EMD-47503, EMD-47504, EMD-47505, EMD-47506, EMD-47507, EMD-47508, EMD-47509, EMD-47510, EMD-47511, EMD-47512, and EMD-47513, respectively. Additional data for the electrophysiology experiments presented in Fig. 6 and Extended data Fig. 1 are provided through a figshare repository (10.6084/m9.figshare.32680923)^64^. Source data are provided with this paper.

## References

[1] Du, J. et al. Protons are a neurotransmitter that regulates synaptic plasticity in the lateral amygdala. *Proc. Natl Acad. Sci. USA* **111**, 8961–8966 (2014). 24889629 10.1073/pnas.1407018111 PMC4066526

[2] Wemmie, J. A. et al. The acid-activated ion channel ASIC contributes to synaptic plasticity, learning, and memory. *Neuron* **34**, 463–477 (2002). 11988176 10.1016/s0896-6273(02)00661-x

[3] Wemmie, J. A. et al. Acid-sensing ion channel 1 is localized in brain regions with high synaptic density and contributes to fear conditioning. *J. Neurosci.* **23**, 5496–5502 (2003). 12843249 10.1523/JNEUROSCI.23-13-05496.2003 PMC6741257

[4] Gao, J. et al. Coupling between NMDA receptor and acid-sensing ion channel contributes to ischemic neuronal death. *Neuron* **48**, 635–646 (2005). 16301179 10.1016/j.neuron.2005.10.011

[5] Xiong, Z. G. et al. Neuroprotection in ischemia: blocking calcium-permeable acid-sensing ion channels. *Cell* **118**, 687–698 (2004). 15369669 10.1016/j.cell.2004.08.026

[6] Redd, M. A. et al. Therapeutic inhibition of acid-sensing ion channel 1a recovers heart function after ischemia–reperfusion injury. *Circulation* **144**, 947–960 (2021). 34264749 10.1161/CIRCULATIONAHA.121.054360

[7] Bohlen, C. J. et al. A heteromeric Texas coral snake toxin targets acid-sensing ion channels to produce pain. *Nature* **479**, 410–414 (2011). 22094702 10.1038/nature10607 PMC3226747

[8] Heusser, S. A. & Pless, S. A. Acid-sensing ion channels as potential therapeutic targets. *Trends Pharmacol. Sci.* **42**, 1035–1050 (2021). 34674886 10.1016/j.tips.2021.09.008

[9] Hesselager, M., Timmermann, D. B. & Ahring, P. K. pH dependency and desensitization kinetics of heterologously expressed combinations of acid-sensing ion channel subunits. *J. Biol. Chem.* **279**, 11006–11015 (2004). 14701823 10.1074/jbc.M313507200

[10] Baconguis, I., Bohlen, C. J., Goehring, A., Julius, D. & Gouaux, E. X-ray structure of acid-sensing ion channel 1-snake toxin complex reveals open state of a Na^+^-selective channel. *Cell* **156**, 717–729 (2014). 24507937 10.1016/j.cell.2014.01.011 PMC4190031

[11] Jasti, J., Furukawa, H., Gonzales, E. B. & Gouaux, E. Structure of acid-sensing ion channel 1 at 1.9 Å resolution and low pH. *Nature* **449**, 316–323 (2007). 17882215 10.1038/nature06163

[12] Yoder, N. & Gouaux, E. The His-Gly motif of acid-sensing ion channels resides in a reentrant ‘loop’ implicated in gating and ion selectivity. *eLife* **9**, e56527 (2020). 32496192 10.7554/eLife.56527 PMC7308080

[13] Yoder, N., Yoshioka, C. & Gouaux, E. Gating mechanisms of acid-sensing ion channels. *Nature* **555**, 397–401 (2018). 29513651 10.1038/nature25782 PMC5966032

[14] Kellenberger, S., Auberson, M., Gautschi, I., Schneeberger, E. & Schild, L. Permeability properties of ENaC selectivity filter mutants. *J. Gen. Physiol.* **118**, 679–692 (2001). 11723161 10.1085/jgp.118.6.679 PMC2229513

[15] Kellenberger, S. & Schild, L. Epithelial sodium channel/degenerin family of ion channels: a variety of functions for a shared structure. *Physiol. Rev.* **82**, 735–767 (2002). 12087134 10.1152/physrev.00007.2002

[16] Yang, L. & Palmer, L. G. Determinants of selective ion permeation in the epithelial Na^+^ channel. *J. Gen. Physiol.* **150**, 1397–1407 (2018). 30135076 10.1085/jgp.201812164 PMC6168236

[17] Lynagh, T. et al. A selectivity filter at the intracellular end of the acid-sensing ion channel pore. *eLife* **6**, e24630 (2017). 10.7554/eLife.24630 PMC5449180 28498103

[18] Cristofori-Armstrong, B. & Rash, L. D. Acid-sensing ion channel (ASIC) structure and function: insights from spider, snake and sea anemone venoms. *Neuropharmacology* **127**, 173–184 (2017). 28457973 10.1016/j.neuropharm.2017.04.042

[19] Baconguis, I. & Gouaux, E. Structural plasticity and dynamic selectivity of acid-sensing ion channel–spider toxin complexes. *Nature* **489**, 400–405 (2012). 22842900 10.1038/nature11375 PMC3725952

[20] Dawson, R. J. et al. Structure of the acid-sensing ion channel 1 in complex with the gating modifier Psalmotoxin 1. *Nat. Commun.* **3**, 936 (2012). 22760635 10.1038/ncomms1917

[21] Sun, D. et al. Structural insights into human acid-sensing ion channel 1a inhibition by snake toxin mambalgin1. *eLife* **9**, e57096 (2020). 32915133 10.7554/eLife.57096 PMC7553779

[22] Yoder, N. & Gouaux, E. Divalent cation and chloride ion sites of chicken acid sensing ion channel 1a elucidated by X-ray crystallography. *PLoS ONE* **13**, e0202134 (2018). 30157194 10.1371/journal.pone.0202134 PMC6114778

[23] Larson, M. C., Luthi, M. R., Hogg, N. & Hillery, C. A. Calcium-phosphate microprecipitates mimic microparticles when examined with flow cytometry. *Cytometry A* **83**, 242–250 (2013). 23125136 10.1002/cyto.a.22222 PMC3615643

[24] Zhang, P., Sigworth, F. J. & Canessa, C. M. Gating of acid-sensitive ion channel-1: release of Ca^2+^ block vs. allosteric mechanism. *J. Gen. Physiol.* **127**, 109–117 (2006). 16418400 10.1085/jgp.200509396 PMC2151491

[25] Wu, Y., Chen, Z., Sigworth, F. J. & Canessa, C. M. Structure and analysis of nanobody binding to the human ASIC1a ion channel. *eLife* **10**, e67115 (2021). 34319232 10.7554/eLife.67115 PMC8318589

[26] Liu, Y. et al. Molecular mechanism and structural basis of small-molecule modulation of the gating of acid-sensing ion channel 1. *Commun. Biol.* **4**, 174 (2021). 33564124 10.1038/s42003-021-01678-1 PMC7873226

[27] Coscoy, S., de Weille, J. R., Lingueglia, E. & Lazdunski, M. The pre-transmembrane 1 domain of acid-sensing ion channels participates in the ion pore. *J. Biol. Chem.* **274**, 10129–10132 (1999). 10187795 10.1074/jbc.274.15.10129

[28] Sheikh, Z. P. et al. The M1 and pre-M1 segments contribute differently to ion selectivity in ASICs and ENaCs. *J. Gen. Physiol.* **153**, e202112899 (2021). 34436511 10.1085/jgp.202112899 PMC8404453

[29] Pfister, Y. et al. A gating mutation in the internal pore of ASIC1a. *J. Biol. Chem.* **281**, 11787–11791 (2006). 16497675 10.1074/jbc.M513692200

[30] Bassler, E. L., Ngo-Anh, T. J., Geisler, H. S., Ruppersberg, J. P. & Grunder, S. Molecular and functional characterization of acid-sensing ion channel (ASIC) 1b. *J. Biol. Chem.* **276**, 33782–33787 (2001). 11448963 10.1074/jbc.M104030200

[31] Smart, O. S., Neduvelil, J. G., Wang, X., Wallace, B. A. & Sansom, M. S. HOLE: a program for the analysis of the pore dimensions of ion channel structural models. *J. Mol. Graph.* **14**, 354–360 (1996). 9195488 10.1016/s0263-7855(97)00009-x

[32] Yang, H. et al. Inherent dynamics of the acid-sensing ion channel 1 correlates with the gating mechanism. *PLoS Biol.* **7**, e1000151 (2009). 19597538 10.1371/journal.pbio.1000151 PMC2701601

[33] Bandarupalli, R., Roth, R., Klipp, R. C., Bankston, J. R. & Li, J. Molecular insights into single-chain lipid modulation of acid-sensing ion channel 3. *J. Phys. Chem. B* **128**, 12685–12697 (2024). 39666997 10.1021/acs.jpcb.4c04289 PMC12885156

[34] Li, M., Toombes, G. E., Silberberg, S. D. & Swartz, K. J. Physical basis of apparent pore dilation of ATP-activated P2X receptor channels. *Nat. Neurosci.* **18**, 1577–1583 (2015). 26389841 10.1038/nn.4120 PMC5113834

[35] Yang, Y. L. & Lai, T. W. Native expression of ASIC1a and ASIC1b human homologues in the HEK 293 cell line allows pharmacological evaluation of analgesics targeting acid sensation in humans. *Neuroreport* **31**, 865–870 (2020). 32453026 10.1097/WNR.0000000000001465

[36] Bonifacio, G., Lelli, C. I. & Kellenberger, S. Protonation controls ASIC1a activity via coordinated movements in multiple domains. *J. Gen. Physiol.* **143**, 105–118 (2014). 24344244 10.1085/jgp.201311053 PMC3874563

[37] Borg, C. B. et al. Mechanism and site of action of big dynorphin on ASIC1a. *Proc. Natl Acad. Sci. USA* **117**, 7447–7454 (2020). 32165542 10.1073/pnas.1919323117 PMC7132280

[38] Heusser, S. A., Borg, C. B., Colding, J. M. & Pless, S. A. Conformational decoupling in acid-sensing ion channels uncovers mechanism and stoichiometry of PcTx1-mediated inhibition. *eLife* **11**, e73384 (2022). 35156612 10.7554/eLife.73384 PMC8871370

[39] Krishtal, O. A. & Pidoplichko, V. I. Receptor for protons in the membrane of sensory neurons. *Brain Res.* **214**, 150–154 (1981). 6263415 10.1016/0006-8993(81)90446-7

[40] Holm, C. M., Topaktas, A. B., Dannesboe, J., Pless, S. A. & Heusser, S. A. Dynamic conformational changes of acid-sensing ion channels in different desensitizing conditions. *Biophys. J.* **123**, 2122–2135 (2024). 38549370 10.1016/j.bpj.2024.03.038 PMC11309988

[41] Springauf, A., Bresenitz, P. & Grunder, S. The interaction between two extracellular linker regions controls sustained opening of acid-sensing ion channel 1. *J. Biol. Chem.* **286**, 24374–24384 (2011). 21576243 10.1074/jbc.M111.230797 PMC3129216

[42] Li, T., Yang, Y. & Canessa, C. M. Impact of recovery from desensitization on acid-sensing ion channel-1a (ASIC1a) current and response to high frequency stimulation. *J. Biol. Chem.* **287**, 40680–40689 (2012). 23048040 10.1074/jbc.M112.418400 PMC3504781

[43] Gonzales, E. B., Kawate, T. & Gouaux, E. Pore architecture and ion sites in acid-sensing ion channels and P2X receptors. *Nature* **460**, 599–604 (2009). 19641589 10.1038/nature08218 PMC2845979

[44] Chen, X., Kalbacher, H. & Grunder, S. Interaction of acid-sensing ion channel (ASIC) 1 with the tarantula toxin psalmotoxin 1 is state dependent. *J. Gen. Physiol.* **127**, 267–276 (2006). 16505147 10.1085/jgp.200509409 PMC2151504

[45] Saez, N. J. et al. A dynamic pharmacophore drives the interaction between psalmotoxin-1 and the putative drug target acid-sensing ion channel 1a. *Mol. Pharmacol.* **80**, 796–808 (2011). 21825095 10.1124/mol.111.072207

[46] Cristofori-Armstrong, B., Saez, N. J., Chassagnon, I. R., King, G. F. & Rash, L. D. The modulation of acid-sensing ion channel 1 by PcTx1 is pH-, subtype- and species-dependent: Importance of interactions at the channel subunit interface and potential for engineering selective analogues. *Biochem. Pharmacol.* **163**, 381–390 (2019). 30849303 10.1016/j.bcp.2019.03.004

[47] Chen, X. & Grunder, S. Permeating protons contribute to tachyphylaxis of the acid-sensing ion channel (ASIC) 1a. *J. Physiol.* **579**, 657–670 (2007). 17204502 10.1113/jphysiol.2006.120733 PMC2151377

[48] Komarova, M. S., Bukharev, A. R., Potapieva, N. N. & Tikhonov, D. B. Modulation of slow desensitization (tachyphylaxis) of acid-sensing ion channel (ASIC)1a. *Cell. Mol. Neurobiol.* **43**, 771–783 (2023). 35201495 10.1007/s10571-022-01207-6 PMC11415197

[49] Gitterman, D. P., Wilson, J. & Randall, A. D. Functional properties and pharmacological inhibition of ASIC channels in the human SJ-RH30 skeletal muscle cell line. *J. Physiol.* **562**, 759–769 (2005). 15576453 10.1113/jphysiol.2004.075069 PMC1665529

[50] Carbone, A. L. & Plested, A. J. Coupled control of desensitization and gating by the ligand binding domain of glutamate receptors. *Neuron* **74**, 845–857 (2012). 22681689 10.1016/j.neuron.2012.04.020

[51] Grunder, S. & Pusch, M. Biophysical properties of acid-sensing ion channels (ASICs). *Neuropharmacology* **94**, 9–18 (2015). 25585135 10.1016/j.neuropharm.2014.12.016

[52] Noreng, S., Bharadwaj, A., Posert, R., Yoshioka, C. & Baconguis, I. Structure of the human epithelial sodium channel by cryo-electron microscopy. *eLife* **7**, e39340 (2018). 30251954 10.7554/eLife.39340 PMC6197857

[53] Kalienkova, V., Dandamudi, M., Paulino, C. & Lynagh, T. Structural basis for excitatory neuropeptide signaling. *Nat. Struct. Mol. Biol.* **31**, 717–726 (2024). 38337033 10.1038/s41594-023-01198-y PMC11026163

[54] Liu, F. et al. Structure and mechanism of a neuropeptide-activated channel in the ENaC/DEG superfamily. *Nat. Chem. Biol.* **19**, 1276–1285 (2023). 37550431 10.1038/s41589-023-01401-7

[55] Goehring, A. et al. Screening and large-scale expression of membrane proteins in mammalian cells for structural studies. *Nat. Protoc.* **9**, 2574–2585 (2014). 25299155 10.1038/nprot.2014.173 PMC4291175

[56] Punjani, A., Rubinstein, J. L., Fleet, D. J. & Brubaker, M. A. cryoSPARC: algorithms for rapid unsupervised cryo-EM structure determination. *Nat. Methods* **14**, 290–296 (2017). 28165473 10.1038/nmeth.4169

[57] Punjani, A., Zhang, H. & Fleet, D. J. Non-uniform refinement: adaptive regularization improves single-particle cryo-EM reconstruction. *Nat. Methods* **17**, 1214–1221 (2020). 33257830 10.1038/s41592-020-00990-8

[58] Emsley, P., Lohkamp, B., Scott, W. G. & Cowtan, K. Features and development of Coot. *Acta Crystallogr. D* **66**, 486–501 (2010). 20383002 10.1107/S0907444910007493 PMC2852313

[59] Pettersen, E. F. et al. UCSF ChimeraX: structure visualization for researchers, educators, and developers. *Protein Sci* **30**, 70–82 (2021). 32881101 10.1002/pro.3943 PMC7737788

[60] Croll, T. I. ISOLDE: a physically realistic environment for model building into low-resolution electron-density maps. *Acta Crystallogr. D* **74**, 519–530 (2018). 10.1107/S2059798318002425 PMC6096486 29872003

[61] Afonine, P. V. et al. Real-space refinement in PHENIX for cryo-EM and crystallography. *Acta Crystallogr. D* **74**, 531–544 (2018). 10.1107/S2059798318006551 PMC6096492 29872004

[62] Liebschner, D. et al. Macromolecular structure determination using X-rays, neutrons and electrons: recent developments in PHENIX. *Acta Crystallogr. D* **75**, 861–877 (2019). 10.1107/S2059798319011471 PMC6778852 31588918

[63] Williams, C. J. et al. MolProbity: more and better reference data for improved all-atom structure validation. *Protein Sci.* **27**, 293–315 (2018). 29067766 10.1002/pro.3330 PMC5734394

[64] Cahill, J, Hartfield, K. A. et al. Conformational plasticity of human acid-sensing ion channel 1a. *figshare*10.6084/m9.figshare.32680923 (2026). 10.1038/s41594-026-01845-0 PMC13468109 42463865

