# Supplementary material for: Conformational plasticity of human acid-sensing ion channel 1a
Source: Nat Struct Mol Biol. 2026 Jul 16;33(8):1171–82. doi: 10.1038/s41594-026-01845-0 (PMC13468109; doi:10.1038/s41594-026-01845-0)
Supplement: Supplementary file 1 [file 41594_2026_1845_MOESM1_ESM.pdf]

nature structural & molecular biology

Supplementary information https://doi.org/10.1038/s41594-026-01845-0

Conformational plasticity of human acid-sensing ion channel 1a

In the format provided by the authors and unedited

Conformational plasticity of human acid-sensing ion channel 1a

James Cahill[1,5], Kimberly A. Hartfield[1,5], Stephanie Andrea Heusser[2,4], Nadine Ritter[2], Mette Homann Poulsen[2], Craig Yoshioka[3], Stephan Alexander Pless[2], Isabelle Baconguis[1,6]

[1]Vollum Institute, Oregon Health & Science University, Portland, OR 97239

[2]University of Copenhagen, Department of Drug Design and Pharmacology, Jagtvej 160, 2100 Copenhagen, Denmark

[3]Department of Biomedical Engineering, Oregon Health & Science University, Portland, OR 97239

[4]Linköping University, Department of Biomedical and Clinical Sciences, 58225 Linköping, Sweden

[5]These authors contributed equally

[6]Correspondence: Isabelle Baconguis, bacongui@ohsu.edu

This PDF file includes:

Supplementary Data Figures 1-3, Supplementary Tables 1-3, Raw gel image

Table of Contents	Title	Page
Supplementary Data Figure 1	Sample preparation of hASIC1a for cryo-EM analysis	3
Supplementary Data Figure 2	Cryo-EM data processing strategy	5
Supplementary Data Figure 3	Cryo-EM map-model agreement	13
Supplementary Table 1	Summary of electrophysiology data	20
Supplementary Table 2	PDB and EMDB codes of hASIC1a structures	21
Supplementary Table 3	Cryo-EM data collection, refinement, model refinement and validation statistics	22
Raw gel image	Raw data for uncropped Coomassie-stained gel	25

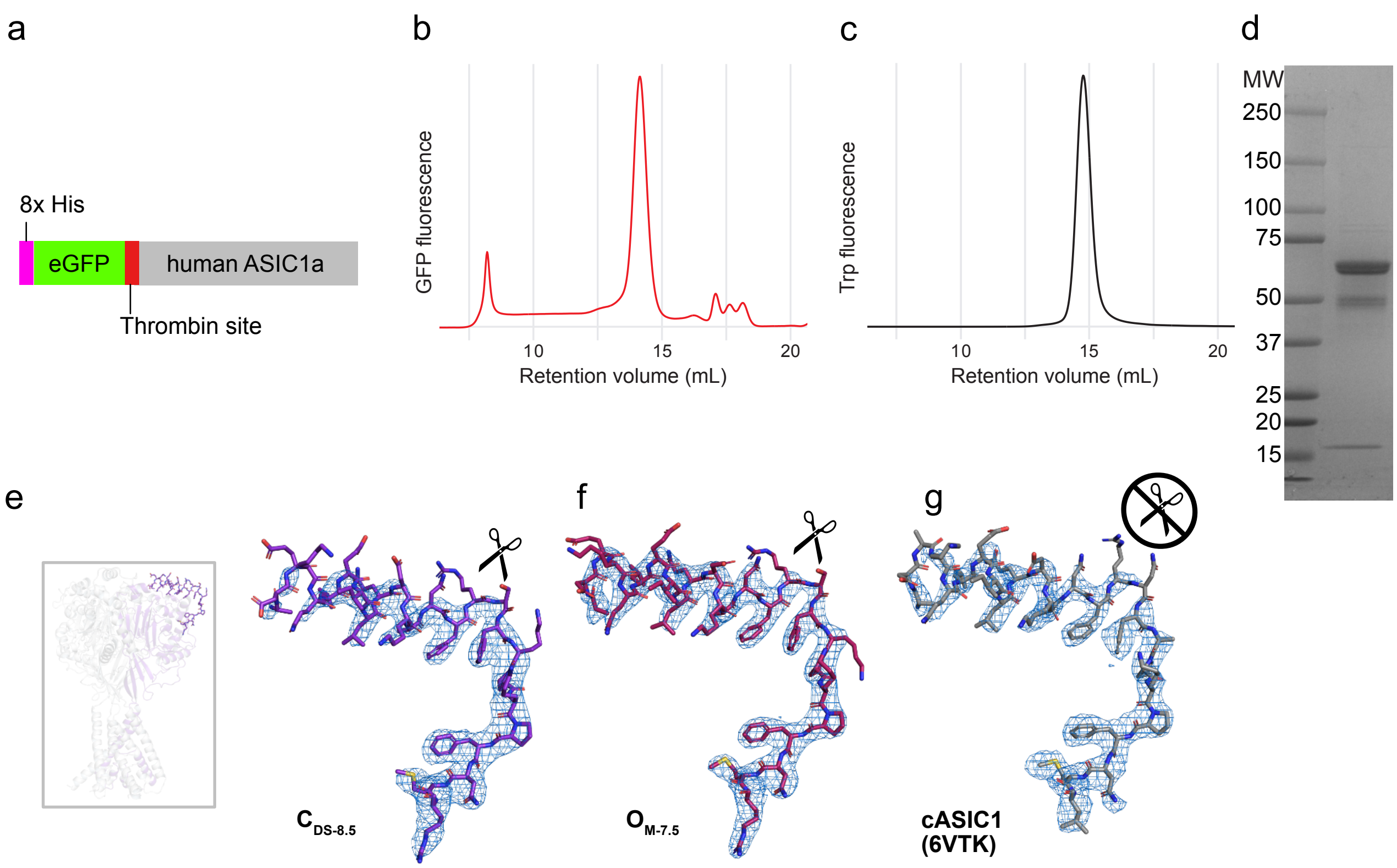

Supplementary Figure 1

Supplementary Data Figure 1: Sample preparation of hASIC1a for cryo-EM analysis

a. The hASIC1a expression construct has a N-terminal 8xHis-eGFP tag which can be removed by thrombin cleavage. **b**. FSEC chromatogram of proteins extracted from HEK293S GnTI$^-$ cells expressing GFP-tagged hASIC1a. GFP fluorescence is monitored due to the abundance of non-fluorescent cellular proteins within the sample. **c**. FSEC chromatogram of purified hASIC1a demonstrates a homogenous sample. Trp fluorescence is monitored due to removal of GFP during purification. **d**. SDS-PAGE gel of purified hASIC1a. The larger band at approximately 65 kDa is consistent with the size of hASIC1a; the bands at approximately 50 and 15 kDa were identified as hASIC1a fragments by N-terminal sequencing. **e-f**. Maps of $C_{DS\text{-}8.5}$ and $O_{M\text{-}7.5}$ (blue mesh) show weakness in the vicinity of Ser146, which is the N-terminal residue of the post-cleavage fragment. However, maps depict the side chains of neighboring Phe144 and Phe147, anchoring the model such that an intact Phe-Arg-Ser-Phe peptide can be modeled in spite of potential partial cleavage. Inset: The cleavage site vicinity is depicted as sticks in the context of the hASIC1a trimer. **g.** Although no cleavage was suggested to occur between Arg146 and Asn147 of cASIC1[12], a similar map weakness is shown in desensitized cASIC1 (PDB ID: 6VTK) despite otherwise high map quality in the region.

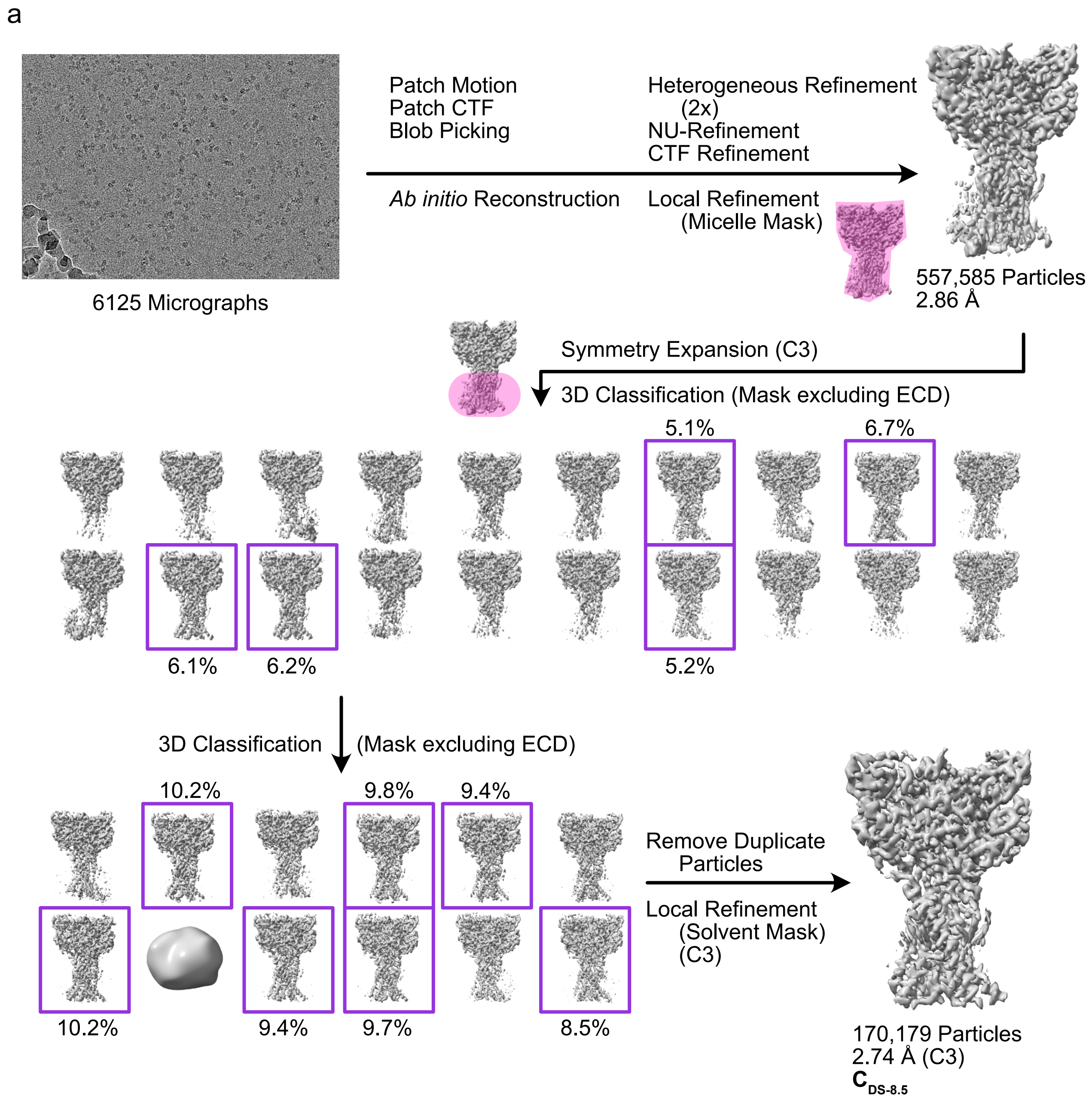
a
Patch Motion
Patch CTF
Blob Picking
Ab initio Reconstruction
Heterogeneous Refinement (2x)
NU-Refinement
CTF Refinement
Local Refinement (Micelle Mask)
6125 Micrographs
557,585 Particles
2.86 Å
Symmetry Expansion (C3)
3D Classification (Mask excluding ECD)
5.1%
6.7%
6.1%
6.2%
5.2%
3D Classification (Mask excluding ECD)
10.2%
9.8%
9.4%
10.2%
9.4%
9.7%
8.5%
Remove Duplicate Particles
Local Refinement (Solvent Mask) (C3)
170,179 Particles
2.74 Å (C3)
$C_{DS-8.5}$

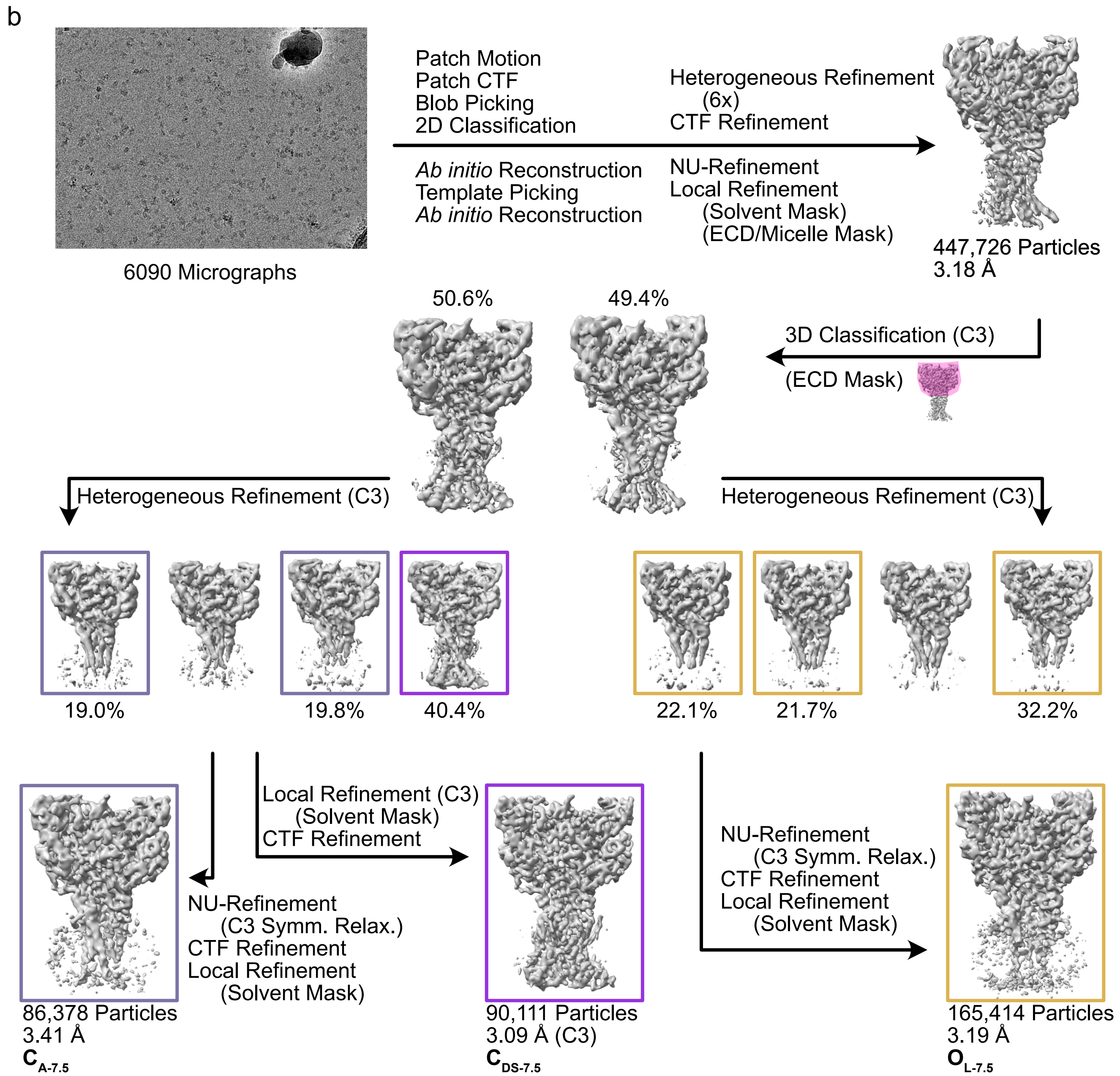
b
6090 Micrographs
Patch Motion
Patch CTF
Blob Picking
2D Classification
Ab initio Reconstruction
Template Picking
Ab initio Reconstruction
Heterogeneous Refinement
(6x)
CTF Refinement
NU-Refinement
Local Refinement
(Solvent Mask)
(ECD/Micelle Mask)
447,726 Particles
3.18 Å
3D Classification (C3)
(ECD Mask)
50.6%
49.4%
Heterogeneous Refinement (C3)
Heterogeneous Refinement (C3)
19.0%
19.8%
40.4%
22.1%
21.7%
32.2%
Local Refinement (C3)
(Solvent Mask)
CTF Refinement
NU-Refinement
(C3 Symm. Relax.)
CTF Refinement
Local Refinement
(Solvent Mask)
NU-Refinement
(C3 Symm. Relax.)
CTF Refinement
Local Refinement
(Solvent Mask)
86,378 Particles
3.41 Å
$C_{A-7.5}$
90,111 Particles
3.09 Å (C3)
$C_{DS-7.5}$
165,414 Particles
3.19 Å
$O_{L-7.5}$

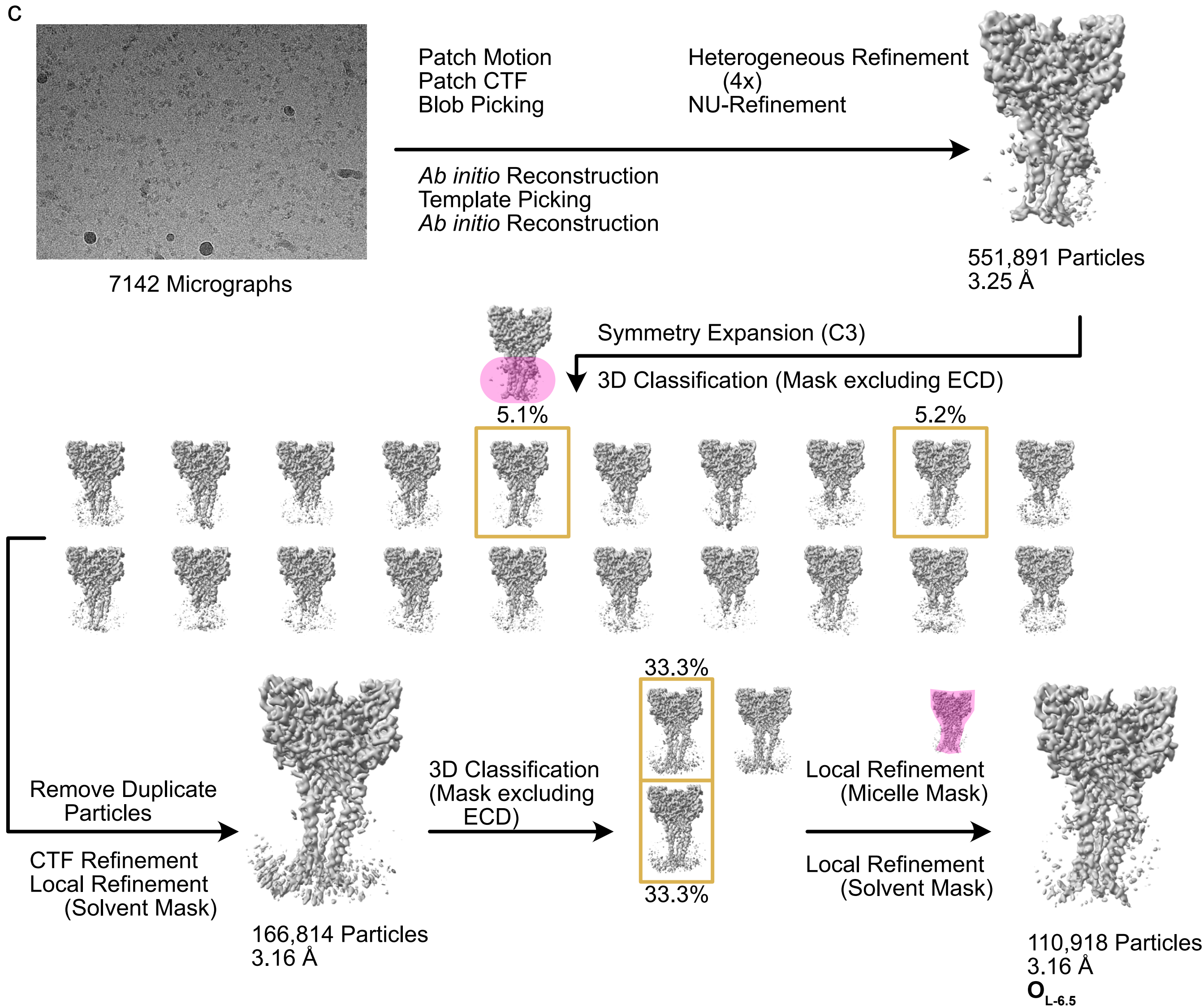
c
Patch Motion
Patch CTF
Blob Picking
Heterogeneous Refinement
(4x)
NU-Refinement
Ab initio Reconstruction
Template Picking
Ab initio Reconstruction
7142 Micrographs
551,891 Particles
3.25 Å
Symmetry Expansion (C3)
3D Classification (Mask excluding ECD)
5.1%
5.2%
Remove Duplicate
Particles
CTF Refinement
Local Refinement
(Solvent Mask)
166,814 Particles
3.16 Å
3D Classification
(Mask excluding
ECD)
33.3%
33.3%
Local Refinement
(Micelle Mask)
Local Refinement
(Solvent Mask)
110,918 Particles
3.16 Å
$\mathbf{O}_{L\text{-}6.5}$

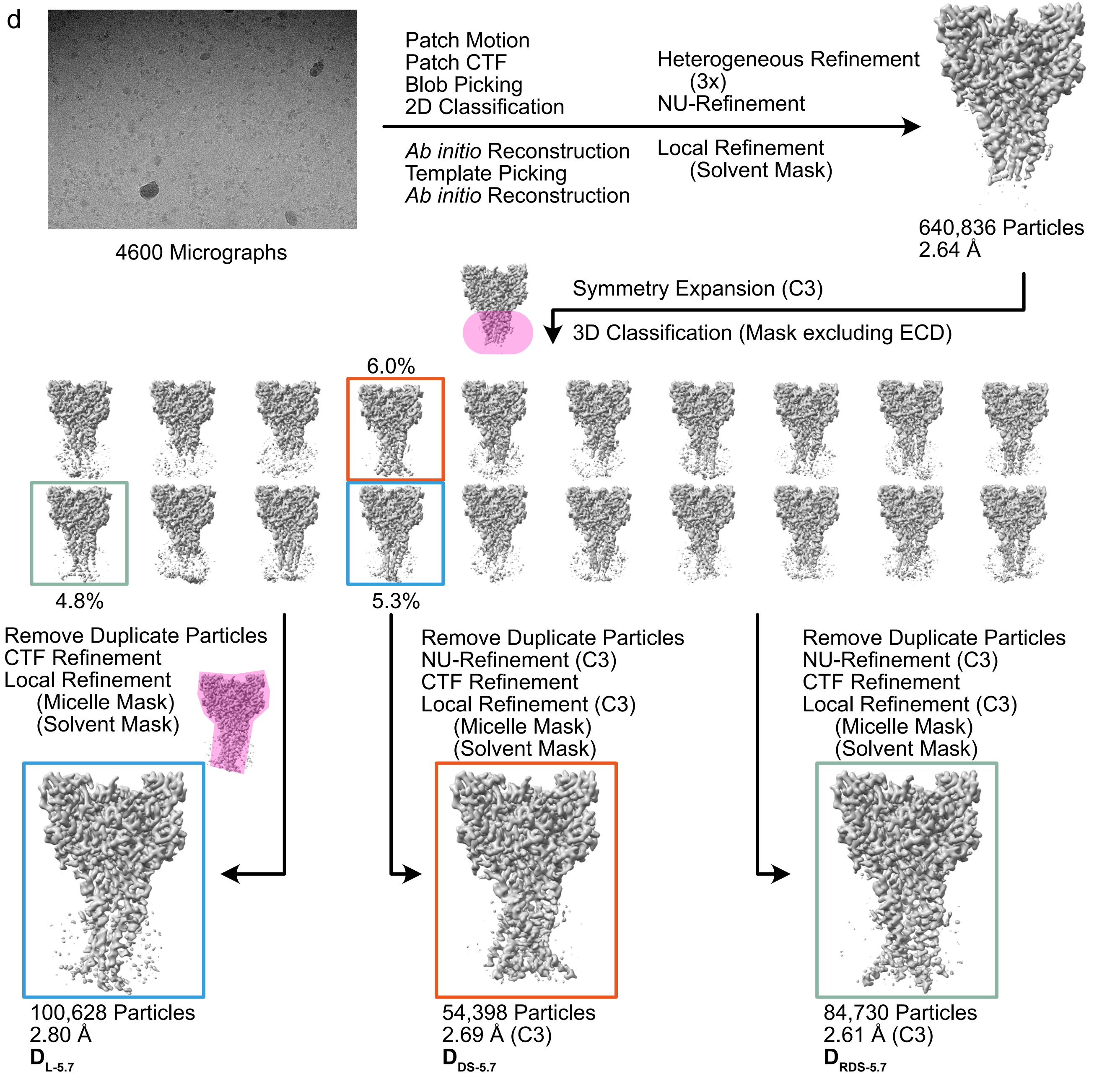
d
4600 Micrographs
Patch Motion
Patch CTF
Blob Picking
2D Classification
Ab initio Reconstruction
Template Picking
Ab initio Reconstruction
Heterogeneous Refinement
(3x)
NU-Refinement
Local Refinement
(Solvent Mask)
640,836 Particles
2.64 Å
Symmetry Expansion (C3)
3D Classification (Mask excluding ECD)
6.0%
4.8%
5.3%
Remove Duplicate Particles
CTF Refinement
Local Refinement
(Micelle Mask)
(Solvent Mask)
Remove Duplicate Particles
NU-Refinement (C3)
CTF Refinement
Local Refinement (C3)
(Micelle Mask)
(Solvent Mask)
Remove Duplicate Particles
NU-Refinement (C3)
CTF Refinement
Local Refinement (C3)
(Micelle Mask)
(Solvent Mask)
100,628 Particles
2.80 Å
$D_{L-5.7}$
54,398 Particles
2.69 Å (C3)
$D_{DS-5.7}$
84,730 Particles
2.61 Å (C3)
$D_{RDS-5.7}$

e

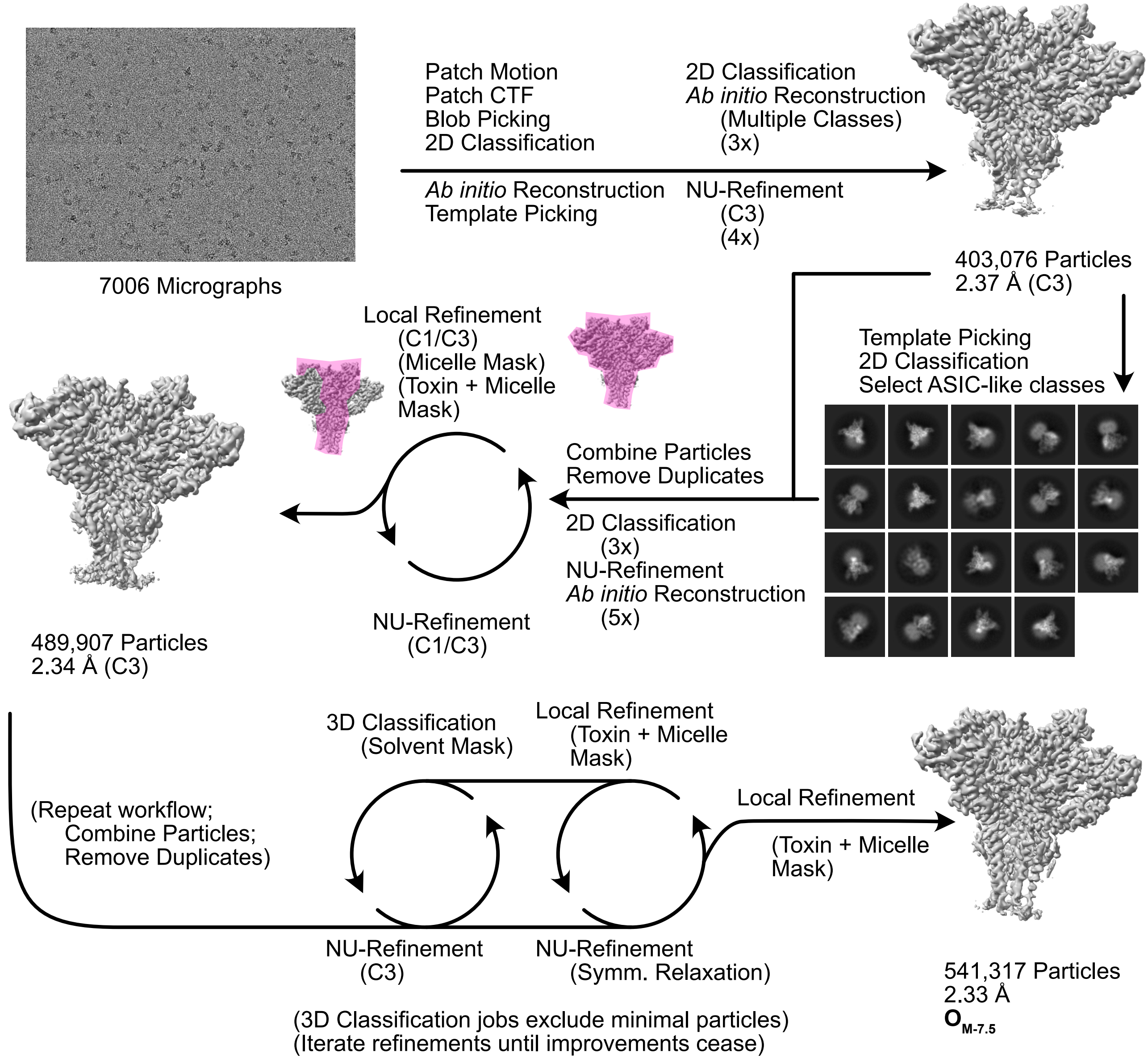
7006 Micrographs
Patch Motion
Patch CTF
Blob Picking
2D Classification
2D Classification
Ab initio Reconstruction
(Multiple Classes)
(3x)
Ab initio Reconstruction
Template Picking
NU-Refinement
(C3)
(4x)
403,076 Particles
2.37 Å (C3)
Template Picking
2D Classification
Select ASIC-like classes
Local Refinement
(C1/C3)
(Micelle Mask)
(Toxin + Micelle
Mask)
Combine Particles
Remove Duplicates
2D Classification
(3x)
NU-Refinement
Ab initio Reconstruction
(5x)
NU-Refinement
(C1/C3)
489,907 Particles
2.34 Å (C3)
3D Classification
(Solvent Mask)
Local Refinement
(Toxin + Micelle
Mask)
(Repeat workflow;
Combine Particles;
Remove Duplicates)
Local Refinement
(Toxin + Micelle
Mask)
NU-Refinement
(C3)
NU-Refinement
(Symm. Relaxation)
541,317 Particles
2.33 Å
O$_{M-7.5}$
(3D Classification jobs exclude minimal particles)
(Iterate refinements until improvements cease)

f

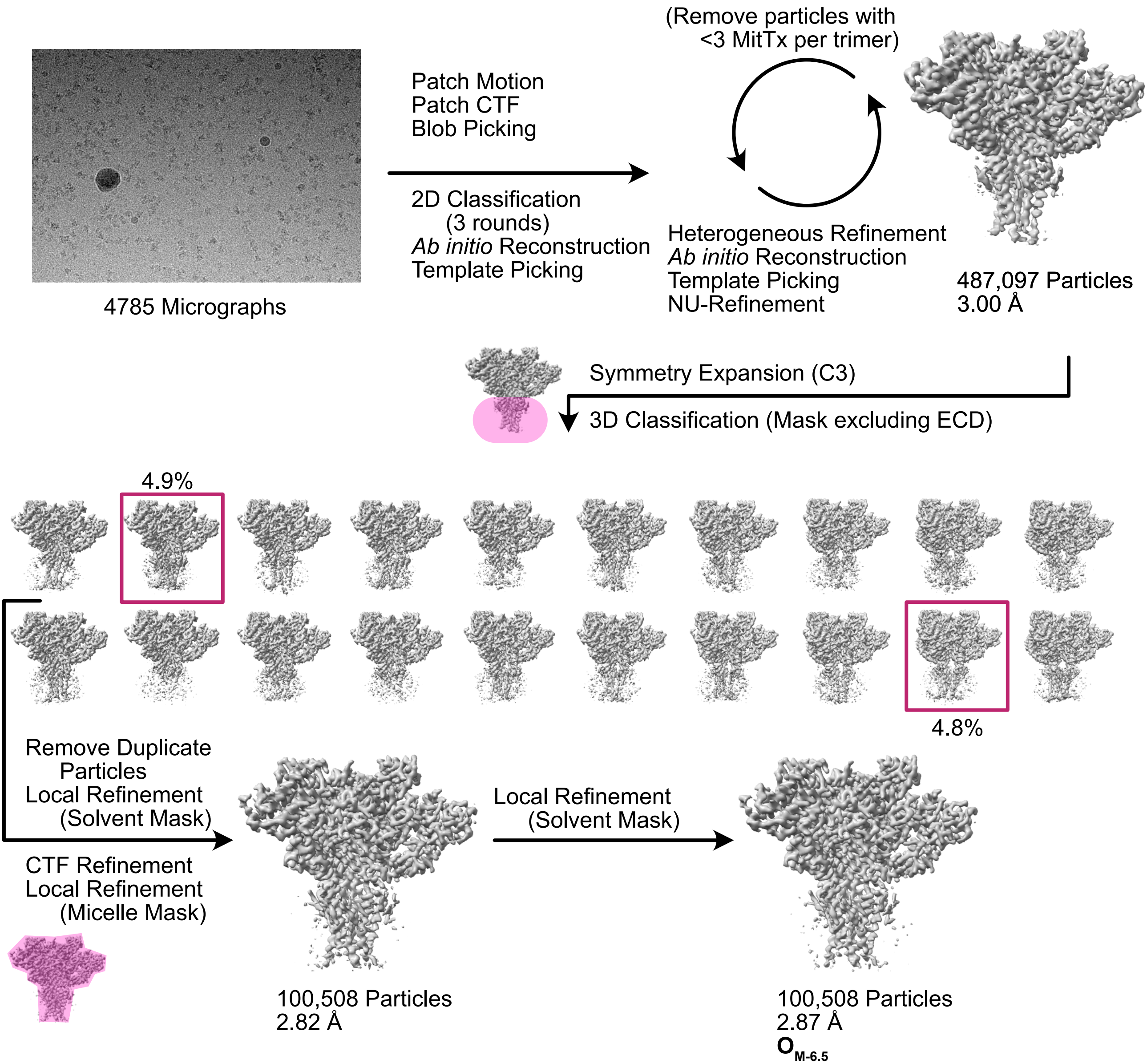

4785 Micrographs
Patch Motion
Patch CTF
Blob Picking
2D Classification
(3 rounds)
Ab initio Reconstruction
Template Picking
(Remove particles with
<3 MitTx per trimer)
Heterogeneous Refinement
Ab initio Reconstruction
Template Picking
NU-Refinement
487,097 Particles
3.00 Å
Symmetry Expansion (C3)
3D Classification (Mask excluding ECD)
4.9%
4.8%
Remove Duplicate
Particles
Local Refinement
(Solvent Mask)
CTF Refinement
Local Refinement
(Micelle Mask)
100,508 Particles
2.82 Å
Local Refinement
(Solvent Mask)
100,508 Particles
2.87 Å
$O_{M-6.5}$

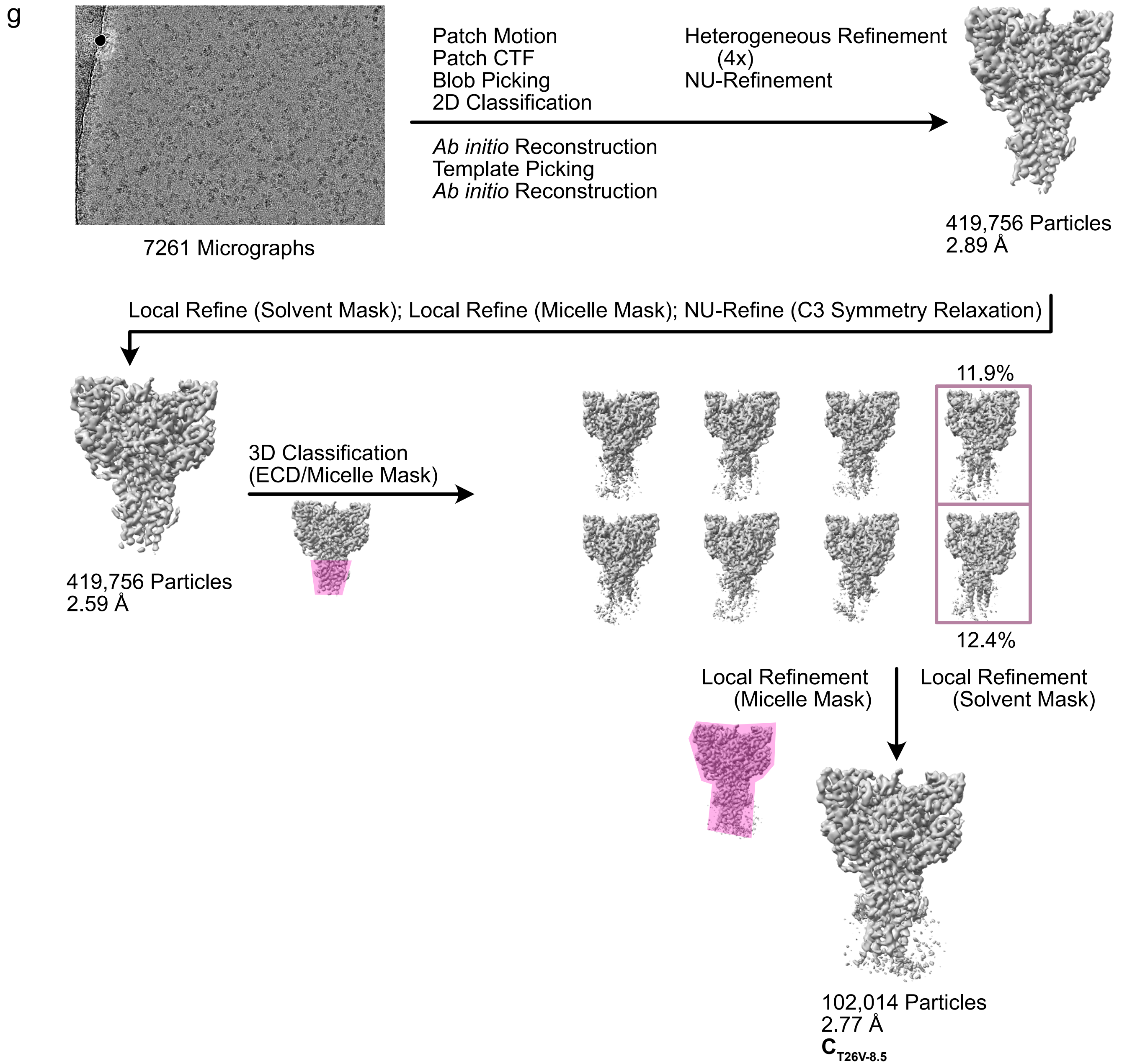

g
Patch Motion
Patch CTF
Blob Picking
2D Classification
Heterogeneous Refinement
(4x)
NU-Refinement
Ab initio Reconstruction
Template Picking
Ab initio Reconstruction
7261 Micrographs
419,756 Particles
2.89 Å
Local Refine (Solvent Mask); Local Refine (Micelle Mask); NU-Refine (C3 Symmetry Relaxation)
3D Classification
(ECD/Micelle Mask)
419,756 Particles
2.59 Å
11.9%
12.4%
Local Refinement
(Micelle Mask)
Local Refinement
(Solvent Mask)
102,014 Particles
2.77 Å
C T26V-8.5

Supplementary Data Figure 2: Cryo-EM data processing strategy

Schematic representation of the cryo-EM data processing workflow applied to the seven cryo-EM datasets using cryoSPARC.

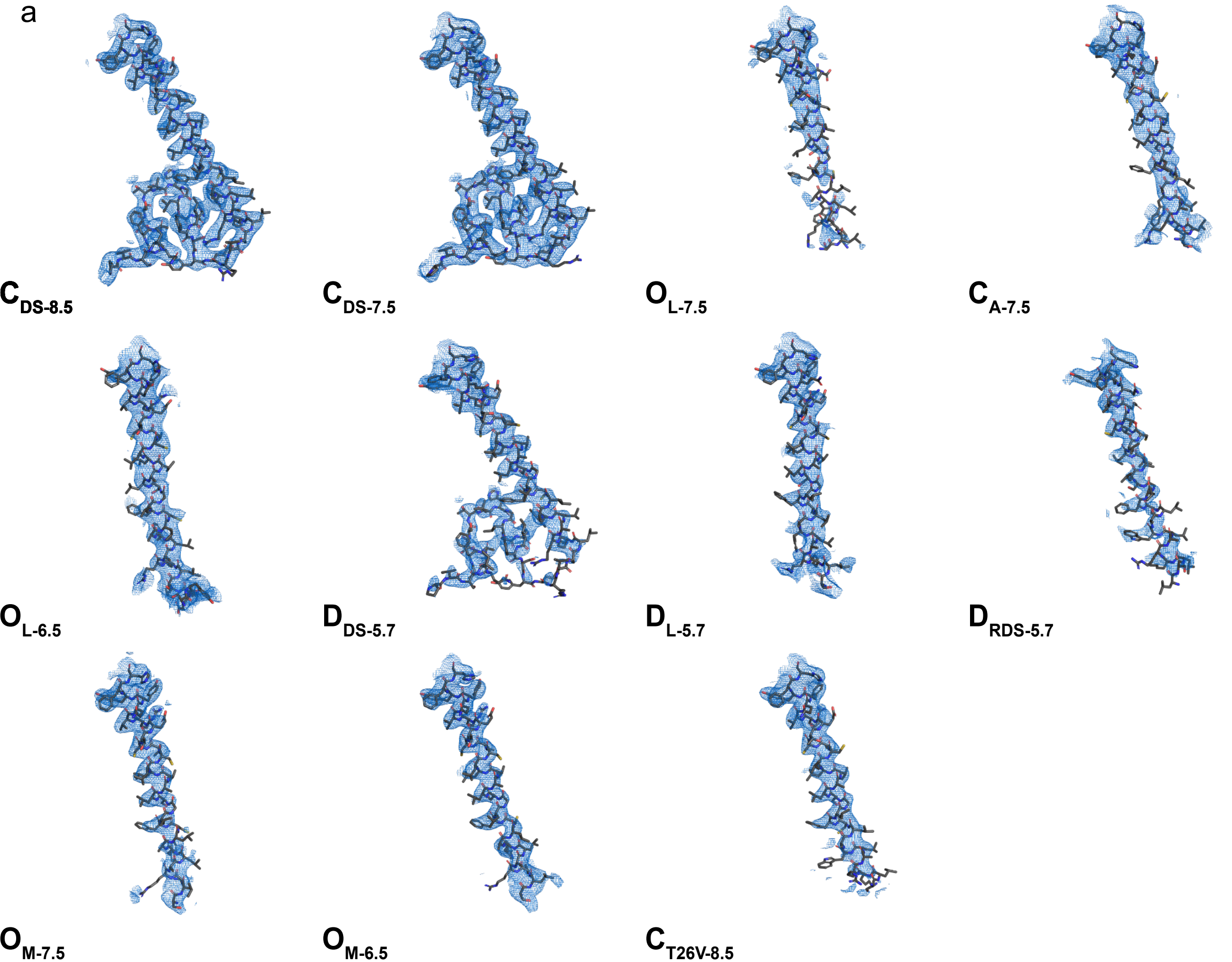
a
C DS-8.5
C DS-7.5
O L-7.5
C A-7.5
O L-6.5
D DS-5.7
D L-5.7
D RDS-5.7
O M-7.5
O M-6.5
C T26V-8.5

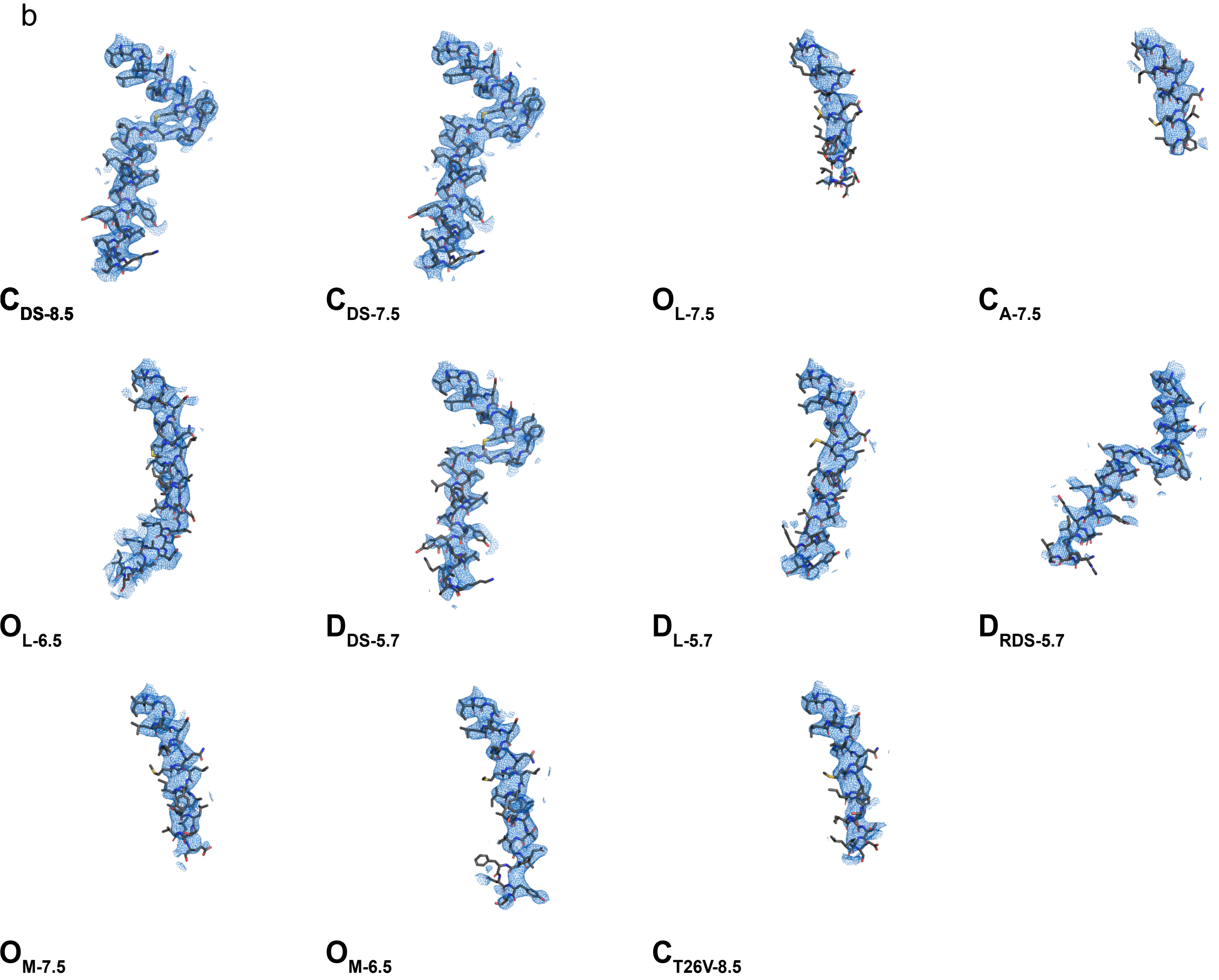
b
C DS-8.5
C DS-7.5
O L-7.5
C A-7.5
O L-6.5
D DS-5.7
D L-5.7
D RDS-5.7
O M-7.5
O M-6.5
C T26V-8.5

C

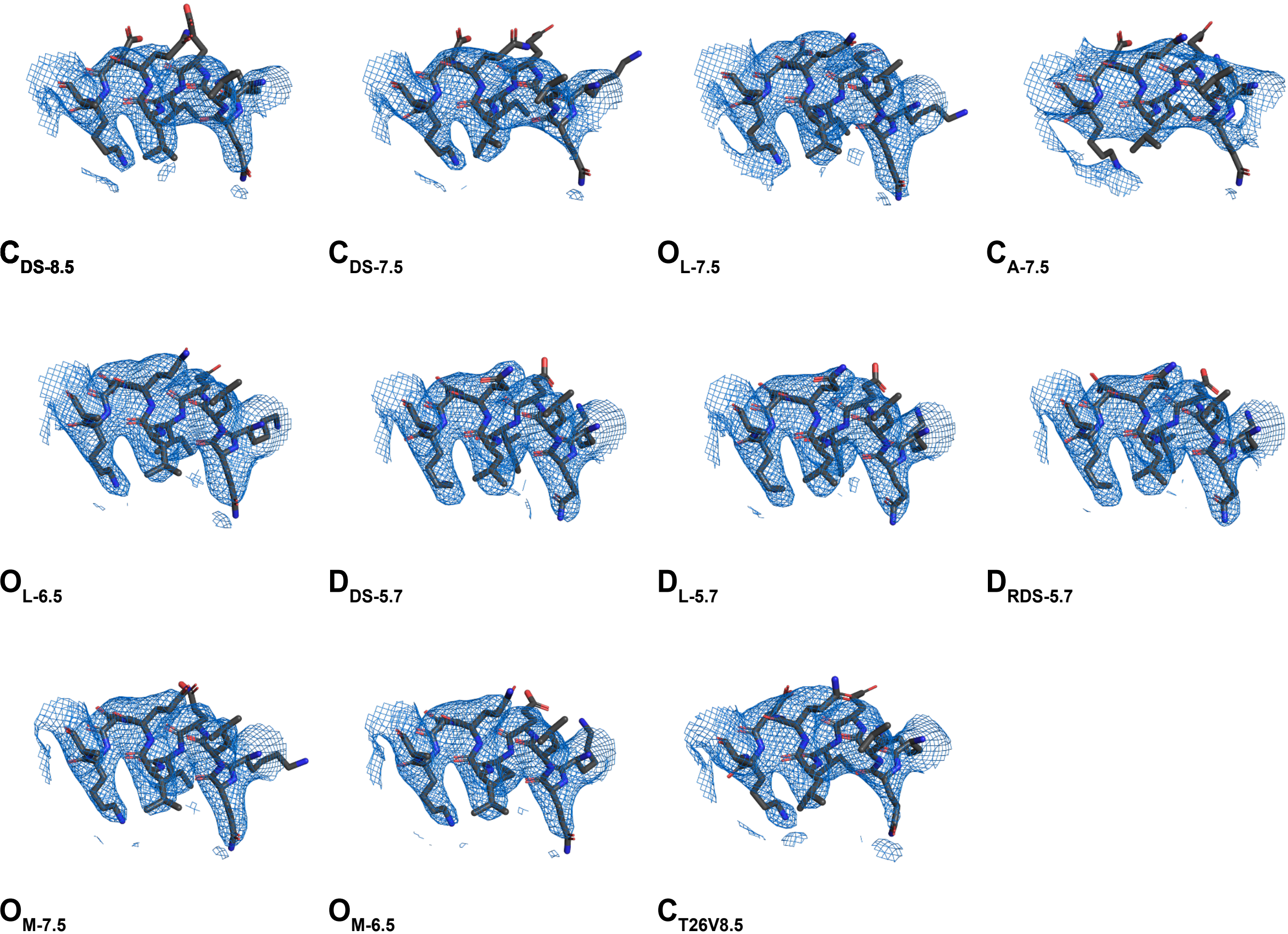

$C_{DS-8.5}$
$C_{DS-7.5}$
$O_{L-7.5}$
$C_{A-7.5}$
$O_{L-6.5}$
$D_{DS-5.7}$
$D_{L-5.7}$
$D_{RDS-5.7}$
$O_{M-7.5}$
$O_{M-6.5}$
$C_{T26V8.5}$

d

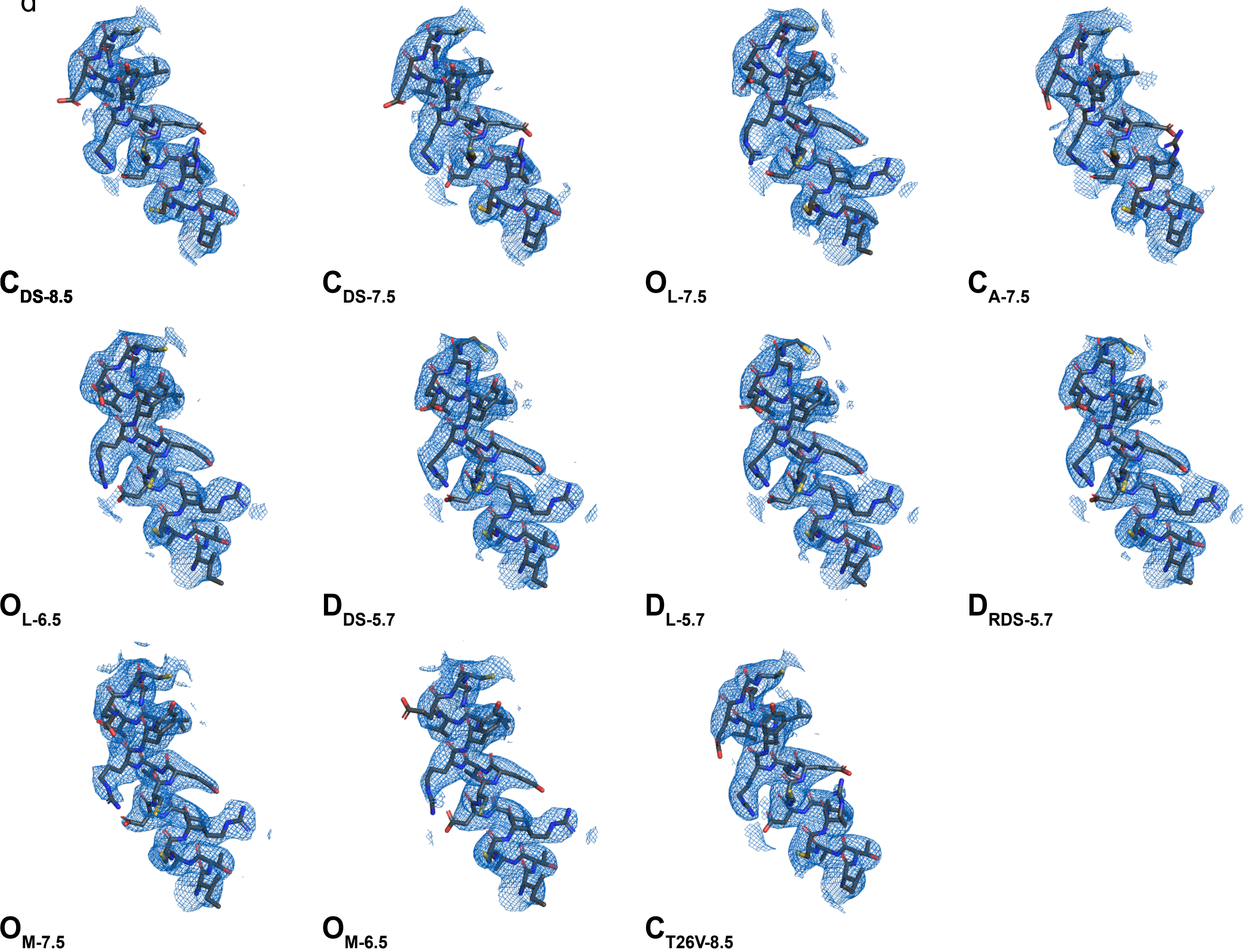
$C_{DS-8.5}$
$C_{DS-7.5}$
$O_{L-7.5}$
$C_{A-7.5}$
$O_{L-6.5}$
$D_{DS-5.7}$
$D_{L-5.7}$
$D_{RDS-5.7}$
$O_{M-7.5}$
$O_{M-6.5}$
$C_{T26V-8.5}$

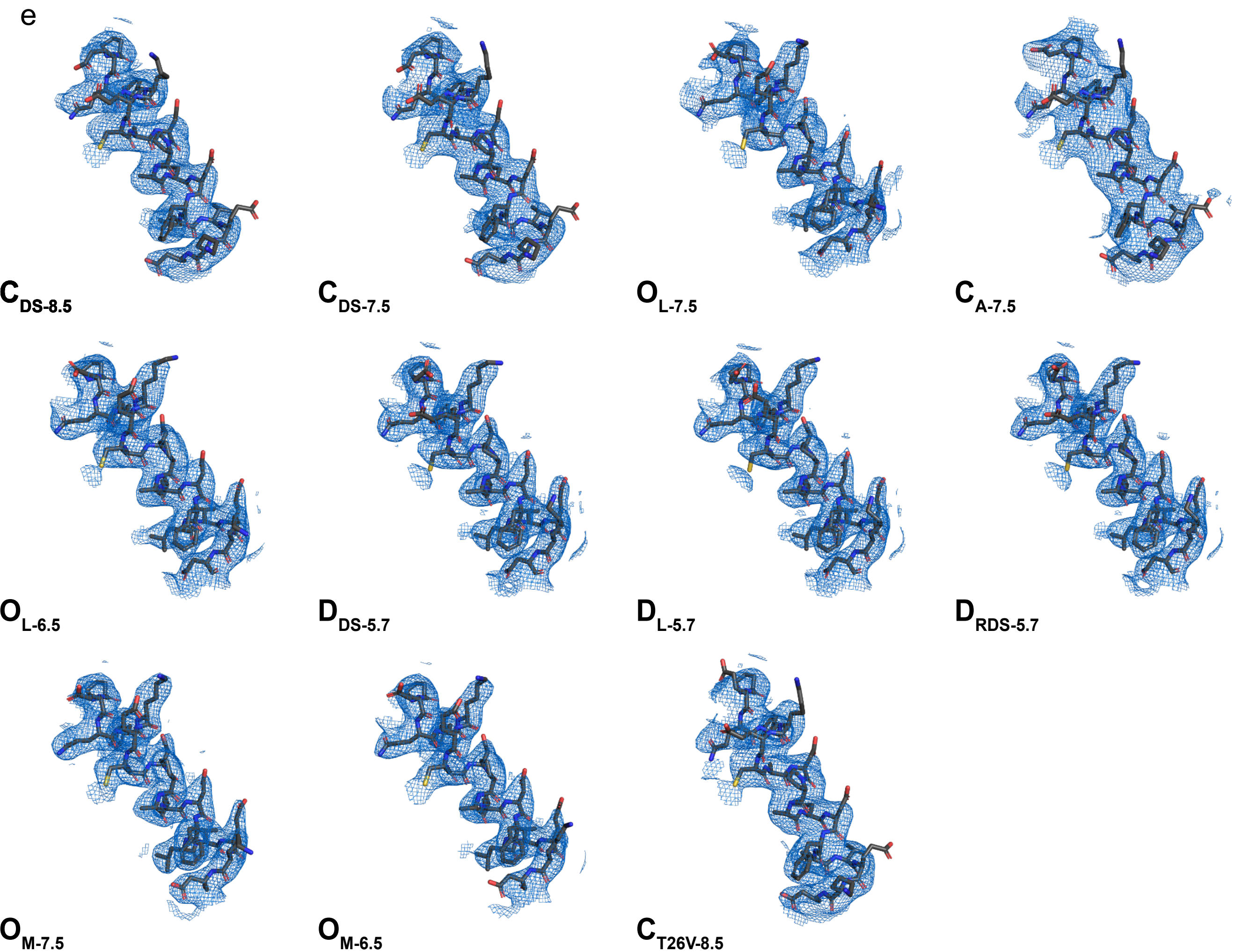
e
C DS-8.5
C DS-7.5
O L-7.5
C A-7.5
O L-6.5
D DS-5.7
D L-5.7
D RDS-5.7
O M-7.5
O M-6.5
C T26V-8.5

f

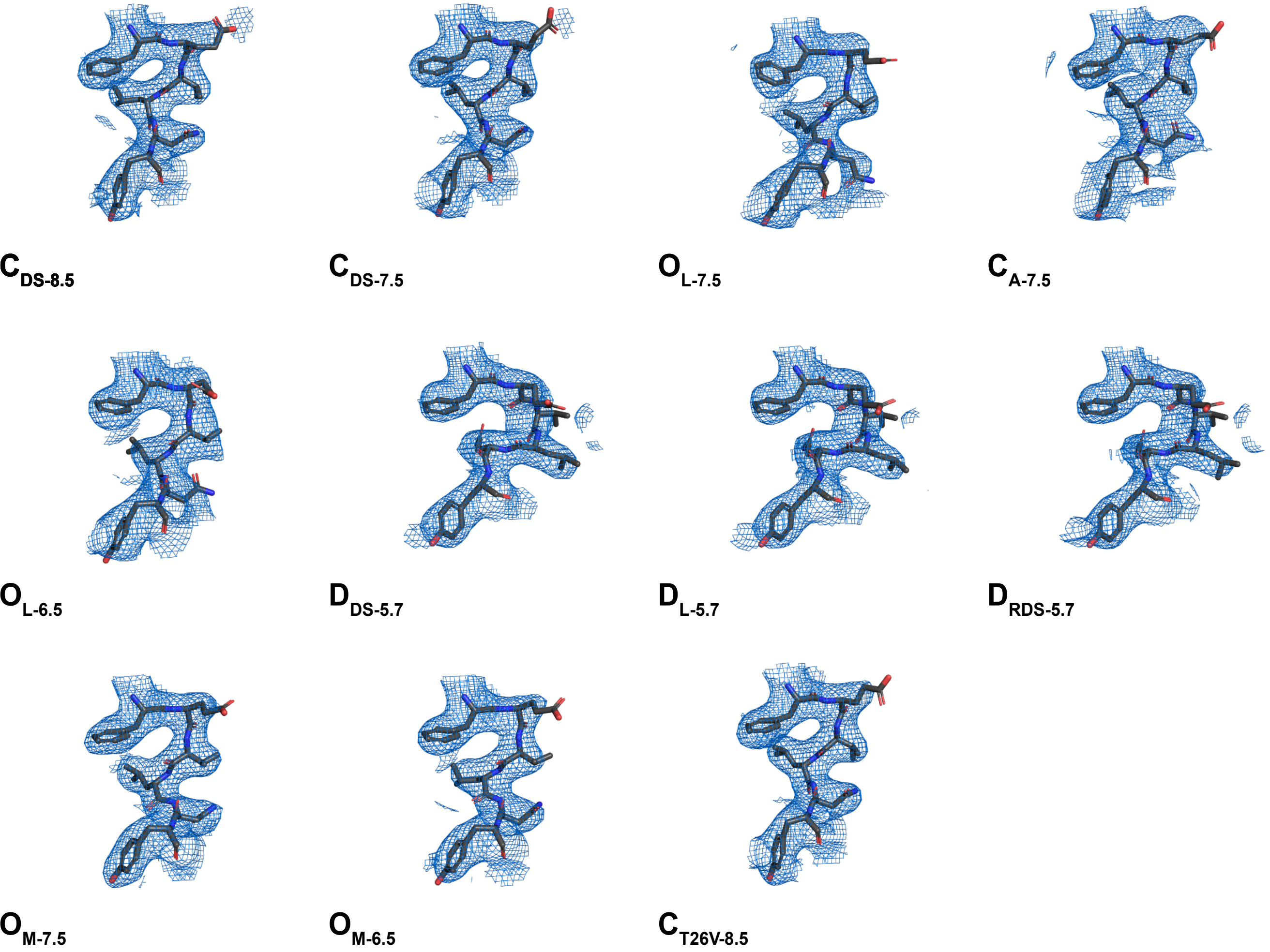

$C_{DS-8.5}$
$C_{DS-7.5}$
$O_{L-7.5}$
$C_{A-7.5}$
$O_{L-6.5}$
$D_{DS-5.7}$
$D_{L-5.7}$
$D_{RDS-5.7}$
$O_{M-7.5}$
$O_{M-6.5}$
$C_{T26V-8.5}$

Supplementary Data Figure 3: Cryo-EM map-model agreement

Sections of cryo-EM maps are overlaid onto corresponding stick models of featured regions of hASIC1a. Each segment from a specific map is contoured at the same level in each panel. Featured regions are: (a) TM1, (b) TM2, (c) α2 of the finger domain, (d) α4 of the thumb domain, (e) α5 of the thumb domain, (f) β11-β12 "clutch" linker.

Supplementary Table 1: Summary of electrophysiology data

**hASIC1a WT expressed in HEK293T* cells**	**Physiological buffer**	**Phosphate buffer**
pH response: hASIC1a WT expressed in HEK293T cells*		
pH_{50} activation	6.63 ± 0.02	7.16 ± 0.03
Desensitization kinetics (rate constants, S^{-1})	0.66 ± 0.10 (n=7)	0.63 ± 0.12 (n=13)
Permeability: hASIC1a WT expressed in HEK293T cells*		
Reversal potential at peak pH 6.5 (mV)		23.64 ± 5.47 (n=6)
Reversal potential at desensitization pH 6.5 (mV)		11.91 ± 7.60 (n=6)
Permeability: endogenous ASIC in HEK293 cells		
Reversal potential at peak pH 6.5 (mV)		35.79 ± 3.25 (n=4)
Reversal potential at desensitization pH 6.5 (mV)		12.40 ± 7.58 (n=4)
Desensitization recovery: hASIC1a WT expressed in HEK293T cells (whole cell)*		
short		1.05 ± 0.24 (n=9)
long		0.24 ± 0.35 (n=6)
recovery		1.03 ± 0.23 (n=6)
Desensitization recovery: hASIC1a WT expressed in HEK293T cells (excised patches)*		
short		0.71 ± 0.13 (n=5)
long		0.07 ± 0.11 (n=3)
recovery		0.74 ± 0.19 (n=3)
**hASIC1a WT expressed in *Xenopus* oocytes**	**ND96 (without Ca^{2+})**	**Phosphate buffer**
pH response		
pH_{50} activation	6.60 ± 0.03 (n=8)	6.87 ± 0.02 (n=11)
pH_{50} SSD	7.04 ± 0.02 (n=10)	7.33 ± 0.04 (n=7)
MitTx response relative to pH 6.0		
MitTx (20 nM) at pH 8.0	0.12 ± 0.10 (n=14)	0.58 ± 0.10 (n=14)
MitTx (20 nM) at pH 6.5		0.78 ± 0.19 (n=6)
MitTx (20 nM) after desensitization at pH 6.5		0.77 ± 0.12 (n=5)
Permeability		
Reversal potential at pH 6.5 (mV)	16.92 ± 11.50 (n=11)	18.00 ± 10.51 (n=13)
Reversal potential in MitTx (20nM) at pH 6.5 (mV)	-1.17 ± 6.74 (n=9)	-2.80 ± 18.11 (n=9)
**hASIC1a K105C expressed in *Xenopus* oocytes**	**Current**	**Fluorescence**
pH/MitTx response relative to pH 6.0		
pH 7.2	0.02 ± 0.02 (n=6)	0.60 ± 0.17 (n=6)
5 nM MitTx	0.07 ± 0.03 (n=4)	0.02 ± 0.02 (n=4)
20 nM MitTx	0.74 ± 0.19 (n=5)	0.39 ± 0.10 (n=5)
50 nM MitTx	0.77 ± 0.09 (n=9)	0.73 ± 0.23 (n=9)

Supplementary Table 2: PDB and EMDB codes of hASIC1a structures

Name	PDB	EMDB
$C_{DS\text{-}8.5}$	9E4A	EMD-47503
$C_{DS\text{-}7.5}$	9E4B	EMD-47504
$C_{A\text{-}7.5}$	9E4C	EMD-47505
$O_{L\text{-}7.5}$	9E4D	EMD-47506
$O_{L\text{-}6.5}$	9E4E	EMD-47507
$D_{DS\text{-}5.7}$	9E4F	EMD-47508
$D_{L\text{-}5.7}$	9E4G	EMD-47509
$D_{RDS\text{-}5.7}$	9E4H	EMD-47510
$O_{M\text{-}7.5}$	9E4I	EMD-47511
$O_{M\text{-}6.5}$	9E4J	EMD-47512
$C_{T26V\text{-}8.5}$	9E4K	EMD-47513

Supplementary Table 3: Cryo-EM data collection, refinement, and validation statistics

	$C_{DS-8.5}$ (EMDB-47503) (PDB 9E4A)	$C_{DS-7.5}$ (EMDB-47504) (PDB 9E4B)	$C_{A-7.5}$ (EMDB-47505) (PDB 9E4C)	$O_{L-7.5}$ (EMDB-47506) (PDB 9E4D)
**Data collection and processing**				
Magnification	105,000	105,000	105,000	105,000
Voltage (kV)	300	300	300	300
Electron exposure (e–/Å^2)	65	65	65	65
Defocus range (μm)	0.8-2.2	0.8-2.2	0.8-2.2	0.8-2.2
Pixel size (Å)	0.394	0.422	0.422	0.422
Symmetry imposed	C3	C3	C1	C1
Initial particle images (no.)	855,037	1,620,674	1,620,674	1,620,674
Final particle images (no.)	170,179	90,111	86,378	165,414
Map resolution (Å)	2.74	3.09	3.41	3.19
FSC threshold	0.143	0.143	0.143	0.143
**Refinement**				
Initial model used (PDB code)	6VTL	$C_{DS-8.5}$	$C_{T26V-8.5}$	$O_{L-6.5}$
Model resolution (Å)	2.7	3.1	3.4	3.2
FSC threshold	0.143	0.143	0.143	0.143
Map sharpening B factor (Å^2)	-93	-89	-79	-94
Model composition				
Non-hydrogen atoms	10842	10842	9729	9915
Protein residues	1353	1353	1212	1239
Ligands	0	0	0	0
B factors (Å^2)				
Protein	122.72	115.31	121.13	109.71
Ligand	n/a	n/a	n/a	n/a
R.m.s. deviations				
Bond lengths (Å)	0.002	0.001	0.002	0.003
Bond angles (°)	0.460	0.394	0.542	0.442
Validation				
MolProbity score	1.20	1.20	1.30	1.38
Clashscore	4.16	4.16	5.54	6.96
Poor rotamers (%)	0.00	0.00	0.00	0.00
Ramachandran plot				
Favored (%)	99.33	99.26	98.18	98.54
Allowed (%)	0.67	0.74	1.82	1.46
Disallowed (%)	0	0	0	0

	$O_{L\text{-}6.5}$ (EMDB-47507) (PDB 9E4E)	$D_{DS\text{-}5.7}$ (EMDB-47508) (PDB 9E4F)	$D_{L\text{-}5.7}$ (EMDB-47509) (PDB 9E4G)	$D_{RDS\text{-}5.7}$ (EMDB-47510) (PDB 9E4H)
**Data collection and processing**				
Magnification	105,000	105,000	105,000	105,000
Voltage (kV)	300	300	300	300
Electron exposure (e–/Å^2)	50	65	65	65
Defocus range (μm)	0.8-2.2	0.8-2.2	0.8-2.2	0.8-2.2
Pixel size (Å)	0.41275	0.4195	0.4195	0.4195
Symmetry imposed	C1	C3	C1	C3
Initial particle images (no.)	1,431,341	1,610,888	1,610,888	1,610,888
Final particle images (no.)	110,918	54,398	100,628	84,730
Map resolution (Å)	3.16	2.69	2.80	2.61
FSC threshold	0.143	0.143	0.143	0.143
**Refinement**				
Initial model used (PDB code)	$C_{DS\text{-}8.5}$	$C_{DS\text{-}8.5}$	$O_{L\text{-}6.5}$	$D_{L\text{-}5.7}$
Model resolution (Å)	3.1	2.7	2.8	2.6
FSC threshold	0.143	0.143	0.143	0.143
Map sharpening *B* factor (Å^2)	-81	-71	-66	-73
Model composition				
Non-hydrogen atoms	10158	10842	10024	10287
Protein residues	1266	1353	1251	1281
Ligands	0	0	0	0
B factors (Å^2)				
Protein	110.83	117.56	107.11	107.97
Ligand	n/a	n/a	n/a	n/a
R.m.s. deviations				
Bond lengths (Å)	0.002	0.002	0.002	0.002
Bond angles (°)	0.414	0.451	0.501	0.478
Validation				
MolProbity score	1.09	1.24	1.29	1.24
Clashscore	3.00	4.72	5.37	4.68
Poor rotamers (%)	0.00	0.00	0.00	0.00
Ramachandran plot				
Favored (%)	98.97	98.44	98.15	99.06
Allowed (%)	1.03	1.56	1.85	0.94
Disallowed (%)	0	0	0	0

	$O_{M-7.5}$ (EMDB-47511) (PDB 9E4I)	$O_{M-6.5}$ (EMDB-47512) (PDB 9E4J)	$C_{T26V-8.5}$ (EMDB-47513) (PDB 9E4K)
**Data collection and processing**			
Magnification	105,000	105,000	105,000
Voltage (kV)	300	300	300
Electron exposure (e–/Å^2)	50	65	65
Defocus range (μm)	0.8-2.2	0.8-2.2	0.8-2.2
Pixel size (Å)	0.41275	0.422	0.4135
Symmetry imposed	C1	C1	C1
Initial particle images (no.)	1,953,574	778,825	2,759,695
Final particle images (no.)	541,317	100,508	102,014
Map resolution (Å)	2.33	2.87	2.77
FSC threshold	0.143	0.143	0.143
**Refinement**			
Initial model used (PDB code)	$O_{L-6.5}$; 4NTW	$O_{M-7.5}$	$C_{DS-7.5}$
Model resolution (Å)	2.3	2.9	2.8
FSC threshold	0.143	0.143	0.143
Map sharpening *B* factor (Å^2)	-87	-64	-60
Model composition			
Non-hydrogen atoms	14241	14285	9915
Protein residues	1773	1778	1239
Ligands	0	0	0
B factors (Å^2)			
Protein	111.44	136.75	110.98
Ligand	n/a	n/a	n/a
R.m.s. deviations			
Bond lengths (Å)	0.003	0.002	0.002
Bond angles (°)	0.451	0.428	0.420
Validation			
MolProbity score	1.15	1.19	1.17
Clashscore	3.59	4.05	3.84
Poor rotamers (%)	0.00	0.00	0.00
Ramachandran plot			
Favored (%)	99.09	98.80	99.03
Allowed (%)	0.91	1.20	0.97
Disallowed (%)	0	0	0

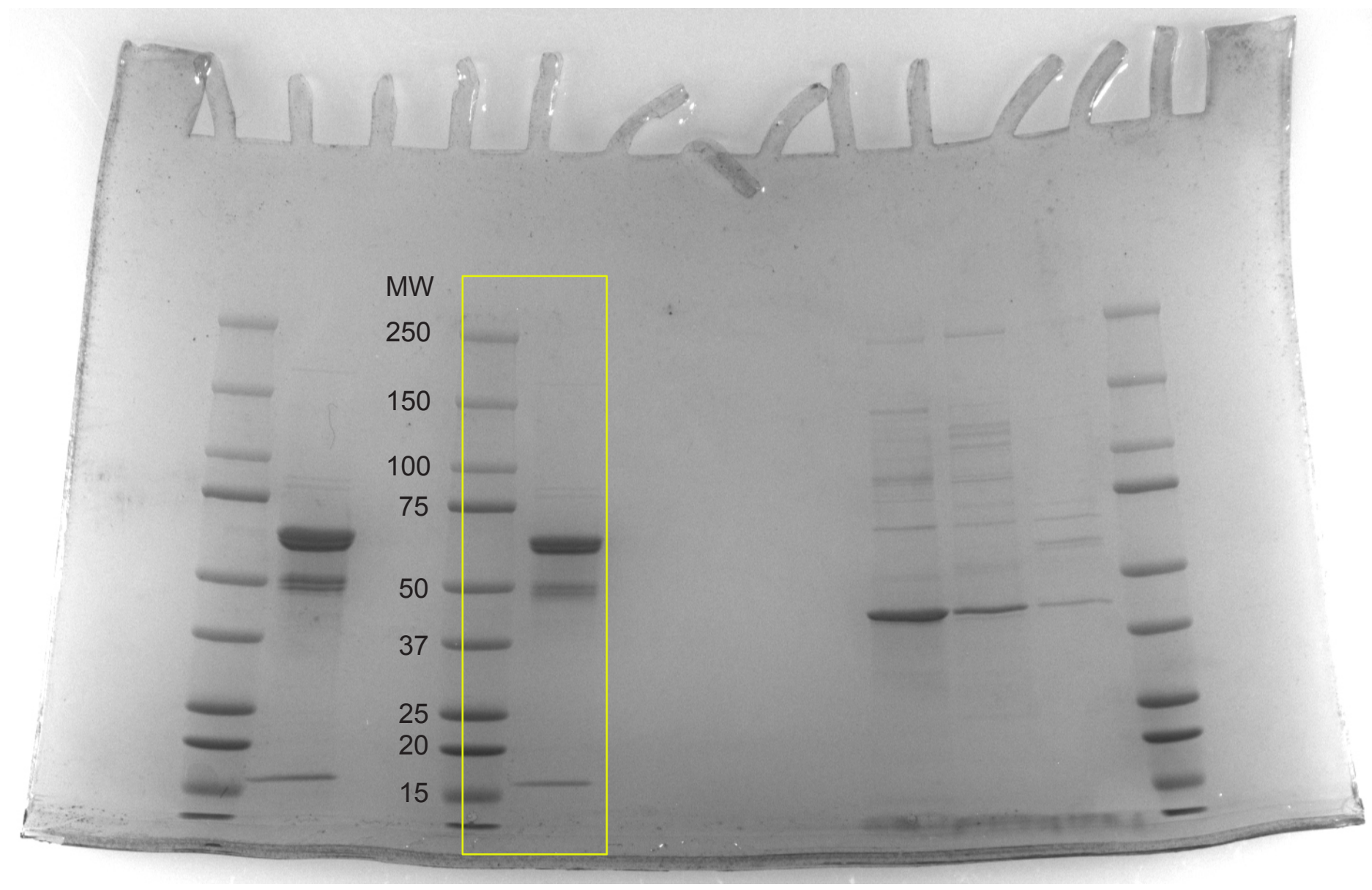

Raw data for uncropped Coomassie-stained gel.

Original Coomassie stained gel (see Supplementary Fig. 1d). Yellow box indicate how the gel was cropped.
