# Supplementary material for: Conformational plasticity of human acid-sensing ion channel 1a
Source: Nat Struct Mol Biol. 2026 Jul 16;33(8):1171–82. doi: 10.1038/s41594-026-01845-0 (PMC13468109; doi:10.1038/s41594-026-01845-0)
Supplement: Supplementary file 3 [file 41594_2026_1845_MOESM3_ESM.pdf]

nature portfolio

Peer Review File

Conformational plasticity of human acid-sensing ion channel 1a

Corresponding Author: Dr Isabelle Baconguis

Version 0:

Decision Letter:

4th Feb 2025

Dear Dr. Baconguis,

Thank you for submitting your manuscript "Conformational plasticity of human acid-sensing ion channel 1a". I apologize for the delay in this decision. I'm writing to let you know that we have decided to send your manuscript for peer review.

I am re-opening the manuscript submission link for you to resubmit your manuscript with all the associated files needed for the peer review process directly to our system, at your convenience. Please see below for details regarding the required materials. Please follow the link at the bottom of this email to upload the documents.

We want to ensure that the methods and statistics reporting in our papers are of the highest quality. To that end, we ask authors to fill out a Reporting Summary that collects information on experimental design and reagents, as well as an editorial Policy Checklist, which confirms compliance with our editorial policies, including the declaration of Competing Interests. If your paper includes ChIP-seq, flow cytometry or MRI data, we ask you take special care to complete those sections of the Reporting Summary as this data will aid greatly in the review of your manuscript.

These documents can be found by following the links below:

Reporting Summary:
https://www.nature.com/documents/nr-reporting-summary.pdf

Editorial Policy Checklist: https://www.nature.com/documents/nr-editorial-policy-checklist.pdf

Please be aware of our guidelines on digital image standards.

IMPORTANT

In order for us to proceed with the peer review process of your manuscript, we require you to provide accession numbers and reviewer tokens to access sequencing data sets. Please add this information to your manuscript file.

Please note we require official wwPDB validation reports for newly described atomic structures, as noted in the policy checklist. We also request that authors provide cryo-EM maps, half-maps and models, to help the reviewers in assessing the work. We recommend the use of figshare integration into our systems, which allows for provision of anonymous access links for the referees (https://www.springernature.com/gp/authors/research-data/figshare-integration). Alternatively, please upload .zip folders directly with the submission. To ensure the ease of reviewer access to the data, please specify in the Data Availability section, where the files can be found (provide a figshare link or direct the reader to the manuscript files).

Additionally, I would like to kindly request that you provide the code used to analyse the data to the reviewers, if used. In order for the reviewers to evaluate the work adequately they must be able to test the software/review the code themselves. If you have not yet provided the software, we therefore request that you provide a single compressed zip file containing the software with a readme.txt file or other user manual containing complete instructions for installing and running the software. If appropriate, please also provide example data and expected output. Sufficient material should be provided for referees to directly test the performance of the software/algorithm. If the software and materials are small enough to fit in a single compressed zip file less than 6MB in size, you may email this file directly to me. If the zip file is between 6 MB and 200 MB you may upload it to our file transfer site. If necessary, a second zip file up to 200 MB in size can be used to supply the example data. Please let me know if you need to use this option and I'll send you further details. Alternatively, you can also

upload the code to GitHub and provide us with the link.

Please also fill out and return to me the code and software submission checklist that will be made available to editors and reviewers during manuscript assessment. Please note that this form is a dynamic 'smart pdf' and must therefore be downloaded and completed in Adobe Reader, instead of opening it in a web browser.

https://www.nature.com/documents/nr-software-policy.pdf

Please use the link below to submit the files. **Please also remember to move forward all other files associated with this version of the paper.**

Link Redacted

Note: This URL links to your confidential home page and associated information about manuscripts you may have submitted, or that you are reviewing for us. If you wish to forward this email to co-authors, please delete the link to your homepage.

Sincerely,

Katarzyna Ciazynska, PhD
(she/her)
Senior Editor
Nature Structural & Molecular Biology
https://orcid.org/0000-0002-9899-2428

Version 1:

Decision Letter:

11th Apr 2025

Dear Dr. Baconguis,

Thank you again for submitting your manuscript "Conformational plasticity of human acid-sensing ion channel 1a". I apologize for the delay in responding, which resulted from the difficulty in obtaining suitable referee reports. Nevertheless, we now have comments (below) from the 3 reviewers who evaluated your paper. In light of those reports, we remain interested in your study and would like to see your response to the comments of the referees, in the form of a revised manuscript.

You will see that while reviewers appreciate the results, they raise several concerns which will need to be addressed in a revision. Specifically, we ask that you strengthen the functional aspects of the manuscript, in line with comments of reviewers #2 and #3. This pertains both to the disconnect between functional and structural work, as well as the concerns about the support for the function of the re-entrant loop and the role of desensitized conformations more generally.

Please be sure to address/respond to all concerns of the referees in full in a point-by-point response and highlight all changes in the revised manuscript text file. If you have comments that are intended for editors only, please include those in a separate cover letter.

We are committed to providing a fair and constructive peer-review process. Do not hesitate to contact us if there are specific requests from the reviewers that you believe are technically impossible or unlikely to yield a meaningful outcome.

We expect to see your revised manuscript within 3-5 months. If you cannot send it within this time, please contact us to discuss an extension; we would still consider your revision, provided that no similar work has been accepted for publication at NSMB or published elsewhere.

As you already know, we put great emphasis on ensuring that the methods and statistics reported in our papers are correct and accurate. As such, if there are any changes that should be reported, please submit an updated version of the Reporting

Summary along with your revision.

Please follow the links below to download these files:

Reporting Summary:
https://www.nature.com/documents/nr-reporting-summary.pdf

Please note that the form is a dynamic 'smart pdf' and must therefore be downloaded and completed in Adobe Reader.

When submitting the revised version of your manuscript, please pay close attention to our href="https://www.nature.com/nature-portfolio/editorial-policies/image-integrity">Digital Image Integrity Guidelines. and to the following points below:

-- that unprocessed scans are clearly labelled and match the gels and western blots presented in figures.
-- that control panels for gels and western blots are appropriately described as loading on sample processing controls
-- all images in the paper are checked for duplication of panels and for splicing of gel lanes.

EXTENDED DATA FIGURES

When re-submitting your manuscript, please ensure that any supplementary figures and tables that are crucial to the manuscript's conclusions are converted into Extended Data figures and tables to increase visibility of these data. Extended Data figures and tables are online-only (present in the online PDF and full-text HTML versions of the paper), peer-reviewed display items that provide essential background to the article but are not included in the main article due to space constraints. A maximum of ten Extended Data display items (figures and tables) is permitted.

Finally, please ensure that you retain unprocessed data and metadata files after publication, ideally archiving data in perpetuity, as these may be requested during the peer review and production process or after publication if any issues arise.

If there are additional or modified structures presented in the final revision, please submit the corresponding PDB validation reports.

Please note that all key data shown in the main figures as cropped gels or blots should be presented in uncropped form, with molecular weight markers. These data can be aggregated into a single supplementary figure item. While these data can be displayed in a relatively informal style, they must refer back to the relevant figures. These data should be submitted with the final revision, as source data, prior to acceptance, but you may want to start putting it together at this point.

SOURCE DATA: we urge authors to provide, in tabular form, the data underlying the graphical representations used in figures. This is to further increase transparency in data reporting, as detailed in this editorial (http://www.nature.com/nsmb/journal/v22/n10/full/nsmb.3110.html). Spreadsheets can be submitted in excel format. Only one (1) file per figure is permitted; thus, for multi-paneled figures, the source data for each panel should be clearly labeled in the Excel file; alternately the data can be provided as multiple, clearly labeled sheets in an Excel file. When submitting files, the title field should indicate which figure the source data pertains to. We encourage our authors to provide source data at the revision stage, so that they are part of the peer-review process.

Data availability: this journal strongly supports public availability of data. All data used in accepted papers should be available via a public data repository, or alternatively, as Supplementary Information. If data can only be shared on request, please explain why in your Data Availability Statement, and also in the correspondence with your editor. Please note that for some data types, deposition in a public repository is mandatory - more information on our data deposition policies and available repositories can be found below:
https://www.nature.com/nature-research/editorial-policies/reporting-standards#availability-of-data

We require deposition of coordinates (and, in the case of crystal structures, structure factors) into the Protein Data Bank with the designation of immediate release upon publication (HPUB). Electron microscopy-derived density maps and coordinate data must be deposited in EMDB and released upon publication. Deposition and immediate release of NMR chemical shift assignments are highly encouraged. Deposition of deep sequencing and microarray data is mandatory, and the datasets must be released prior to or upon publication. To avoid delays in publication, dataset accession numbers must be supplied with the final accepted manuscript and appropriate release dates must be indicated at the galley proof stage.

While we encourage the use of color in preparing figures, please note that this will incur a charge to partially defray the cost of printing. Information about color charges can be found at http://www.nature.com/nsmb/authors/submit/index.html#costs

Nature Structural & Molecular Biology is committed to improving transparency in authorship. As part of our efforts in this direction, we are now requesting that all authors identified as 'corresponding author' on published papers create and link their Open Researcher and Contributor Identifier (ORCID) with their account on the Manuscript Tracking System (MTS), prior to acceptance. This applies to primary research papers only. ORCID helps the scientific community achieve unambiguous attribution of all scholarly contributions. You can create and link your ORCID from the home page of the MTS by clicking on 'Modify my Springer Nature account'. For more information please visit please visit www.springernature.com/orcid.

Please use the link below to submit your revised manuscript and related files:

Link Redacted

Note: This URL links to your confidential home page and associated information about manuscripts you may have submitted, or that you are reviewing for us. If you wish to forward this email to co-authors, please delete the link to your homepage.

We look forward to seeing the revised manuscript and thank you for the opportunity to review your work.

Sincerely,

Katarzyna Ciazynska, PhD
(she/her)
Senior Editor
Nature Structural & Molecular Biology
https://orcid.org/0000-0002-9899-2428

Referee expertise:

Referee #1: ASIC, neurobiology

Referee #2: ASIC, cryo-EM

Referee #3: cryo-EM, DEG/ENaC

Reviewers' Comments:

Reviewer #1 (Remarks to the Author):

The manuscript by Cahill et. al. entitled "Conformational plasticity of human acid-sensing ion channel 1a" reports for the first time a conformational library of human homotrimeric ASIC1a channels across a range of pH values revealing a number of conformational changes related to the resting closed, activated open, and desensitized closed states. A technical advance that seems to have been critical for this work is the switch to a phosphate NaCl buffer system.
Overall, this is important work and in my view will add valuable information to the field; increasing possibilities for identifying, designing, and testing new channel modulators.
Can the authors speculate why the switch to phosphate buffer shifted the activation profile of the channels in the alkaline direction?
Can the authors comment on whether any of the new observations from these structures provide specific understandings about channel access by any of the other known peptide modulators or even points of access for new chemical modulators targeting the different states?
Also, since the majority of ASICs in brain appear to be comprised of heterotrimeric channels, can the authors comment about how any structural observations from this work might apply to or differ from heterotrimeric channels comprised of ASIC1a plus ASIC2a or 2b for example?

Reviewer #2 (Remarks to the Author):

Acid sensing ion channels (ASICs) are ubiquitously expressed in the nervous system of various animals. The channels are activated by H+ upon acidification of extracellular milieu, and once activated they contribute to excitatory Na+ current. A number of ASIC structures have been solved to date at different pH, revealing the architecture of these proteins, and conformations in resting and activating pH. However, due to rapid ASIC desensitization, the structures obtained in the activating conditions represent inactivated channels, and the open conformation of ASICs has remained elusive.
The authors solve 8 new structures of human ASIC1a at different pH using the same buffering system in resting, putatively activated and desensitized conformations, which reveal a high structural plasticity of the pore helices and point towards a novel activation mechanism, where in resting conditions the pore helices are in the 'helix-swapped' conformation, in the open channel the helices are linearized, and in desensitized conformation the helices assume the swapped conformation again. Further, the authors solve the structures in presence of the activating toxin (MitTx) and of the activating mutant T26V, which further support the proposed activation mechanism. The structures are complemented by functional characterization of ASIC1a channels.
This is an exciting study suggesting that ASIC1a might be activated differently from what was suggested before (TMD helices remain swapped in open channel), and that there might be differences in activation mechanisms in broader DEG/ENaC channel superfamily (i.e. ASICs compared to BASICs or FaNaCs). The authors make compelling arguments to support their hypothesis, and I very much appreciate the "Potential Methodological Constraints" section of the discussion. That being said, I do think that the authors could exercise more caution interpreting their data, as other explanations are

possible. Below are my comments.

Essential revisions:
1. I do not think there is a clear equivalence between structural and functional data, as the authors point out themselves – ASIC1a desensitizes rapidly in electrophysiological experiments, but not during cryo-EM sample preparation at lower pH, the concentration of phosphate buffer used in recordings and protein purification has a 10x difference, etc. ASIC1a could transition through different conformations of TMD helices as it activates or desensitizes, or the linear helix conformations could be artifactual due to the lack of membrane environment etc. Perhaps a way to address this would be additional functional experiments (fluorimetry) probing the changes in the reentrant loop conformation. Additionally, the authors could do cysteine accessibility experiments with the residues in the lower pore region – the positions of some of them change between swapped and linear helix conformations.
2. OL and OM conformations are indeed very similar, and it is unclear how selectivity can be achieved in one but not the other. The authors suggest that the changes might be minor and not apparent at this resolution. Alternatively, the structures with linear helices might represent non-selective channels. This would also be consistent with T26V mutant (linear helices in the structure) having lower Na+ selectivity (Sheikh et al., 2021). The authors might consider adding "and reduces ion selectivity" to the line 169. The authors should also discuss the conformation of the selectivity filter in their resting / 'open' / desensitized structures.
3. Both OL and OM maps have prominent non-protein densities partially inserting into the pore (particularly OL map), which were not taken into account when calculating pore diameter. I am struggling with the idea that this would not interfere with ion conduction. Current Figure 4a is misleading, as it does not show just how much these densities are inside the pore. The authors should describe these densities, along with implications for ion conduction, and prepare a new figure clearly demonstrating them, with the surrounding protein density at the same contour.
4. The manuscript would benefit from a more extensive discussion on the role of GAS and HG motives for channel gating / ion selectivity, in the context of the newly solved structures and the proposed activation mechanism with linear helices. The GAS motif refolding completely changes the lower pore profile. Additionally, the HG motif was shown important for ion selectivity (Sheikh et al., 2021) – how is this achieved if in the linear TM conformation the reentrant loop is not lining the pore anymore?
5. Please include data processing workflows for every structure, this is the standard in the field.

Presentation:
1. Figure 4 and Extended Data Figure 5 – the pore profiles are not really informative, the authors could for example only show the pore regions of their structures next to calculated pore diameter plots, and label the residues forming constrictions, which are mentioned in the text.

Reviewer #3 (Remarks to the Author):

The manuscript by Cahill et al. presents an extensive cryo-EM study of human ASIC1a, detailing six distinct conformations captured under various pH conditions as well as with pharmacological modulation via MitTx and a T26V mutation. The work provides valuable insights into the structural transitions associated with channel activation and desensitization. The overall analysis is thorough, and the manuscript is clearly written. However, several points need to be addressed to enhance the clarity and rigor of the study:

1. The identification of three distinct desensitized conformations (DDS, DL, DRDS) is intriguing, but their functional relevance remains speculative. Electrophysiological data—such as recovery rates from desensitization and ion selectivity differences between states—are essential to link these structures to specific functional phenotypes. For example, does DRDS represent a metastable intermediate during desensitization, or is it a unique nonconducting state? Additional experiments (e.g., single-channel recordings or kinetic analyses) could help clarify these distinctions.
2. The T26V mutation is reported to increase leak currents, which is attributed to the destabilization of the pre-TM1 "re-entrant loop." However, the manuscript lacks direct evidence (such as detailed hydrogen-bonding interactions in CDS versus CT26V or single-channel recordings) to support this claim. The authors should consider molecular dynamics simulations or additional mutagenesis experiments (e.g., comparisons of T26A vs. T26C) to validate the specific role of Thr26 in stabilizing the closed state.
3. The discussion briefly touches on ASIC1a's roles in stroke and pain but does not fully explore the therapeutic implications of the observed conformational diversity. For instance, could the stabilization of specific desensitized states (such as DDS) potentially mitigate acidosis-induced neuronal damage? Furthermore, how might the insights gained from toxin-MitTx interactions inform the design of non-proton agonists? A deeper discussion in this regard would enhance the manuscript's broader impact.
4. The local resolution limitations in the TMD region (Extended Data Figs. 2d-e, 4a-b) raise concerns about the accuracy of side-chain placements, particularly for key pore-lining residues such as Leu441 and Asp434. A dedicated section discussing potential model uncertainties and how these might affect the mechanistic interpretations would strengthen the manuscript.

Version 2:

Decision Letter:

Our ref: NSMB-A50406B

6th Feb 2026

Dear Dr. Baconguis,

Thank you for submitting your revised manuscript "Conformational plasticity of human acid-sensing ion channel 1a" (NSMB-A50406B). It has now been seen by the original referees #1 and #3 and their comments are below. Unfortunately, reviewer #2 was unavailable to assess the revision, but considering overlaps in their concerns, reviewer #3 was asked to assess the revision in response to comments of both reviewers. The reviewers find that the paper has improved in revision, and therefore we'll be happy in principle to publish it in Nature Structural & Molecular Biology, pending minor revisions to satisfy the referees' final requests and to comply with our editorial and formatting guidelines.

We are now performing detailed checks on your paper and will send you a checklist detailing our editorial and formatting requirements in about 2-3 weeks. Please do not upload the final materials and make any revisions until you receive this additional information from us.

To facilitate our work at this stage, it is important that we have a copy of the main text as a word file. If you could please send along a word version of this file as soon as possible, we would greatly appreciate it; please make sure to copy the NSMB account (cc'ed above).

Thank you again for your interest in Nature Structural & Molecular Biology Please do not hesitate to contact me if you have any questions.

Sincerely,

Katarzyna Ciazynska, PhD
(she/her)
Senior Editor
Nature Structural & Molecular Biology
https://orcid.org/0000-0002-9899-2428

Reviewer #1 (Remarks to the Author):

My queries about the earlier version of this manuscript have now been addressed. I believe this paper is appropriate for publication in Nature Structural and Molecular Biology.

Reviewer #3 (Remarks to the Author):

The authors have addressed all my concerns, and the manuscript is acceptable for publication.

Version 3:

Decision Letter:

19th Jun 2026

Dear Dr. Baconguis,

We are now happy to accept your revised paper "Conformational plasticity of human acid-sensing ion channel 1a" for publication as an Article in Nature Structural & Molecular Biology.

Acceptance is conditional on the manuscript's not being published elsewhere and on there being no announcement of this work to the newspapers, magazines, radio or television until the publication date in Nature Structural & Molecular Biology.

Over the next few weeks, your paper will be copyedited to ensure that it conforms to Nature Structural & Molecular Biology style. Once your paper is typeset, you will receive an email with a link to choose the appropriate publishing options for your paper and our Author Services team will be in touch regarding any additional information that may be required.

After the grant of rights is completed, you will receive a link to your electronic proof via email with a request to make any corrections within 48 hours. If, when you receive your proof, you cannot meet this deadline, please inform us at rjsproduction@springernature.com immediately.

You will not receive your proofs until the publishing agreement has been received through our system.

Due to the importance of these deadlines, we ask that you please let us know now whether you will be difficult to contact over the next month. If this is the case, we ask you provide us with the contact information (email, phone and fax) of someone who will be able to check the proofs on your behalf, and who will be available to address any last-minute problems.

To assist our authors in disseminating their research to the broader community, our SharedIt initiative provides all co-authors with the ability to generate a unique shareable link that will allow anyone (with or without a subscription) to read the published article. Recipients of the link with a subscription will also be able to download and print the PDF.

As soon as your article is published, you can generate your shareable link by entering the DOI of your article here: http://authors.springernature.com/share. Corresponding authors will also receive an automated email with the shareable link

Note the policy of the journal on data deposition: http://www.nature.com/authors/policies/availability.html.

Your paper will be published online soon after we receive proof corrections and will appear in print in the next available issue. You can find out your date of online publication by contacting the production team shortly after sending your proof corrections.

You may wish to make your media relations office aware of your accepted publication, in case they consider it appropriate to organize some internal or external publicity. Once your paper has been scheduled you will receive an email confirming the publication details. This is normally 3-4 working days in advance of publication. If you need additional notice of the date and time of publication, please let the production team know when you receive the proof of your article to ensure there is sufficient time to coordinate. Further information on our embargo policies can be found here: https://www.nature.com/authors/policies/embargo.html

You can now use a single sign-on for all your accounts, view the status of all your manuscript submissions and reviews, access usage statistics for your published articles and download a record of your refereeing activity for the Nature journals.

If you have not already done so, we strongly recommend that you upload the step-by-step protocols used in this manuscript to the Protocol Exchange. Protocol Exchange is an open online resource that allows researchers to share their detailed experimental know-how. All uploaded protocols are made freely available, assigned DOIs for ease of citation and fully searchable through nature.com. Protocols can be linked to any publications in which they are used and will be linked to from your article. You can also establish a dedicated page to collect all your lab Protocols. By uploading your Protocols to Protocol Exchange, you are enabling researchers to more readily reproduce or adapt the methodology you use, as well as increasing the visibility of your protocols and papers. Upload your Protocols at www.nature.com/protocolexchange/. Further information can be found at www.nature.com/protocolexchange/about.

An online order form for reprints of your paper is available at https://www.nature.com/reprints/author-reprints.html. Please let your coauthors and your institutions' public affairs office know that they are also welcome to order reprints by this method.

Authors may need to take specific actions to achieve compliance with funder and institutional open access mandates. If your research is supported by a funder that requires immediate open access (e.g. according to Plan S principles or the NIH public access policy) then you should select the gold OA route, and we will direct you to the compliant route where possible. Because authors warrant under our subscription licensing terms that they haven't committed to licensing any version of their article under a licence inconsistent with the terms of our agreement – including the applicable embargo period – publication under the subscription model isn't suitable for authors whose funders require no embargo.

In approximately 10 business days you will receive an email with a link to choose the appropriate publishing options for your paper and our Author Services team will be in touch regarding any additional information that may be required.

You will not receive your proofs until the publishing agreement has been received through our system.

If you have any questions about our publishing options, costs, Open Access requirements, or our legal forms, please contact ASJournals@springernature.com

Sincerely,

Katarzyna Ciazynska, PhD
(she/her)
Senior Editor
Nature Structural & Molecular Biology
https://orcid.org/0000-0002-9899-2428

Click here if you would like to recommend Nature Structural & Molecular Biology to your librarian:
http://www.nature.com/subscriptions/recommend.html#forms

Open Access This Peer Review File is licensed under a Creative Commons Attribution 4.0 International License, which permits use, sharing, adaptation, distribution and reproduction in any medium or format, as long as you give appropriate credit to the original author(s) and the source, provide a link to the Creative Commons license, and indicate if changes were made.
In cases where reviewers are anonymous, credit should be given to 'Anonymous Referee' and the source.
The images or other third party material in this Peer Review File are included in the article's Creative Commons license, unless indicated otherwise in a credit line to the material. If material is not included in the article's Creative Commons license and your intended use is not permitted by statutory regulation or exceeds the permitted use, you will need to obtain permission directly from the copyright holder.
To view a copy of this license, visit https://creativecommons.org/licenses/by/4.0/

We are grateful to the reviewers and editor for their insightful feedback, which has substantially improved both the clarity and impact of our manuscript. We have addressed every comment in detail, performing new analyses and revising the text where appropriate. Our point-by-point responses are provided below and are highlighted in blue. These responses also specify the corresponding revisions to the manuscript, including section and line numbers for ease of review. For clarity, comments and responses are organized by reviewer.

Reviewer #1 (Remarks to the Author):

The manuscript by Cahill et. al. entitled "Conformational plasticity of human acid-sensing ion channel 1a" reports for the first time a conformational library of human homotrimeric ASIC1a channels across a range of pH values revealing a number of conformational changes related to the resting closed, activated open, and desensitized closed states. A technical advance that seems to have been critical for this work is the switch to a phosphate NaCl buffer system.
Overall, this is important work and in my view will add valuable information to the field; increasing possibilities for identifying, designing, and testing new channel modulators.

1) Can the authors speculate why the switch to phosphate buffer shifted the activation profile of the channels in the alkaline direction?

We thank the reviewer for this insightful question. We speculate that the shift in the activation profile observed upon switching to phosphate buffer is primarily due to calcium sequestration or precipitation. Phosphate can form insoluble complexes with divalent cations such as Ca^{2+}, effectively reducing the free Ca^{2+} concentration in solution. Because extracellular Ca^{2+} is known to stabilize the closed state of ASIC1a, its removal lowers the energetic barrier for channel activation, thereby promoting opening at more alkaline pH values. Thus, the observed shift likely reflects the combined effects of phosphate buffering and diminished ambient Ca^{2+} levels on channel gating. We have added a sentence to address this point in lines 85-86: "This alkaline shift is likely due to calcium sequestration or precipitation by phosphate, which reduces ambient free Ca^{2+} and thereby favors channel activation[23,24]."

2) Can the authors comment on whether any of the new observations from these structures provide specific understandings about channel access by any of the other known peptide modulators or even points of access for new chemical modulators targeting the different states?

We appreciate the reviewer's thoughtful comment. The extracellular domains (ECDs) in our newly resolved structures are largely consistent with those reported in prior studies, with no major rearrangements that would immediately suggest new access points for peptide or chemical modulators. However, a key advance of our study is the observation, at pH 7.5, of multiple, distinct ECD conformations coexisting within the same dataset. To our knowledge, this represents the first structural evidence that ASIC1a can adopt a range of ECD conformations under a single pH condition. This

conformational heterogeneity suggests that ASIC1a exists in a dynamic equilibrium between structural states, which may be a fundamental feature of its regulation. Importantly, modulators like PcTx1 bind in a state-dependent manner and alter steady-state desensitization. Our findings therefore provide a structural basis for how such modulators can act selectively by exploiting pre-existing conformational ensembles. Given that PcTx1 has served as a foundation for designing new ASIC-targeting molecules, this insight into the structural plasticity of the ECD may have broad implications for the development of next-generation modulators. We now highlight this point in the revised discussion section lines 357-362, which states: “Such heterogeneity could explain how state-dependent modulators like PcTx1, which bind selectively and alter steady-state desensitization, exert their effects[44]. Our structural observations provide a mechanistic basis for how such modulators may act by stabilizing specific pre-existing conformations. Given that PcTx1 has served as a foundation for designing new ASIC-targeting compounds[45,46], our findings may inform future efforts to exploit this intrinsic conformational plasticity for therapeutic development.”

3) Also, since the majority of ASICs in brain appear to be comprised of heterotrimeric channels, can the authors comment about how any structural observations from this work might apply to or differ from heterotrimeric channels comprised of ASIC1a plus ASIC2a or 2b for example?

We agree this is an important consideration, although direct structural comparisons are challenging because our current data are limited to homotrimeric hASIC1a. That said, we would expect certain key features of the gating mechanism, particularly those involving the highly conserved HG motif in pre-M1 (including T26) and the GAS belt in TM2, to be preserved in heterotrimeric channels such as ASIC1a/2a or ASIC1a/2b. Their conservation suggests that similar structural transitions may underlie gating in heteromeric assemblies. However, given that heteromeric channels exhibit distinct functional properties, such as altered proton sensitivities, compared to homomeric ASIC1a, it is possible that the distribution and relative occupancy of different conformational states may differ from what we observe in this current study. We emphasize that this is speculative, and structural and functional characterization of heteromeric channels will be essential to determine the extent to which these conformations are conserved or diverge in a mixed-subunit context. We added a section in the Discussion section under the subheading Outlook lines 476-485 to discuss this topic: “Our study focuses on homotrimeric hASIC1a; it is important to consider how these findings might relate to heteromeric ASIC channels, which also exist in the brain. Key elements implicated in gating, such as the conserved HG motif and Thr26 of pre-TM1 and the GAS motif of TM2, are present across ASIC subtypes, suggesting that similar conformational transitions may underlie gating in heteromeric assemblies such as ASIC1a/1b or ASIC1a/2b. However, heteromeric channels exhibit distinct functional properties, including different proton sensitivities, compared to homomeric ASIC1a. We speculate that the distribution and relative occupancy of conformational states may differ from those captured in this study. Structural and functional characterization of heteromeric ASICs will be critical to determine the extent to which these conformations are conserved or diverge across physiologically relevant assemblies.”

Reviewer #2 (Remarks to the Author):

Acid sensing ion channels (ASICs) are ubiquitously expressed in the nervous system of various animals. The channels are activated by H+ upon acidification of extracellular milieu, and once activated they contribute to excitatory Na+ current. A number of ASIC structures have been solved to date at different pH, revealing the architecture of these proteins, and conformations in resting and activating pH. However, due to rapid ASIC desensitization, the structures obtained in the activating conditions represent inactivated channels, and the open conformation of ASICs has remained elusive.
The authors solve 8 new structures of human ASIC1a at different pH using the same buffering system in resting, putatively activated and desensitized conformations, which reveal a high structural plasticity of the pore helices and point towards a novel activation mechanism, where in resting conditions the pore helices are in the 'helix-swapped' conformation, in the open channel the helices are linearized, and in desensitized conformation the helices assume the swapped conformation again. Further, the authors solve the structures in presence of the activating toxin (MitTx) and of the activating mutant T26V, which further support the proposed activation mechanism. The structures are complemented by functional characterization of ASIC1a channels.
This is an exciting study suggesting that ASIC1a might be activated differently from what was suggested before (TMD helices remain swapped in open channel), and that there might be differences in activation mechanisms in broader DEG/ENaC channel superfamily (i.e. ASICs compared to BASICs or FaNaCs). The authors make compelling arguments to support their hypothesis, and I very much appreciate the "Potential Methodological Constraints" section of the discussion. That being said, I do think that the authors could exercise more caution interpreting their data, as other explanations are possible. Below are my comments.

Essential revisions:
1. I do not think there is a clear equivalence between structural and functional data, as the authors point out themselves – ASIC1a desensitizes rapidly in electrophysiological experiments, but not during cryo-EM sample preparation at lower pH, the concentration of phosphate buffer used in recordings and protein purification has a 10x difference, etc. ASIC1a could transition through different conformations of TMD helices as it activates or desensitizes, or the linear helix conformations could be artifactual due to the lack of membrane environment etc. Perhaps a way to address this would be additional functional experiments (fluorimetry) probing the changes in the reentrant loop conformation. Additionally, the authors could do cysteine accessibility experiments with the residues in the lower pore region – the positions of some of them change between swapped and linear helix conformations.

We appreciate the reviewer's interest in further probing the relationship between the observed structures and functional states. We agree that correlating structural and functional data for ASIC1a is important. We respectfully note that the proposed experimental approaches, such as fluorometry, present significant technical challenges in the context of rapidly desensitizing channels like hASIC1a. With fluorometry, while

such an approach could potentially indicate movement, it would not provide insight into specific structural rearrangements such as domain-swapping versus linear helical conformations, nor would it offer the temporal resolution needed to capture transient gating transitions.

We note that cysteine accessibility experiments have already been elegantly applied to ASIC1a (Pfister et al., which we cited in our manuscript), particularly to probe residues in the re-entrant loop and TM1. This study showed that while many substitutions disrupted channel function, one residue (Arg43 in TM1) was especially striking. Arg43 was shown to exhibit robust Cd^{2+} accessibility from both extracellular and intracellular sides of the membrane. When the re-entrant loops are intact, as observed in the cASIC structures, FaNaC structures, and in domain-swapped conformations of hASIC1a in this study, Arg43 (and equivalent arginines in FaNaC) is shielded by the re-entrant loop and does not face the pore. Thus, this experimental finding suggests that Arg43 must at some point become exposed to the aqueous pore environment. This could plausibly occur in the linear conformation we observed, in which the re-entrant loop is displaced and no longer restricts Arg43 accessibility. While we were cautious in assigning the helical register of TM1 due to local resolution limits, and thus modeled it using a canonical helical geometry, in the linear conformation, Arg43 is positioned just one turn away from facing directly into the pore, unobstructed by the re-entrant loop. This observation offers a potential structural rationale for the accessibility profile seen in the study by Pfister et al. We have added this interpretation in our discussion section lines 380-386: "This result recalls a previous study that link structural features of the linearized TM2 conformation to a functional open state. Cysteine accessibility experiments[29] provide support for an activation mechanism that displaces the re-entrant loop, as seen with the linearized-TM2 conformations. Arg43 has both conformation- and state-dependent exposure to the pore environment, becoming chemically accessible from the pore-facing side upon channel activation and becoming unshielded by the re-entrant loop between domain-swapped and linear conformations."

2. OL and OM conformations are indeed very similar, and it is unclear how selectivity can be achieved in one but not the other. The authors suggest that the changes might be minor and not apparent at this resolution. Alternatively, the structures with linear helices might represent non-selective channels. This would also be consistent with T26V mutant (linear helices in the structure) having lower Na+ selectivity (Sheikh et al., 2021). The authors might consider adding "and reduces ion selectivity" to the line 169. The authors should also discuss the conformation of the selectivity filter in their resting / 'open' / desensitized structures.

We appreciate the reviewer for highlighting this portion. We have now revised the sentence to state, "This mutation was chosen primarily for its ability to increase apparent proton sensitivity in mouse ASIC1a, despite its accompanying reduction in ion selectivity[28]" in lines 167-169.

With the addition of new functional experiments and analysis, we have revised our interpretation accordingly. The updated experimental description and explanation can now be found in lines 247-267, which state:
"Although the O_M and O_L pore profiles are only slightly different (Fig. 4c,g and Extended Data Fig. 5j,k), functional experiments with MitTx-activated hASIC1a suggest a loss of sodium ion selectivity during peak activation (Fig. 4i,j), similar to the reduced selectivity observed in MitTx-activated cASIC1a[10]. This phenomenon suggests perturbation of the selectivity filter, although local resolution limitations in the TMD region (Extended Data Figs. 2d,e and 4a,b) preclude unambiguous modeling of key pore-lining residues and complicate the assignments of O_L and O_M to individual selectivity conditions. However, patch-clamp recordings of ASIC1a-transfected HEK C2 cells reveal a clear shift in reversal potential between the peak and sustained phases of proton activation, indicating reduced sodium selectivity during the later, partially desensitized current (Fig. 4k, l). To test whether this observed shift in reversal potential is due to ion redistribution, as shown for overexpressed P2X receptors[34], or instead reflects a true, state-dependent change in selectivity we conducted patch-clamp recordings of endogenous ASIC currents from HEK cells[35]. As expected, endogenous ASIC currents in HEK cells show slightly increased selectivity, but the recordings from these small endogenous ASIC currents confirm a change in ion selectivity, rather than ion distribution[30,35] (Fig. 4m, n). This behavior could be consistent with a rearrangement of the selectivity filter during activation, or with channels entering multiple conducting states with different desensitization rates. The shared reduction in selectivity during MitTx activation and during the later phase of proton-gated currents could suggest a common structural basis. Because both the MitTx-bound and toxin-free hASIC1a structures adopt a linear pore architecture, this conformation may correspond to the state occupied during the partially desensitized, less-selective phase of proton activation."

We have devoted a paragraph in the discussion section to describe the different conformations of the selectivity filter in lines 412-430:

"The conformation of the GAS motif differs markedly between the domain-swapped and linear architectures, with potential implications for ion selectivity. In the domain-swapped conformations, this selectivity filter region adopts a belt-like geometry, disrupted by a conserved glycine that introduces a helical break. In contrast, in the linear conformation, this region forms a continuous α-helix. Both proton- and MitTx-induced activation have been associated in this study with TM2 linearization, as well as the T26V mutation shifting the resting-state equilibrium toward the linearized conformation; reduced ion selectivity is also associated with MitTx addition (Fig. 4i,j) and pre-TM1 mutations including T26V[28]. Mutations such as T26V could influence selectivity upon channel opening by altering the GAS motif configuration or decreasing the dwell time of TM1 and re-entrant loop residues within the conduction pathway. Although our structural studies have not revealed an alternative activated state exposing these regions to the pore, we have described a functional transition from higher- to lower-selectivity conducting states during a single proton activation event. One explanation for these various structural and functional observations is that O_L and O_M both represent the low-

selectivity open conformation, with C_{T26V} representing a nonconducting state which is biased toward accessing only this open conformation."

3. Both OL and OM maps have prominent non-protein densities partially inserting into the pore (particularly OL map), which were not taken into account when calculating pore diameter. I am struggling with the idea that this would not interfere with ion conduction. Current Figure 4a is misleading, as it does not show just how much these densities are inside the pore. The authors should describe these densities, along with implications for ion conduction, and prepare a new figure clearly demonstrating them, with the surrounding protein density at the same contour.

We thank the reviewer for this important observation. We agree that the non-protein features visible in the OL and OM maps, particularly in the OL dataset, partially extend into the pore and should be described more explicitly. As suggested, we have added figures to show these non-protein features, which clearly illustrates these densities in the context of the surrounding protein architecture. These panels are shown in Extended Data Figure 6. Based on their alkyl chain-like shape observed, these features likely represent co-purified endogenous lipids. We excluded them from our pore diameter measurements to focus on the underlying protein architecture, but we now explicitly describe their presence and discuss their potential functional implications in the revised text lines 207-217 and 232-239:

"Ordered lipid-like features, which wrap the ECD-proximal TMD in maps with both linear and domain-swapped TMDs, also occupy the membrane-embedded portions of the fenestrations in O_L and O_M (Fig. 4a; Extended Data Fig. 6a-e), suggesting a conformation-dependent intrusion of lipids into the pore-lining region. These features extend to the level of the selectivity filter, possibly forming interactions with the conserved Gly444 and Ala445 of the GAS motif.
Structural determinants of channel conductance were analyzed by calculating pore profiles with HOLE[31] (Fig. 4b-h and Extended Data Fig. 6f-h). Although the helical turns were generally clear enough to define pore-lining residues with reasonable confidence, the specific orientations of many side chains could not be determined. Additionally, unmodeled putative lipids did not contribute to the pore profile calculations. However, the general trends in pore profile can be interpreted."

"O_L and O_M are therefore the only conformations with wide fenestrations and relatively unconstricted pores (Fig. 4c,g and Extended Data Fig. 6g,h). The combination of widened lateral fenestrations containing putative lipid tails (Fig. 4a and Extended Data Fig. 6a,b,e) and open pores parallels findings from Bandarupalli et al.[33] In that study, molecular dynamics simulations showed that lipid tails can enter the ASIC3 pore through lateral fenestrations, but only when the channel adopts an open conformation. This state-dependent accessibility supports our interpretation that the conformations captured in the O_L and O_M datasets reflect an open state, one that permits lipid access from the membrane to the pore."

4. The manuscript would benefit from a more extensive discussion on the role of GAS

and HG motives for channel gating / ion selectivity, in the context of the newly solved structures and the proposed activation mechanism with linear helices. The GAS motif refolding completely changes the lower pore profile. Additionally, the HG motif was shown important for ion selectivity (Sheikh et al., 2021) – how is this achieved if in the linear TM conformation the reentrant loop is not lining the pore anymore?

The reviewer makes a good point. Related to our response in comment #2, this is addressed in the discussion section lines 412-430, which states:

"The conformation of the GAS motif differs markedly between the domain-swapped and linear architectures, with potential implications for ion selectivity. In the domain-swapped conformations, this selectivity filter region adopts a belt-like geometry, disrupted by a conserved glycine that introduces a helical break. In contrast, in the linear conformation, this region forms a continuous α-helix. Both proton- and MitTx-induced activation have been associated in this study with TM2 linearization, as well as the T26V mutation shifting the resting-state equilibrium toward the linearized conformation; reduced ion selectivity is also associated with MitTx addition (Fig. 4i,j) and pre-TM1 mutations including T26V[28]. Mutations such as T26V could influence selectivity upon channel opening by altering the GAS motif configuration or decreasing the dwell time of TM1 and re-entrant loop residues within the conduction pathway. Although our structural studies have not revealed an alternative activated state exposing these regions to the pore, we have described a functional transition from higher- to lower-selectivity conducting states during a single proton activation event. One explanation for these various structural and functional observations is that O_L and O_M both represent the low-selectivity open conformation, with C_{T26V} representing a nonconducting state which is biased toward accessing only this open conformation."

5. Please include data processing workflows for every structure, this is the standard in the field.

We have expanded the workflow as suggested, and the updated version is now included as Supplementary Data Figure 2.

Presentation:
1. Figure 4 and Extended Data Figure 5 – the pore profiles are not really informative, the authors could for example only show the pore regions of their structures next to calculated pore diameter plots, and label the residues forming constrictions, which are mentioned in the text.

Figures 4 and now Extended Data Figure 6 have been revised.

Reviewer #3 (Remarks to the Author):

The manuscript by Cahill et al. presents an extensive cryo-EM study of human ASIC1a,

detailing six distinct conformations captured under various pH conditions as well as with pharmacological modulation via MitTx and a T26V mutation. The work provides valuable insights into the structural transitions associated with channel activation and desensitization. The overall analysis is thorough, and the manuscript is clearly written. However, several points need to be addressed to enhance the clarity and rigor of the study:

1. The identification of three distinct desensitized conformations (DDS, DL, DRDS) is intriguing, but their functional relevance remains speculative. Electrophysiological data—such as recovery rates from desensitization and ion selectivity differences between states—are essential to link these structures to specific functional phenotypes. For example, does DRDS represent a metastable intermediate during desensitization, or is it a unique nonconducting state? Additional experiments (e.g., single-channel recordings or kinetic analyses) could help clarify these distinctions.

We appreciate the reviewer's insightful comments and agree that identifying three desensitized conformations (DDS, DL, DRDS) raises important questions about their functional relevance. We also agree that electrophysiological measurements can provide critical support for multiple desensitized states. However, while approaches such as single-channel analysis or detailed kinetic modeling may reveal distinct nonconducting states, they would still not allow unambiguous assignment of specific structural conformations (e.g., DDS vs. DL vs. DRDS) to particular functional phenotypes. Determining these relationships would require extensive structure-guided mutagenesis followed by combined structural and functional analyses, which we view as an important future direction but beyond the scope of the current work.
Nevertheless, to directly address the reviewer's concern, we have conducted new electrophysiological experiments designed to probe whether hASIC1a can transition between kinetically distinct desensitized states. By comparing short versus prolonged exposure to acidic pH followed by brief recovery at high pH, we observed that channels enter a deeply desensitized state under prolonged acidification that does not readily recover, even though recovery is preserved under shorter exposure conditions. This behavior is consistent with at least two kinetically distinct desensitized states in hASIC1a, in agreement with prior work (Li et al., 2012). These new results support the idea that the structural diversity we observe reflects functionally meaningful desensitization pathways.
This new data and interpretation are described in the revised manuscript (lines 299–314):
"The ability of hASIC1a to form multiple kinetically distinct desensitized states has been previously suggested[42]. To explore whether there is direct transition between kinetically distinct states consistent with structural rearrangement, we performed whole-cell patch-clamp electrophysiology experiments in which we varied the duration of channel exposure to low pH before briefly returning to high pH, followed by a second application of low pH. Following relatively short initial acidification (12-14 s) and a 30 ms recovery step in pH 8.5, reapplication of low pH evoked a current amplitude comparable to the control response (Fig. 6f). In contrast, prolonged initial acid exposure (70-80 s) abolished subsequent currents, indicating entry into a desensitized state that was not

readily reversed by brief alkalinization (Fig. 6g). To ensure that the loss of current did not reflect channel rundown, we added an extended second recovery period (5-10 s) at high pH before subsequent acid challenge. Under these conditions, current responses reappeared, confirming that the channels remained functional and could recover given sufficient time at alkaline pH (Fig. 6h). To exclude perfusion artifacts and to ensure that the 30 ms recovery step was not limiting, we conducted similar experiments (with a 60 ms recovery step in pH 8.5) in outside-out excised patches (Fig. 6i-k) and obtained similar results. These findings support the existence of at least two distinct desensitized conformations of hASIC1a with different rates of recovery."

2. The T26V mutation is reported to increase leak currents, which is attributed to the destabilization of the pre-TM1 "re-entrant loop." However, the manuscript lacks direct evidence (such as detailed hydrogen-bonding interactions in CDS versus CT26V or single-channel recordings) to support this claim. The authors should consider molecular dynamics simulations or additional mutagenesis experiments (e.g., comparisons of T26A vs. T26C) to validate the specific role of Thr26 in stabilizing the closed state.

We appreciate the reviewer's suggestion and agree that understanding the role of Thr26 in stabilizing the closed state is important. Importantly, the functional consequences of mutating this residue have already been thoroughly investigated by Sheikh et al. (2021), who demonstrated that substitutions such as T26V, T26C, and T26E disrupt ion selectivity and shift the pH sensitivity leftward, consistent with destabilization of the closed state. In contrast, the T26S mutation, which preserves hydrogen bonding capacity, behaves similarly to wild-type in both selectivity and proton sensitivity. This prior work directly motivated our decision to generate and structurally characterize the T26V mutant, as noted in lines 167-169 of the manuscript: "This mutation was chosen primarily for its ability to increase apparent proton sensitivity in mouse ASIC1a, despite its accompanying reduction in ion selectivity[28]."

We also highlight these findings again in lines 181-185, where we contrast the behavior of T26S and T26C: "The loss of the pre-TM1 "re-entrant loop" at pH 8.5 appears to be a direct consequence of the isosteric T26V mutation, which removes hydrogen-bonding capability. In support of this assignment, the T26S[28] (but not the T26C)[28,29] mutant retains wild-type-like function. Thus, the loss of this single hydrogen-bonding interaction may be sufficiently destabilizing to the "re-entrant loop" configuration of pre-TM1 to induce a conformational shift to C_{T26V}."

While our current study does not include single-channel recordings or hydrogen-bonding analysis, our structural observations in the T26V conformation are fully consistent with the previously reported functional data. Further studies such as molecular dynamics simulations could refine the understanding of these interactions, but we consider this outside the scope of the present work.

3. The discussion briefly touches on ASIC1a's roles in stroke and pain but does not fully explore the therapeutic implications of the observed conformational diversity. For instance, could the stabilization of specific desensitized states (such as DDS) potentially mitigate acidosis-induced neuronal damage? Furthermore, how might the insights

gained from toxin-MitTx interactions inform the design of non-proton agonists? A deeper discussion in this regard would enhance the manuscript's broader impact.

As stated in our response in Reviewer #1's comment #2:
The extracellular domains (ECDs) in our newly resolved structures are largely consistent with those reported in prior studies, with no major rearrangements that would immediately suggest new access points for peptide or chemical modulators. However, a key advance of our study is the observation, at pH 7.5, of multiple, distinct ECD conformations coexisting within the same dataset. To our knowledge, this represents the first structural evidence that ASIC1a can adopt a range of ECD conformations under a single pH condition. This conformational heterogeneity suggests that ASIC1a exists in a dynamic equilibrium between structural states, which may be a fundamental feature of its regulation. Importantly, modulators like PcTx1 bind in a state-dependent manner and alter steady-state desensitization. Our findings therefore provide a structural basis for how such modulators can act selectively by exploiting pre-existing conformational ensembles. Given that PcTx1 has served as a foundation for designing new ASIC-targeting molecules, this insight into the structural plasticity of the ECD may have broad implications for the development of next-generation modulators. We now highlight this point in the revised discussion section lines 357-362:

"Such heterogeneity could explain how state-dependent modulators like PcTx1, which bind selectively and alter steady-state desensitization, exert their effects[44]. Our structural observations provide a mechanistic basis for how such modulators may act by stabilizing specific pre-existing conformations. Given that PcTx1 has served as a foundation for designing new ASIC-targeting compounds[45,46], our findings may inform future efforts to exploit this intrinsic conformational plasticity for therapeutic development."

4. The local resolution limitations in the TMD region (Extended Data Figs. 2d-e, 4a-b) raise concerns about the accuracy of side-chain placements, particularly for key pore-lining residues such as Leu441 and Asp434. A dedicated section discussing potential model uncertainties and how these might affect the mechanistic interpretations would strengthen the manuscript.

We thank the reviewer for raising this important point. We fully acknowledge the limitations imposed by local resolution in the TMD region. For this reason, we have been careful throughout the manuscript to avoid overinterpretation and to explicitly acknowledge the associated uncertainties. For example, in lines 214-216, we state:
"Although the helical turns were generally clear enough to define pore-lining residues with reasonable confidence, the specific orientations of many side chains could not be determined."
Lines 250-253:
"This phenomenon suggests perturbation of the selectivity filter, although local resolution limitations in the TMD region (Extended Data Figs. 2d,e and 4a,b) preclude unambiguous modeling of key pore-lining residues and complicate the assignments of O_L and O_M to individual selectivity conditions."

To further clarify these limitations, we have added a dedicated statement to the *Potential Methodological Constraints* section lines 459-465: “We also note that local resolution in the transmembrane region imposes limitations on the accuracy of pore radius calculations (Extended Data Figs. 2d,e and 4a,b). While the backbone trace and helical register are generally well resolved, side-chain orientations remain ambiguous in several regions, especially near the lower pore. The apparent intrusion of lipid-like map features into the pore region (Extended Data Fig. 6a-e) may also alter the effective pore radius and local chemical environment from what the protein models would suggest. As a result, precise interpretation of pore geometry and ion conduction pathways should be approached with caution.”

This addition helps to explicitly address the limitations in structural precision that may affect mechanistic interpretations of the pore architecture.

We are grateful to the reviewers and editor for their insightful feedback, which has substantially improved both the clarity and impact of our manuscript.

Reviewer #1:
Remarks to the Author:
My queries about the earlier version of this manuscript have now been addressed. I believe this paper is appropriate for publication in Nature Structural and Molecular Biology.

We thank the reviewer for their valuable comments and suggestions.

Reviewer #3:
Remarks to the Author:
The authors have addressed all my concerns, and the manuscript is acceptable for publication.

We thank the reviewer for their valuable comments and suggestions.
